# Skeletal Editing of Mechanically Interlocked Molecules: Nitrogen Atom Deletion from Crown Ether-Dibenzylammonium Rotaxanes

**DOI:** 10.1021/jacs.4c09066

**Published:** 2024-10-21

**Authors:** Maxime Gauthier, Jessica B. M. Whittingham, Avantika Hasija, Daniel J. Tetlow, David A. Leigh

**Affiliations:** † Department of Chemistry, University of Manchester, Oxford Road, Manchester M13 9PL, U.K.; ‡ School of Chemistry and Molecular Engineering, East China Normal University, Shanghai 200062, China

## Abstract

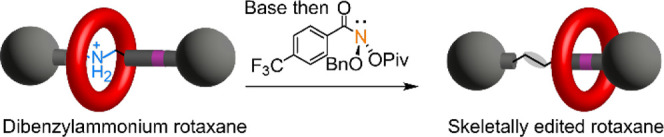

Removing the nitrogen atom from secondary amines while simultaneously linking the remaining fragments is a powerful form of late-stage skeletal editing. Here, we report its use for the deletion of the nitrogen atom of the dibenzylammonium template used to assemble crown ether rotaxanes. The reaction uses an anomeric amide that activates secondary amines to generate a carbon–carbon bond that replaces the amine nitrogen. Despite the potential for dethreading of the intermediate diradical pair, the nitrogen atom was successfully deleted from a series of rotaxane axles as long as the macrocycle could access coconformations that did not inhibit the reaction of the amine group. The skeletally edited interlocked molecules were obtained directly from the parent crown ether-dibenzylammonium rotaxanes in modest yields (23–36%) and characterized by NMR spectroscopy, mass spectrometry, and X-ray crystallography. One skeletally edited rotaxane shows a network of weak CH···O hydrogen bonds between the crown ether and benzylic methylene groups of the axle in the solid state, in place of the crown ether-ammonium binding motif used to assemble the parent, unedited, rotaxane.

## Introduction

A structural limitation in the types of mechanically interlocked molecules that can be accessed by conventional (“passive”) template synthesis is that specific recognition elements are required on both components, which remain in the interlocked product.^1^ Alternative synthetic strategies that address this shortcoming include active template synthesis^2^ (catalysis of a bond-forming reaction through the cavity of a macrocycle), cleavable molecular scaffolds,^3^ and mechanical interlocking auxiliaries^4^ (multistep reaction sequences that remove the template site following translocation of the macrocycle to another site on the thread). These approaches expand the structural diversity possible for interlocked molecules by enabling the synthesis of structures where at least one component is traceless with regards to the process used to assemble the threaded architecture.^4j^

Here we report on another way to expand the structural diversity of interlocked molecules, namely late-stage modification of crown ether-dibenzylammonium rotaxanes,^1,5^ one of the most common and easily accessible classes of rotaxanes. Skeletal editing^6^ is a powerful approach for modifying already structurally complex intermediates in organic synthesis. The various processes explored to date include insertion (where insertion of carbon or nitrogen causes ring expansion),^7^ atom swapping (typically changing a carbon atom for either oxygen or nitrogen)^8^ and deletion (where an atom is completely removed from a molecule).^9,10^

In 2021, the Levin group reported the use of anomeric amide **1** as an effective reagent for deleting nitrogen from a range of amines.^10^ The protocol circumvents the need for potentially explosive reagents, such as low molecular weight azides,^11^ previously used for such transformations. The reported scope^10^ for nitrogen deletion with **1** includes dibenzylamine which, when protonated, is a popular axle motif for promoting^1,5,12^ crown ether rotaxane formation.

**Figure 1:**
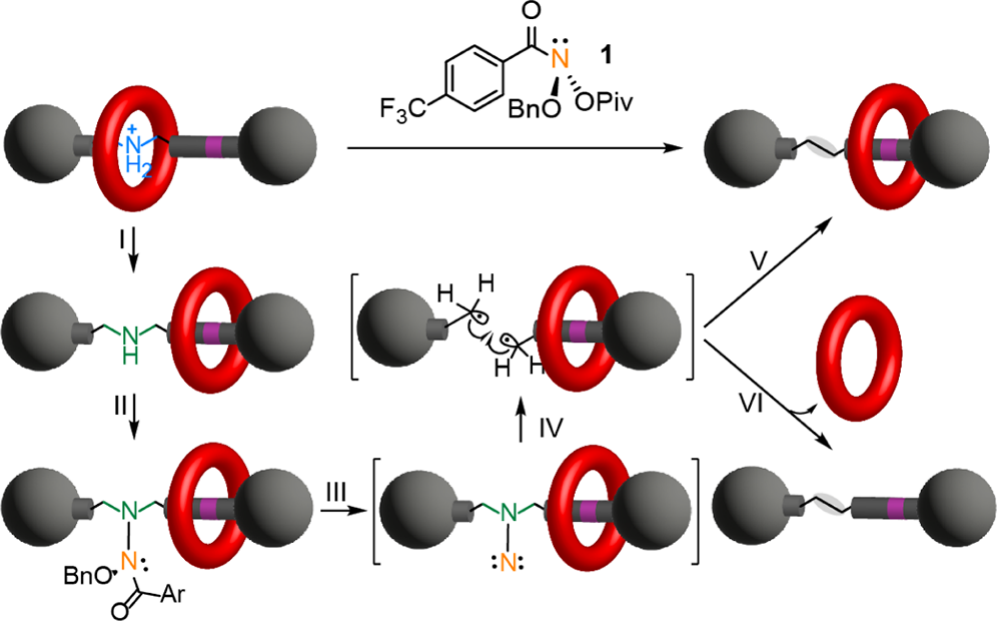
Skeletal editing of crown ether-dibenzylammonium rotaxanes by nitrogen deletion. Mechanistic outline: I. Deprotonation with base. II. Reaction of the secondary amine with anomeric amide **1**. III. Formation of a reactive isodiazene intermediate. IV. Extrusion of molecular nitrogen and formation of a diradical pair. V. If recombination of the diradical pair to form a C–C bond is faster than dethreading from the pseudorotaxane radical, the nitrogen-deleted rotaxane is formed. VI. If dethreading is faster than radical recombination, or one of the radical fragments leaves the solvent cage, then dethreaded products result.

Chemically deleting the template from a rotaxane axle is challenging because (i) the macrocycle generally remains associated with the template site because of intercomponent binding interactions that persist after assembly of the rotaxane, and (ii) transiently cleaving the axle would allow the ring to dethread, especially if the binding site had been deleted. Nevertheless, we reasoned that crown ether dibenzylammonium-axle rotaxanes were suitable candidates for investigating in this regard for two reasons (Figure 1): First, the two benzyl groups would stabilize the radicals on each fragment of the axle formed during the deletion process;^13^ and, second, under basic conditions the crown ether would not remain tightly bound to the deprotonated template (i.e., amine) site and, with sufficient space on the axle, the ring might spend sufficient time away from the amine to not sterically hinder^14^ its reaction with the anomeric amide.

We envisaged a process such as that shown in Figure 1: Deprotonation of the dibenzylammonium group (shown in blue) by base (Figure 1, step I), frees the crown ether (red) from strong hydrogen bonding to the original template site (green). The axle amine should then be sufficiently unhindered to be able to displace the pivalate from the electrophilic nitrogen atom of the anomeric amide **1** (Figure 1, step II). An *N*-alkoxyhydrazide is generated, which undergoes a reductive elimination-like process to generate an ester byproduct and a highly reactive isodiazene intermediate (Figure 1, step III). The isodiazene decomposes to generate dinitrogen and C-centered benzylic radicals on both axle fragments (Figure 1, step IV). The two radicals then undergo what should in principle be a fast, in-cage, reaction to form a C–C bond, reconnecting the axle without the nitrogen atom. If the radical recombination is faster than dethreading of the macrocycle from the transiently broken axle, then the rotaxane architecture would be retained (Figure 1, step V). However, if dethreading is faster than C–C bond formation, or if one of the radicals escapes the solvent cage, then unthreaded products would result (Figure 1, step VI).

## Results and Discussion

**Figure 2:**
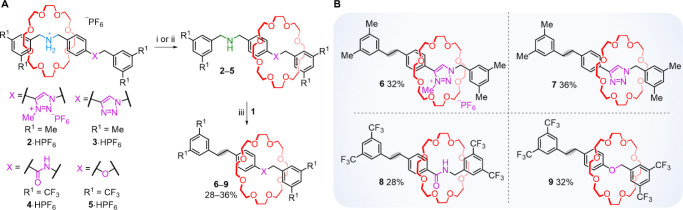
(A) Skeletal editing of dibenzylammonium rotaxanes by nitrogen atom deletion from the axle template with Levin’s anomeric amide **1**.^10^ Reagents and conditions: (i) for **2**·HPF_6_, **3**·HPF_6_, and **5**·HPF_6_: polymer-supported 2-*tert*-butylimino-2-diethylamino-1,3-dimethylperhydro-1,3,2-diazaphosphorine (BEMP) (2–5 equiv), CD_3_CN, RT, 2 h, quant. (ii) For **4**·HPF_6_: polymer-supported 1,8-diazabicyclo[5.4.0]undec-7-ene (DBU) (6 equiv), CD_3_CN, RT, 2 h, quant. (iii) **1** (2 equiv), THF, RT, overnight, 28–36%. (B) Rotaxanes **6**–**9** generated by nitrogen deletion.

The mechanism of nitrogen deletion requires stabilization of both alkyl radical fragments,^10a^ so we used dibenzylamine axles to prepare an initial series of crown ether rotaxanes (**2**–**5**) for investigation (Figure 2). Triazole rotaxane **3** was synthesized through a standard ammonium-promoted threading-and-stoppering process^1^ with Cu(I)-catalyzed alkyne–azide cycloaddition (CuAAC) click chemistry (Supporting Information).^15^ Alkylation of **3**·HPF_6_ with MeI, followed by anion exchange, afforded rotaxane **2**·HPF_6_. Rotaxane salts **4**·HPF_6_ and **5**·HPF_6_ were assembled directly by metal-free active template synthesis (Supporting Information). The ammonium groups of each rotaxane were deprotonated with polymer-supported 2-*tert*-butylimino-2-diethylamino-1,3-dimethylperhydro-1,3,2-diazaphosphorine (BEMP) or 1,8-diazabicyclo(5.4.0)undec-7-ene (DBU) to generate the corresponding amine-axle rotaxanes, **2**–**5**.

**Figure 3:**
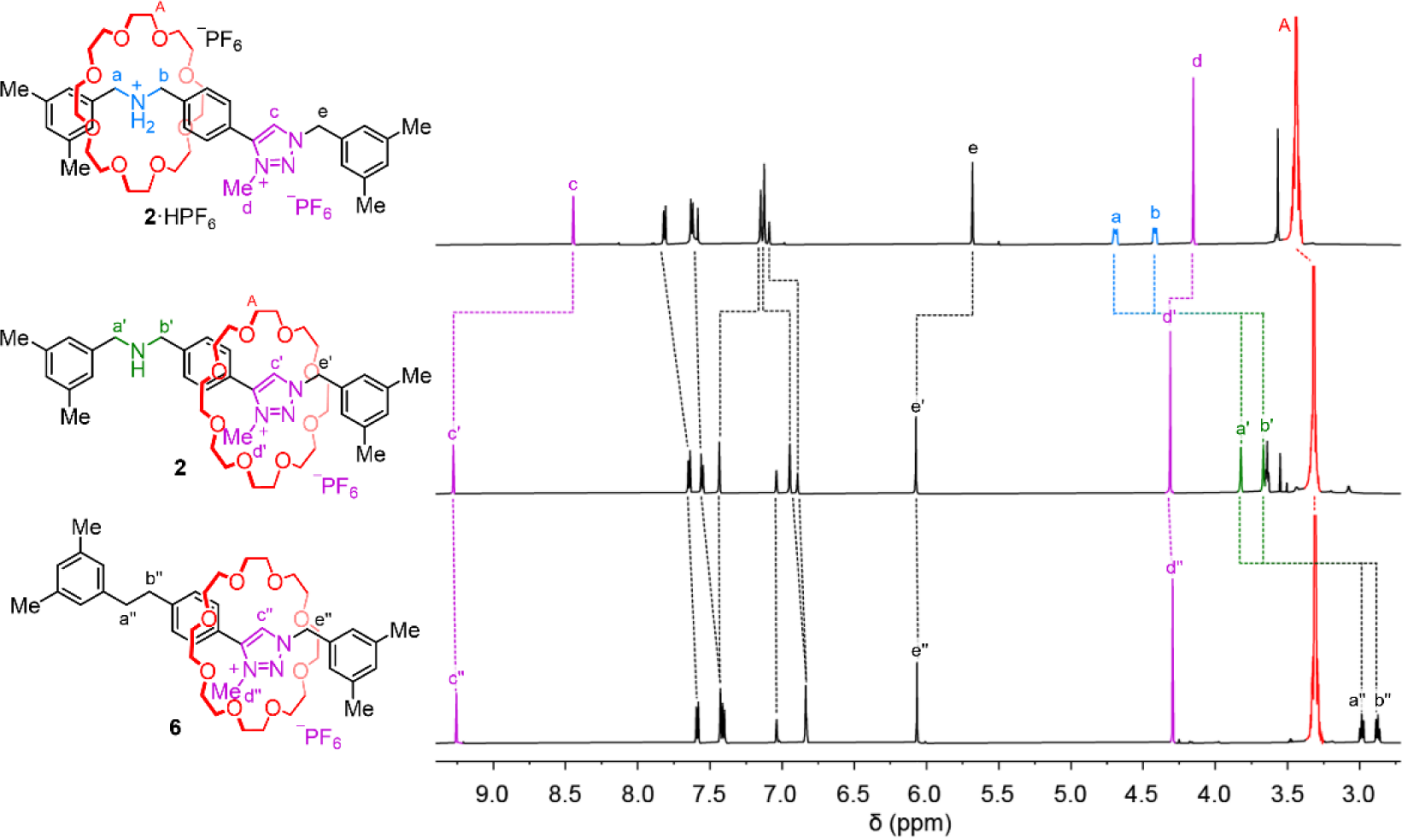
^1^H NMR spectra (CD_3_CN, 600 MHz, 298 K) of rotaxanes **2**·HPF_6_ (top), **2** (middle), and **6** (bottom).

We first investigated the nitrogen deletion reaction on rotaxane **2**, because axle methyl triazolium groups have a binding affinity for threaded crown ethers in-between that of amine and ammonium groups^16,17^ and so the macrocycle should not preclude the amine from reacting with **1** while binding to the methyl triazolium will slow dethreading of the pseudorotaxane benzyl radical intermediate. Indeed, ^1^H NMR spectroscopy confirmed that the crown ether in rotaxane **2** is mostly positioned over the methyl triazolium site in CD_3_CN (Figure 3, top and middle), leaving the axle amine group sterically unhindered. Hydrogen atoms H_a′_ and H_b′_ of the benzylamine group are upfield in **2** compared to **2**·HPF_6_ (Δδ = −0.87 ppm and −0.75 ppm, respectively). In contrast, H_c′_ and H_d′_ of the methyl triazolium group, and the adjacent benzylic hydrogens H_e’_ are shifted downfield (Δδ = 0.83, 0.16, and 0.39 ppm, respectively), due to CH···O hydrogen bonding with the crown ether.

Rotaxane **2** was treated with anomeric amide **1** in tetrahydrofuran (THF) at room temperature overnight. The reaction afforded a complex mixture of products by thin layer chromatographic (TLC) analysis. However, flash column chromatography allowed the isolation of nitrogen-deleted rotaxane **6** in 32% yield. High resolution mass spectrometry (HRMS) confirmed the molecular mass of **6** (*m*/*z* = 762 [M]^+^) and ^1^H NMR spectroscopy showed a new set of resonances at 2.99 and 2.87 ppm corresponding to diarylethane protons H_a″_ and H_b″_ (Figure 3, bottom spectrum). The chemical shifts of H_c″_, H_d″_ and H_e″_ of the methyl triazolium site are essentially unchanged with respect to the analogous protons in **2**, indicating the overall position of the macrocycle is similar in the rotaxane before and after nitrogen deletion. In addition to **2**, some free 24-crown-8 was also produced, indicating that some dethreading does occur during the nitrogen deletion reaction. However, we were unable to identify the structures of the corresponding cleaved axle fragments.

Following the successful nitrogen deletion from **2**, we next explored whether or not the macrocycle needs to be held in an axle position distant from the amine group, as it is in the methyl triazolium rotaxane. Threaded crown ethers have a much weaker affinity for neutral triazole groups than charged methyl triazolium groups on rotaxane axles. The ^1^H NMR of **3** and the corresponding thread (Section S4.2) shows modest shifts in the resonances of protons near the amine or the triazole group indicating that the macrocycle is not localized to either site.^18^ Reaction of **3** with **1**, afforded nitrogen-deleted rotaxane **7** in 36%

In order to assess if macrocycle-axle binding in the pseudorotaxane benzyl radical intermediate has a significant effect on the amount of dethreading that occurs during the deletion reaction, we applied the reaction to dibenzylamine rotaxanes **4** and **5**, which feature amide and ether groups, respectively.^19^ Crown ether-amide hydrogen bonds have been observed in rotaxanes in the absence of alternative axle binding sites,^20^ while crown ethers would be expected to bind only very weakly to a benzyl ether group. Carrying out the nitrogen deletion reaction with **1** on **4** and **5** led to **8** and **9** in 28% and 32% yield, respectively, similar values to those obtained for **6** and **7**. This is consistent with radical recombination to form the C–C bond being much faster than dethreading within the solvent cage, suggesting that the 24C8 released in the reaction mainly arises as a result of benzyl radicals escaping the solvent cage before recombination.

**Figure 4:**
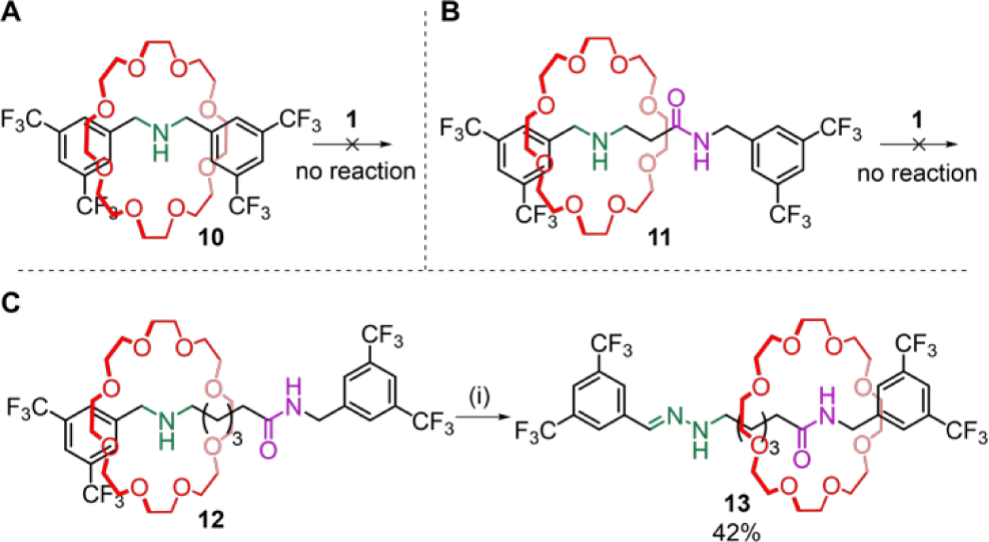
Limitations in the scope for nitrogen deletion of crown ether-amine rotaxanes. Steric hindrance of the dibenzylamine by (A) lack of room on the axle for the macrocycle to move away from the amine, or (B) having a secondary binding site close to the amine, the proximity of the macrocycle inhibiting reaction of the axle amine with anomeric amide **1**. (C) In the absence of groups on both axle fragments to stabilize the radicals formed in step IV (Figure 1), hydrazone formation occurs instead of nitrogen deletion. Reagents and conditions: (i) **1** (3 equiv), THF, RT, overnight, 42%.

We next explored the efficacy of the nitrogen deletion reaction on more compact^21^ dibenzylamine rotaxanes (**10** and **11**, Figure 4) which have less room on the axle away from the amine for the macrocycle to occupy. Neither rotaxane **10** or **11** reacted with **1** under our reaction conditions (Figure 4A,B), in line with the limitations due to steric hindrance noted in the original nitrogen deletion report by Levin and coworkers.^10a^

To investigate whether it was necessary to stabilize the radicals on both axle fragments (IV, Figure 1) in order to form the axle C–C bond after nitrogen deletion with **1**, we prepared monobenzylamine rotaxane **12**. Treatment of **12** with **1** afforded hydrazone **13** in 42% yield with no evidence of the nitrogen-deleted rotaxane. The hydrazone apparently results^10a^ from rearrangement of the isodiazene intermediate (III, Figure 1) formed during the deletion reaction (Figure 4C and Section S5.1). Although the template nitrogen atom is not deleted in rotaxane **13** the reaction does represent a novel method for forming hydrazone-terminated crown ether rotaxanes that should be able to undergo stopper-exchange by dynamic covalent chemistry.^22^

**Figure 5:**
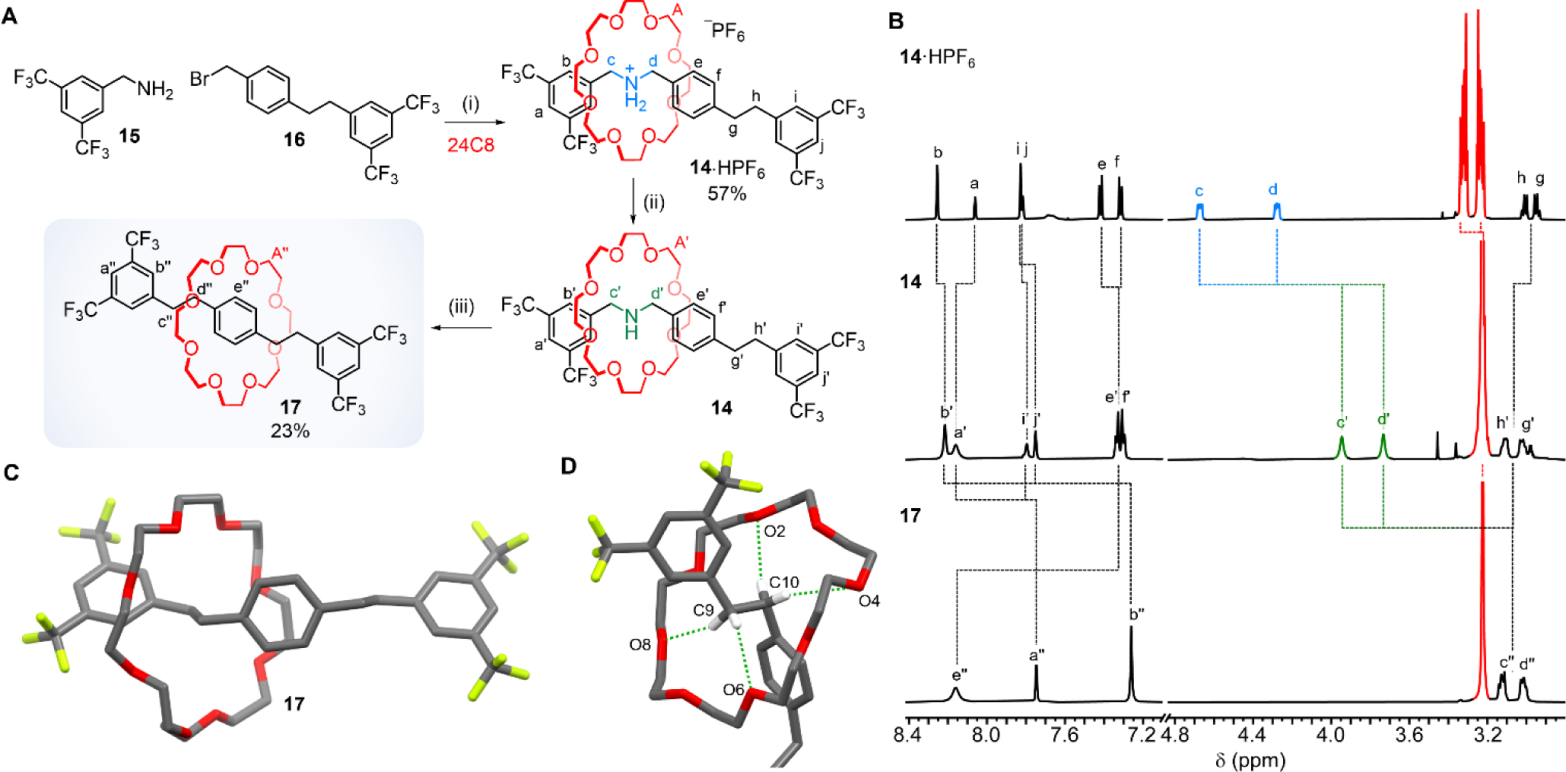
(A) Metal-free active template synthesis of **14**·HPF_6_ (a rotaxane with an extended axle and no secondary binding site for the macrocycle) and its subsequent nitrogen deletion to give symmetrical [2]rotaxane **17** with an all-carbon backbone. Reagents and conditions: (i) **15** (2 equiv), **16** (1 equiv), 24C8 (2 equiv), toluene, RT, 21 h, then NH_4_PF_6_ (6 equiv), CHCl_3_/H_2_O (1:1), 57%. (ii) Polymer-supported 2-*tert*-butylimino-2-diethylamino-1,3-dimethylperhydro-1,3,2-diazaphosphorine (BEMP) (5 equiv), CD_3_CN, RT, 2 h, quant. (iii) **1** (2 equiv), THF, RT, overnight, 23%. (B) ^1^H NMR of **14**·HPF_6_ (top), **14** (middle), and **17** (bottom) (CD_3_CN, 600 MHz, 298 K). (C) X-ray crystal structure of **17**. (D) Side view to show the CH···O hydrogen bonding between the macrocycle and diarylethane region of the thread. Hydrogen bond lengths: O2···HC10 2.759 Å, O4···HC10 2.617 Å, O6···HC9 2.413 Å, and O8···HC9 2.575 Å. Hydrogen bond angles: O2···HC10 165.3°, O4···HC10 170.8°, O6···HC9 147.4°, and O8···HC9 165.0°. Carbon, gray; oxygen, red; hydrogen, white; fluorine, yellow. Hydrogen bonds are shown in green. Additional hydrogen atoms and solvent molecules are omitted for clarity.

Finally, we prepared dibenzylamine [2]rotaxane **14**·HPF_6_ in 57% yield by metal-free active template^19^ synthesis from **15**, **16** and 24-crown-8 (Figure 5A). Deprotonation of **14**·HPF_6_ with polymer-supported BEMP was accompanied by an upfield shift of protons H_c_ and H_d_ in the ^1^H NMR spectrum of **14** (Figure 5B, top and middle). Treatment of **14** with **1** afforded the nitrogen-deleted, symmetrical axle, rotaxane **17** in 23% yield.

Single crystals of **17** suitable for investigation by X-ray crystallography were obtained from slow evaporation of a saturated solution of **17** in diethyl ether. The solid-state structure of **17** confirms the rotaxane architecture is maintained following the deletion of the nitrogen atom from **14**, and shows the macrocycle sited around one of the diarylethane spacers, with which it forms a network of CH···O hydrogen bonds (Figure 5D). A comparison of the ^1^H NMR spectra of **17** with **14** and **14**·HPF_6_ suggests that these intercomponent CH···O interactions are very weak in CD_3_CN solution.

## Conclusions

Nitrogen deletion from the axle template used to assemble crown ether-dibenzylammonium rotaxanes provides a direct and simple route to structurally diverse crown ether rotaxanes that are not currently accessible by other synthetic strategies. The one-step transformation is tolerant to other functional groups (e.g., amides, ethers, triazoles, triazolium salts, etc.), and the fast rate of C–C bond formation means that strong intercomponent binding interactions are unnecessary to avoid macrocycle dethreading of the radical pseudorotaxane intermediate. However, stabilization of the radicals on both axle fragments is required with this reagent system,^10a^ although potentially not with others,^23^ along with sufficient space on the axle for the amine to be able to react with the anomeric amide without being impeded by the macrocycle. The modest yields of editing (23–36%) reflect the yields obtained (often 40–65%^10a^ with conventional (i.e., not mechanically interlocked) amine substrates), with the added possibility of dethreading occurring from radical intermediates escaping from the solvent cage before recombination occurs.

In the absence of the template site, the interlocking of the components enables weak intercomponent interactions to persist and be characterized in the edited rotaxanes, such as CH···O hydrogen bonding between the crown ether and benzylic methylene groups in the X-ray crystal structure of **17**.

While it was convenient for us to use rotaxanes containing 24-crown-8 in this study because of its size complementarity with readily available 3,5-disubstituted-benzylamine stoppers, the functional group tolerance of the nitrogen-deletion reaction^10a^ suggests that this methodology should be applicable to rotaxanes incorporating other sized crown ethers (with appropriately sized stoppers) as well as rotaxanes based on other ammonium-binding macrocycles, such as cucurbiturils,^24^ cyclic amides^25^ and calixarenes.^26^ We anticipate that this and other forms of skeletal editing will provide efficacious strategies to catenanes and rotaxanes that are difficult or impossible to prepare by other synthetic methods.^10b^

## Supporting Information Available

Experimental procedures, synthesis, and characterization data; NMR, mass spectrometry, and X-ray crystallography data (PDF)

## Notes

While this manuscript was undergoing peer review we found that *O*-diphenylphosphinylhydroxylamine (DPPH), a very recently reported^23^ alternative nitrogen deletion reagent, is also effective for nitrogen atom deletion from crown ether-dibenzylammonium rotaxanes. In a typical example experiment, treatment of rotaxane **5**·HPF_6_ with polymer-supported BEMP (5 equiv.) in CH_3_CN, followed by DPPH (5 equiv.), K_2_CO_3_ (2 equiv.) in THF/H_2_O (1:1) smoothly afforded nitrogen-deleted rotaxane **9** in 43% yield (see Supporting Information, Section 6).

## References

[ref1] Bruns C. J.; Stoddart J. F. The Nature of the Mechanical Bond: From Molecules to Machines; John Wiley & Sons: Hoboken, NJ, 2017.

[ref2] aAucagne V.; Hänni K. D.; Leigh D. A.; Lusby P. J.; Walker D. B. Catalytic “click” rotaxanes: A substoichiometric metal-template pathway to mechanically interlocked architectures. J. Am. Chem. Soc. 2006, 128, 2186–2187. 10.1021/ja056903f. 16478152

[ref3] aSegawa Y.; Kuwayama M.; Hijikata Y.; Fushimi M.; Nishihara T.; Pirillo J.; Shirasaki J.; Kubota N.; Itami K. Topological molecular nanocarbons: All-benzene catenane and trefoil knot. Science 2019, 365, 272–276. 10.1126/science.aav5021. 31320538

[ref4] aHannam J. S.; Lacy S. M.; Leigh D. A.; Saiz C. G.; Slawin A. M. Z.; Stitchell S. G. Controlled submolecular translational motion in synthesis: A mechanically interlocking auxiliary. Angew. Chem., Int. Ed. 2004, 43, 3260–3264. 10.1002/anie.200353606. 15213949

[ref5] aKolchinski A. G.; Busch D. H.; Alcock N. W. Gaining control over molecular threading: Benefits of second coordination sites and aqueous–organic interfaces in rotaxane synthesis. J. Chem. Soc., Chem. Commun. 1995, 1289–1291. 10.1039/C39950001289.

[ref6] aJurczyk J.; Woo J.; Kim S. F.; Dherange B. D.; Sarpong R.; Levin M. D. Single-atom logic for heterocycle editing. Nat. Synth. 2022, 1, 352–364. 10.1038/s44160-022-00052-1. 35935106 PMC9355079

[ref7] aDherange B. D.; Kelly P. Q.; Liles J. P.; Sigman M. S.; Levin M. D. Carbon atom insertion into pyrroles and indoles promoted by chlorodiazirines. J. Am. Chem. Soc. 2021, 143, 11337–11344. 10.1021/jacs.1c06287. 34286965 PMC8343525

[ref8] aLuu Q. H.; Li J. A C-to-O atom-swapping reaction sequence enabled by Ni-catalyzed decarbonylation of lactones. Chem. Sci. 2022, 13, 1095–1100. 10.1039/D1SC06968C. 35211275 PMC8790783

[ref9] aHsueh S.-Y.; Ko J.-L.; Lai C.-C.; Liu Y.-H.; Peng S.-M.; Chiu S.-H. A Metal-Free “Threading-Followed-by-Shrinking” Protocol for Rotaxane Synthesis. Angew. Chem., Int. Ed. 2011, 50, 6643–6646. 10.1002/anie.201101524. 21648035

[ref10] aKennedy S. H.; Dherange B. D.; Berger K. J.; Levin M. D. Skeletal editing through direct nitrogen deletion of secondary amines. Nature 2021, 593, 223–227. 10.1038/s41586-021-03448-9. 33981048

[ref11] Zou X.; Zou J.; Yang L.; Li G.; Lu H. Thermal rearrangement of sulfamoyl azides: Reactivity and mechanistic study. J. Org. Chem. 2017, 82, 4677–4688. 10.1021/acs.joc.7b00308. 28414236

[ref12] Pedersen C. J. Cyclic polyethers and their complexes with metal salts. J. Am. Chem. Soc. 1967, 89, 7017–7036. 10.1021/ja01002a035.

[ref13] aWright J. S.; Shadnia H.; Chepelev L. L. Stability of carbon-centered radicals: Effect of functional groups on the energetics of addition of molecular oxygen. J. Comput. Chem. 2009, 30, 1016–1026. 10.1002/jcc.21124. 18825692

[ref14] aParham A. H.; Windisch B.; Vögtle F. Chemical reactions in the axle of rotaxanes – steric hindrance by the wheel. Eur. J. Org. Chem. 1999, 1999, 1233–1238. 10.1002/(SICI)1099-0690(199905)1999:5<1233::AID-EJOC1233>3.0.CO;2-Q.

[ref15] aTornøe C. W.; Christensen C.; Meldal M. Peptidotriazoles on solid phase: [1,2,3]-triazoles by regiospecific copper(I)-catalyzed 1,3-dipolar cycloadditions of terminal alkynes to azides. J. Org. Chem. 2002, 67, 3057–3064. 10.1021/jo011148j. 11975567

[ref16] Coutrot F. A focus on triazolium as a multipurpose molecular station for pH-Sensitive interlocked crown-ether-based molecular machines. ChemistryOpen 2015, 4, 556–576. 10.1002/open.201500088. 26491633 PMC4608521

[ref17] aErbas-Cakmak S.; Fielden S. D. P.; Karaca U.; Leigh D. A.; McTernan C. T.; Tetlow D. J.; Wilson M. R. Rotary and linear molecular motors driven by pulses of a chemical fuel. Science 2017, 358, 340–343. 10.1126/science.aao1377. 29051374

[ref18] Gauthier M.; Fournel-Marotte K.; Clavel C.; Waelès P.; Laurent P.; Coutrot F. An interlocked figure-of-eight molecular shuttle. Angew. Chem., Int. Ed. 2023, 62, e202310643 10.1002/anie.202310643. 37594476

[ref19] aHübner G. M.; Gläser J.; Seel C.; Vögtle F. High-yielding rotaxane synthesis with an anion template. Angew. Chem. Int. Ed. 1999, 38, 383–386. 10.1002/(SICI)1521-3773(19990201)38:3<383::AID-ANIE383>3.0.CO;2-H. 29711647

[ref20] Riss-Yaw B.; Morin J.; Clavel C.; Coutrot F. How secondary and tertiary amide moieties are molecular stations for dibenzo-24-Crown-8 in [2]rotaxane molecular shuttles?. Molecules 2017, 22, 2017 10.3390/molecules22112017. 29160822 PMC6150268

[ref21] Power M. J.; Morris D. T. J.; Vitorica-Yrezabal I. J.; Leigh D. A. Compact rotaxane superbases. J. Am. Chem. Soc. 2023, 145, 8593–8599. 10.1021/jacs.3c01202. 37039157 PMC10119927

[ref22] Borodin O.; Shchukin Y.; Robertson C. C.; Richter S.; von Delius M. J. Am. Chem. Soc. 2021, 143, 16448–16457. 10.1021/jacs.1c05230. 34559523 PMC8517971

[ref23] Guo T.; Li J.; Cui Z.; Wang Z.; Lu H. C(sp3)–C(sp3) bond formation through nitrogen deletion of secondary amines using O-diphenylphosphinylhydroxylamine. Nat. Synth. 2024, 3, 913–921. 10.1038/s44160-024-00559-9.

[ref24] aKim K. Mechanically interlocked molecules incorporating cucurbituril and their supramolecular assemblies. Chem. Soc. Rev. 2002, 31, 96–107. 10.1039/a900939f. 12109209

[ref25] aAucagne V.; Leigh D. A.; Lock J. S.; Thomson A. R. Rotaxanes of Cyclic Peptides. J. Am. Chem. Soc. 2006, 128, 1784–1785. 10.1021/ja057206q. 16464065

[ref26] aTalotta C.; Gaeta C.; Neri P. Stereoprogrammed Direct Synthesis of Calixarene-Based [3]Rotaxanes. Org. Lett. 2012, 14, 3104–3107. 10.1021/ol3011997. 22668501

