# Supplementary material for: Skeletal Editing of Mechanically Interlocked Molecules: Nitrogen Atom Deletion from Crown Ether-Dibenzylammonium Rotaxanes
Source: J Am Chem Soc. 2024 Oct 21;146(43):29496–502. doi: 10.1021/jacs.4c09066 (PMC11528408; doi:10.1021/jacs.4c09066)
Supplement: Supplementary file 1 — ja4c09066_si_001.pdf [file ja4c09066_si_001.pdf]

-Supporting Information-

Skeletal Editing of Mechanically Interlocked Molecules: Nitrogen Atom Deletion from Crown Ether-Dibenzylammonium Rotaxanes

Maxime Gauthier,[1] Jessica B. M. Whittingham,[1] Avantika Hasija,[1] Daniel J. Tetlow[1] and David A. Leigh[1,2]*

[1]Department of Chemistry, University of Manchester, Oxford Road, Manchester M13 9PL, UK

[2]School of Chemistry and Molecular Engineering, East China Normal University, 200062 Shanghai, China.

Email: david.leigh@manchester.ac.uk

Table of Contents

1. Abbreviations 2
2. General information 2
3. Experimental data 3
 3.1. Synthetic schemes 3
 3.2. Experimental procedures and characterization data 6
4. ^{1}H NMR comparison of protonated, deprotonated, and skeletally edited rotaxanes 19
 4.1. ^{1}H NMR comparison of rotaxanes **2**·HPF_6, **2**, **6** and thread **S11** 19
 4.2. ^{1}H NMR comparison of rotaxanes **3**·HPF_6, **3**, **7** and thread **S9** 20
 4.3. ^{1}H NMR comparison of rotaxanes **4**·HPF_6, **4** and **8** 21
 4.4. ^{1}H NMR comparison of rotaxanes **5**·HPF_6, **5** and **9** 22
 4.5. ^{1}H NMR comparison of rotaxanes **14**·HPF_6, **14** and **17** 23
5. Limitations 24
 5.1. Summary 24
 5.2. Synthetic schemes 25
 5.3. Experimental procedures and characterization data 25
 5.4. ^{1}H NMR comparison of rotaxanes **12**·HPF_6, **12** and **13** 28
6. Comparison of Nitrogen Deletion Reagents 1 & 18 29
 6.1. Synthetic scheme 29
 6.2. Experimental procedure 29
 6.3. ^{1}H NMR comparison of crude reaction mixtures and the isolated [2]rotaxane 30
7. X-Ray Crystallography 31
8. NMR spectra 34

1. Abbreviations

AcOH: acetic acid; APCI-MS: atmospheric-pressure chemical ionization mass spectroscopy; BEMP: 2-tert-Butylimino-2-diethylamino-1,3-dimethylperhydro-1,3,2-diazaphosphorine; Boc_2O: di-*tert*-butyl decarbonate; DBU: 1,8-diazabicyclo [5.4.0]undec-7-ene; DMAP: 4-dimethylaminopyridine; DMF: *N*,*N*-dimethylformamide; EDC·HCl: N-ethyl-N’-(3-dimethylaminopropyl)carbodiimide hydrochloride; EDTA: ethylenediamine tetraacetic acid; eq.: equivalent; EtOAc: ethyl acetate; ESI-MS: electrospray ionization mass spectrometry; h: hours; HRMS: high resolution mass spectrometry; M: mol/L; min.: minute NEt_3: triethylamine; NMR: nuclear magnetic resonance; quant.: quantitative; RT: room temperature; STAB: sodium triacetoxyborohydride; THF: tetrahydrofuran; w/w: weight per weight; 24C8: 24-crown 8-ether.

2. General information

Unless stated otherwise, reagents were obtained from commercial sources and used without purification. Unless stated otherwise, all reactions were carried out in anhydrous solvents and under an N_2 atmosphere. Room temperature (RT) reactions were carried out between 18–25 °C. Anhydrous solvents were obtained by passing the solvent through an activated alumina column on a Phoenix SDS (solvent drying system; JC Meyer Solvent Systems, CA, USA). Solvents were sparged with a balloon filled with argon gas bubbling through. ^{1}H NMR spectra were recorded on a Bruker Avance III instrument with an Oxford AS600 magnet equipped with a cryoprobe [5 mm CPDCH ^{13}C-$^{1}H/D$] (600 MHz) at 298 K. Chemical shifts are reported in parts per million (ppm) from high to low frequency using the residual solvent peak as the internal reference ($CDCl_3$ = 7.26 ppm and CD_3CN = 1.94 ppm). All ^{1}H resonances are reported to the nearest 0.01 ppm. The multiplicity of ^{1}H signals are indicated as: s = singlet; d = doublet; t = triplet; q = quartet; quint = quintet; m = multiplet; br = broad; or combinations thereof. Coupling constants (J) are quoted in Hz and reported to the nearest 0.1 Hz. Where appropriate, averages of the signals from peaks displaying multiplicity were used to calculate the value of the coupling constant. ^{13}C NMR spectra were recorded on the same spectrometer at 298 K with the central resonance of the solvent peak as the internal reference ($CDCl_3$ = 77.16 ppm and CD_3CN = 1.32 ppm). All ^{13}C resonances are reported to the nearest 0.01 ppm to aid in the differentiation of closely resolved signals. DEPT, COSY, HSQC and HMBC experiments were used to aid structural determination and spectral assignment. Fully characterized compounds were chromatographically homogeneous. Flash column chromatography was carried out using Silica 60 Å (particle size 40−63 µm, Sigma Aldrich, UK) as the stationary phase. Automatic flash column chromatography was carried out using a Buchi Reveleris X2 with Buchi FlashPure Cartridges (12–40 g, particle size 15–50 µm). Analytical TLC was performed on precoated silica gel plates (0.25 mm thick, 60 F254, Merck, Germany) and visualized using both short and long waved ultraviolet light in combination with standard laboratory stains (bromocresol purple, potassium permanganate and ninhydrin). Size exclusion chromatography was performed using Bio-Rad Bio-Beads® S-X1 Beads. High-resolution mass spectrometry was carried out by staff at the Mass Spectrometry Service, Department of Chemistry, University of Manchester. Compounds **1**,[1] **10**·HPF_6,[2] and **S27**[2] were prepared in accordance with literature procedures.

[1] Kennedy, S. H.; Dherange, B. D.; Berger, K. J.; Levin, M. D. *Nature*, **2021**, *593*, 223-227.
[2] Power, M. J.; Morris, D. T. J.; Vitorica-Yrezabal, I.J.; Leigh*,* D. A. *J. Am. Chem. Soc.* **2023**, *145*, 8593-8599.

3. Experimental data

3.1. Synthetic schemes

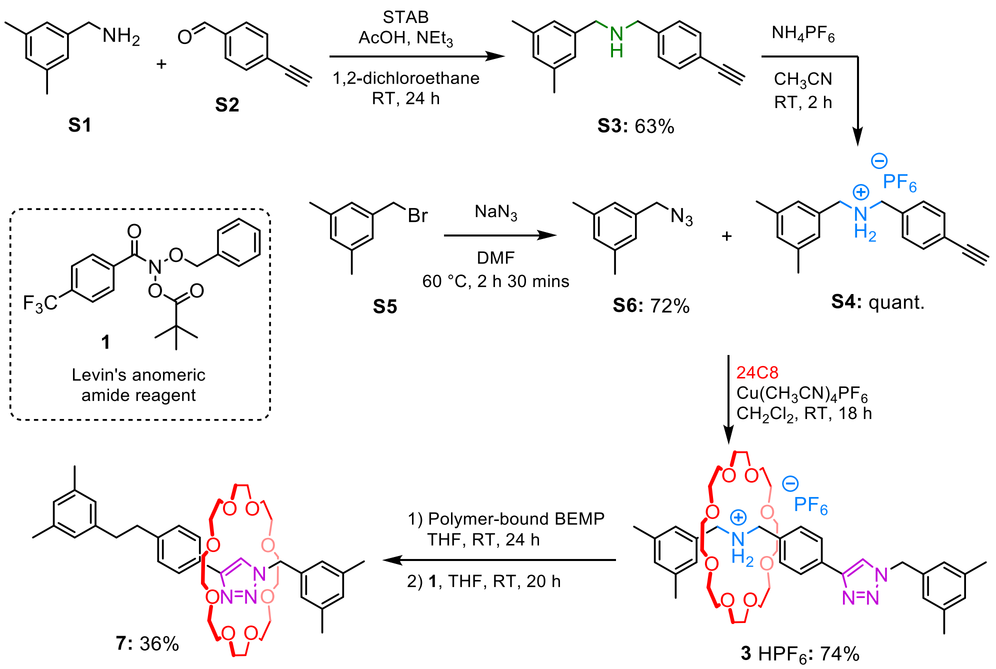

Scheme S1. Synthetic pathway to triazole-containing rotaxane **7**.

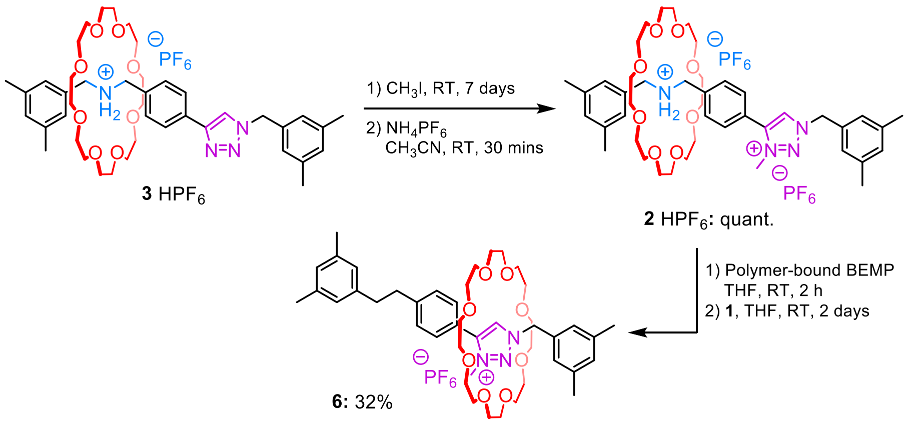

Scheme S2. Synthetic pathway to triazolium-containing rotaxane **6**.

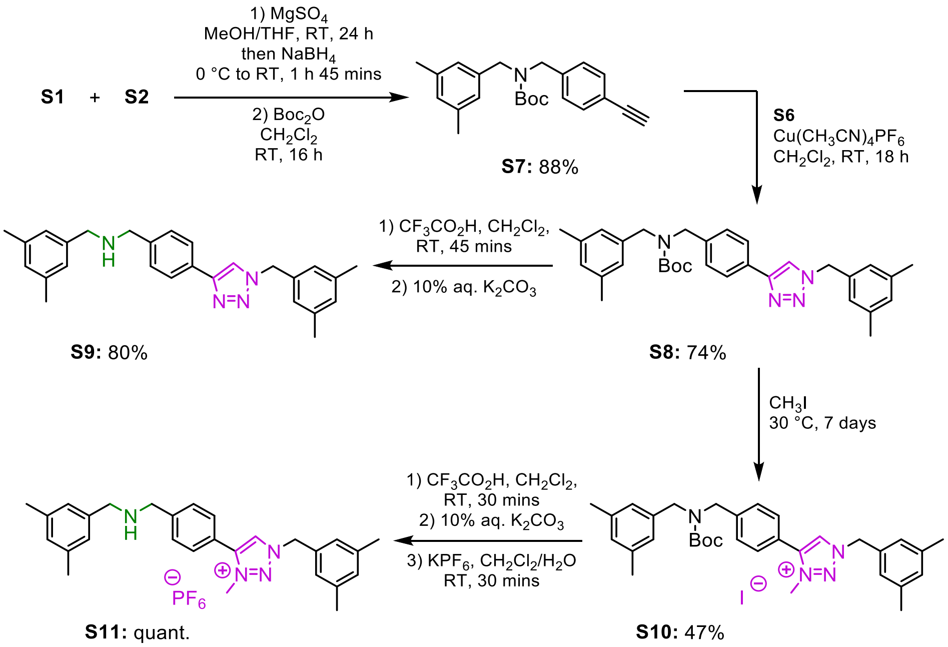

Scheme S3. Synthetic pathway to threads **S9** and **S11**.

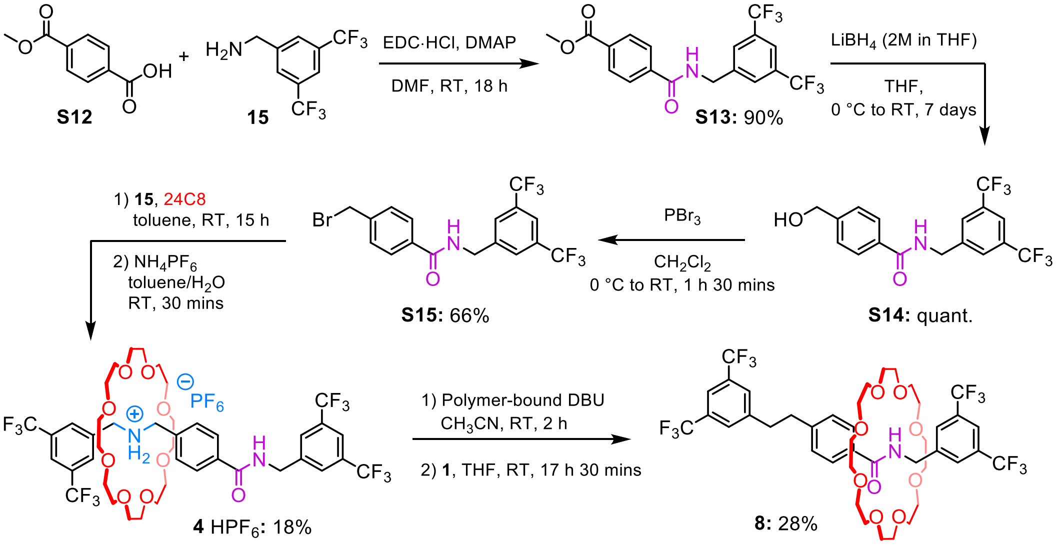

Scheme S4. Synthetic pathway to amide-containing rotaxane **8**.

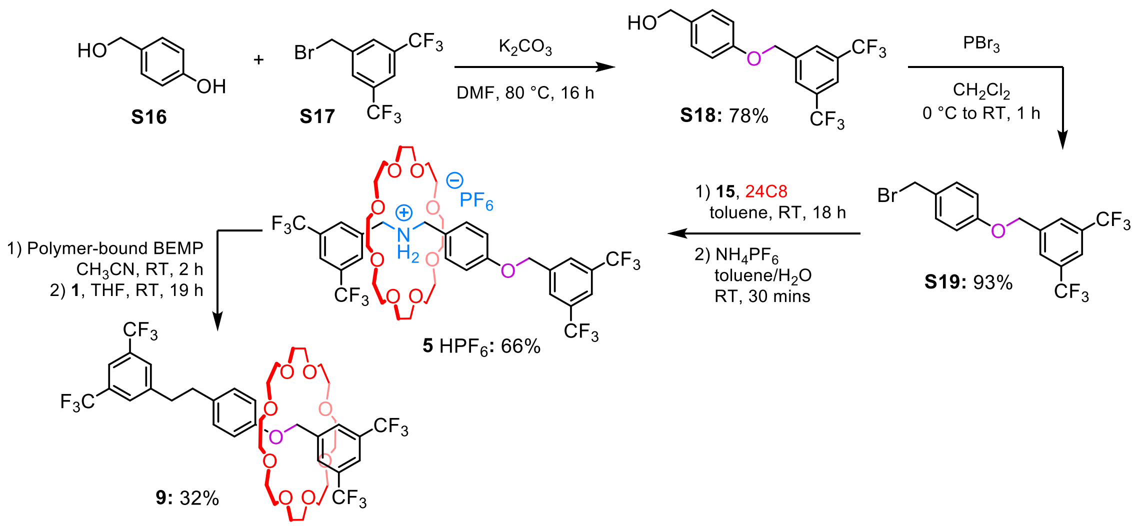

Scheme S5. Synthetic pathway to ether-containing rotaxane **9**.

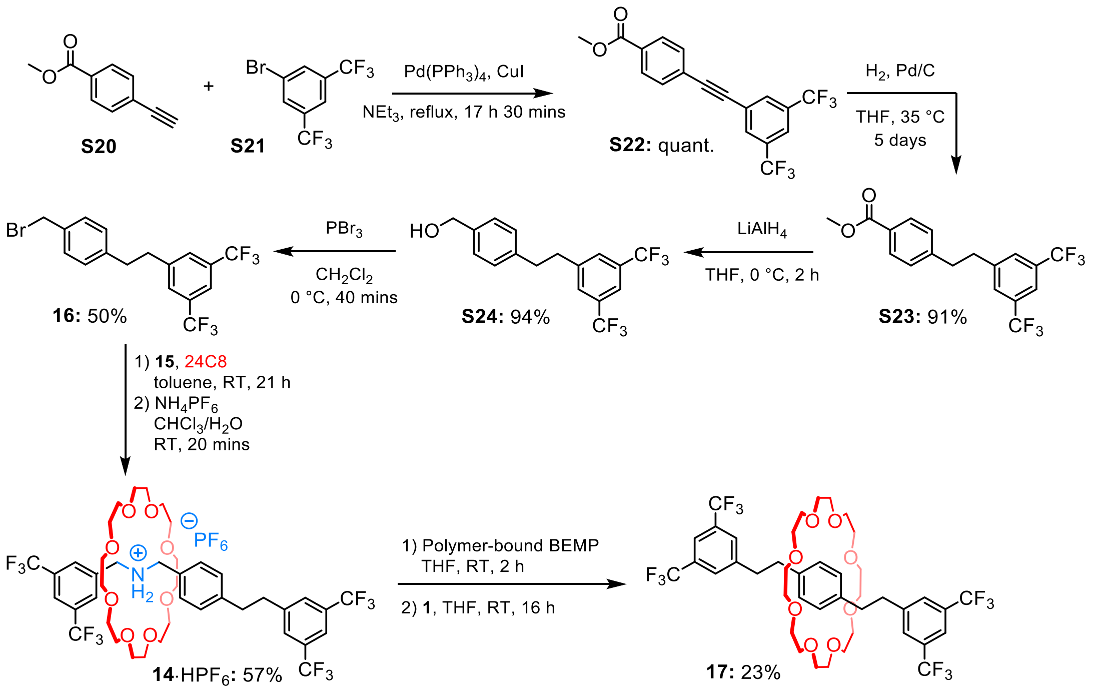

Scheme S6. Synthetic pathway to ‘impossible’ rotaxane **17**.

3.2. Experimental procedures and characterization data

S3

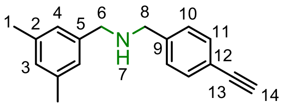

A solution of 3,5-dimethylbenzylamine **S1** (200 mg, 1.48 mmol, 1 eq.) and dry NEt_3 (242 µL, 1.78 mmol, 1.2 eq.) in dry 1,2-dichloroethane (10 mL), was stirred at room temperature, under a nitrogen atmosphere, for 15 minutes. 4-Ethynylbenzaldehyde **S2** (193 mg, 1.48 mmol, 1 eq.) was added to the reaction mixture, followed by STAB (627 mg, 2.96 mmol, 2 eq.) and AcOH (170 µL, 2.96 mmol, 2 eq.). The reaction mixture was stirred at room temperature, under nitrogen atmosphere, for 24 hours. The organic solution was washed with a 1M aqueous solution of NaOH (1 × 10 mL). The aqueous layer was extracted with $CHCl_3$ (2 × 10 mL). The combined organic layers were dried over $MgSO_4$, filtered, and the solvent was removed under reduced pressure. The residue was purified by automated flash column chromatography (SiO_2, CH_2Cl_2/MeOH 100:0 to 98:2) to yield **S3** (245 mg, 63%) as a yellow oil.
^{1}H NMR (600 MHz, $CDCl_3$, 298 K) δ 7.47 (d, ^3J = 8.2 Hz, 2H, H_{10}), 7.31 (d, ^3J = 8.4 Hz, 2H, H_{11}), 6.94 (s, 2H, H_4), 6.90 (s, 1H, H_3), 3.81 (s, 2H, H_8), 3.72 (s, 2H, H_6), 3.06 (s, 1H, H_{14}), 2.31 (s, 6H, H_1).
^{13}C NMR (151 MHz, $CDCl_3$, 298 K) δ 141.47 (C_9), 140.13 (C_5), 138.11 (C_2), 132.31 (C_{10}), 128.78 (C_3), 128.20 (C_{11}), 126.10 (C_4), 120.68 (C_{12}), 83.81 (C_{13}), 77.02 (C_{14}), 53.28 (C_6), 53.06 (C_8), 21.42 (C_1).
HRMS (ESI^+): calculated for $C_{18}H_{20}N$ $[M+H]^+$: 250.1590, found 250.1582.

S4

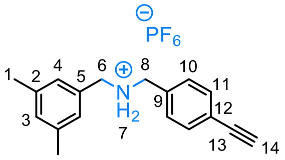

To a solution of **S3** (245 mg, 0.94 mmol, 1 eq.) in CH_3CN (5 mL), was added NH_4PF_6 (764 mg, 4.68 mmol, 5 eq.). The reaction mixture was stirred at room temperature for 2 hours. The solvent was removed under reduced pressure. The residue was dissolved in EtOAc (10 mL) and washed with distilled water (1 × 10 mL). The aqueous layer was extracted with EtOAc (3 × 10 mL). The combined organic layers were dried over $MgSO_4$, filtered, and the solvent was evaporated under reduced pressure, to yield **S4** (383 mg, quant.) as a pale-yellow solid, which was used without any further purification.
^{1}H NMR (600 MHz, CD_3CN, 298 K) δ 7.57 (d, ^3J = 8.4 Hz, 2H, H_{10}), 7.45 (d, ^3J = 8.2 Hz, 2H, H_{11}), 7.11 (s, 1H, H_3), 7.06 (s, 2H, H_4), 4.21 (s, 2H, H_8), 4.14 (s, 2H, H_6), 3.50 (s, 1H, H_{14}), 2.32 (s, 6H, H_1).
^{13}C NMR (151 MHz, CD_3CN, 298 K) δ 139.93 (C_2), 133.48 (C_{10}), 132.16 (C_3), 132.08 (C_9), 131.47 (C_{11}), 131.06 (C_5), 128.74 (C_4), 124.43 (C_{12}), 83.29 (C_{13}), 80.43 (C_{14}), 52.48 (C_6), 51.84 (C_8), 21.24 (C_1).
HRMS (ESI^+): calculated for $C_{18}H_{20}N$ $[M-PF_6]^+$: 250.1590, found 250.1582.

S6

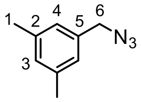

A solution of 3,5-dimethylbenzyl bromide **S5** (2.00 g, 10 mmol, 1 eq.) and NaN_3 (975 mg, 15 mmol, 1.5 eq.) in dry DMF (20 mL), was heated at 60 °C, under a nitrogen atmosphere, for 2 hours 30 minutes. The solvent was removed under reduced pressure. The residue was dissolved in a mixture of Et_2O / $LiCl_{(aq)}$ 5% (20 mL/20 mL). The two layers were separated, and the organic layer was washed again with a 5% aqueous

solution of LiCl (2 × 20 mL). The organic layer was dried over $MgSO_4$, filtered, and the solvent was removed under reduced pressure, to yield **S6** (1.16 g, 72%) as a colorless oil, which was used without any further purification.
^{1}H NMR (600 MHz, $CDCl_3$, 298 K) δ 6.98 (s, 1H, H_3), 6.93 (s, 2H, H_4), 4.26 (s, 2H, H_6), 2.33 (s, 6H, H_1).
^{13}C NMR (151 MHz, $CDCl_3$, 298 K) δ 138.62 (C_2), 135.34 (C_5), 130.09 (C_3), 126.18 (C_4), 54.98 (C_6), 21.41 (C_1).

3·HPF_6

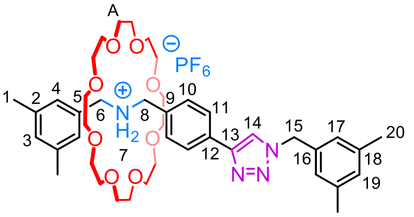

A solution of **S4** (50 mg, 0.123 mmol, 1 eq.) and 24C8 (86 mg, 0.245 mmol, 2 eq.) in dry and degassed CH_2Cl_2 (4 mL), was stirred at room temperature, under a nitrogen atmosphere, for 1 hours 30 minutes. **S6** (25 mg, 0.148 mmol, 1.2 eq.) and $Cu(CH_3CN)_4PF_6$ (48 mg, 0.123 mmol, 1 eq.) were added, and the reaction mixture was stirred at room temperature, under nitrogen atmosphere, for 18 hours. The organic solution was washed with a 10% aqueous solution of EDTA (3 × 5 mL). The combined aqueous layers were extracted with CH_2Cl_2 (1 × 5 mL). The combined organic layers were dried over $MgSO_4$, filtered, and the solvent was removed under reduced pressure. The residue was triturated with Et_2O (6 × 10 mL). The precipitate was purified by automated flash column chromatography (SiO_2, CH_2Cl_2/MeOH 100:0 to 99:1) to yield **3**·HPF_6 (82 mg, 74%) as a colorless solid.
^{1}H NMR (600 MHz, CD_3CN, 298 K) δ 8.13 (s, 1H, H_{14}), 7.90 (d, ^3J = 8.5 Hz, 2H, H_{11}), 7.63 (d, ^3J = 8.4 Hz, 2H, H_{10}), 7.60 – 7.46 (m, 2H, H_7), 7.16 (s, 2H, H_4), 7.07 (s, 1H, H_3), 7.01 (s, 1H, H_{19}), 6.99 (s, 2H, H_{17}), 5.50 (s, 2H, H_{15}), 4.62 – 4.53 (m, 2H, H_8), 4.45 – 4.40 (m, 2H, H_6), 3.44 (s, 32H, H_A), 2.33 (s, 6H, H_1), 2.28 (s, 6H, H_{20}).
^{13}C NMR (151 MHz, CD_3CN, 298 K) δ 147.68 (C_{13}), 139.72 (C_{18}), 139.20 (C_2), 136.65 (C_{16}), 133.10 (C_5), 133.07 & 132.66 (C_9, C_{12}), 132.34 (C_{10}), 131.34 (C_3), 130.84 (C_{19}), 128.95 (C_4), 126.75 (C_{17}), 126.20 (C_{11}), 122.01 (C_{14}), 71.28 (C_A), 54.65 (C_{15}), 53.30 (C_6), 52.87 (C_8), 21.32, 21.23 (C_1, C_{20}).
HRMS (ESI^+) calculated for $C_{43}H_{63}N_4O_8$ $[M-PF_6]^+$: 763.4640, found 763.4631.

7

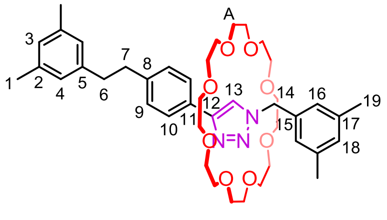

To a solution of **3**·HPF_6 (36 mg, 0.034 mmol, 1 eq.) in dry and degassed THF (1 mL), was added polymer-bound BEMP (34 mg, 0.067 mmol, 2 eq., 2-2.5 mmol/g). The reaction mixture was stirred at room temperature, under nitrogen atmosphere, for 24 hours. The mixture was filtered and the polymer resin was rinsed with dry THF. The filtrate was evaporated under reduced pressure, at room temperature,[3] to give **3** (assumed quant.), which was used directly.

[3] To prevent potential dethreading of the interlocked structure at elevated temperatures due to the similar size of the stoppers and macrocycle cavity, the solution was evaporated at room temperature.

A solution of **1** (26 mg, 0.067 mmol, 2 eq.) in dry and degassed THF (500 µL), was added to the freshly deprotonated rotaxane. The reaction mixture was stirred at room temperature, under a nitrogen atmosphere, for 20 hours. The solvent was removed under reduced pressure at room temperature. The residue was directly purified by automated flash column chromatography (SiO_2, CH_2Cl_2/MeOH 100:0 to 95:5) to yield **7** (9 mg, 36%) as a colorless film.
^{1}H NMR (600 MHz, CD_3CN, 298 K) δ 8.85 (br s, 1H, H_{13}), 7.84 (d, ^3J = 8.0 Hz, 2H, H_{10}), 7.46 (br s, 2H, H_{16}), 7.29 (d, ^3J = 8.2 Hz, 2H, H_9), 6.95 (br s, 1H, H_{18}), 6.90 (s, 2H, H_4), 6.84 (s, 1H, H_3), 5.89 (br s, 2H, H_{14}), 3.35 – 3.22 (m, 32H, H_A), 3.02 – 2.93 (m, 2H, H_7), 2.92 – 2.86 (m, 2H, H_6), 2.31 (s, 6H, H_{19}), 2.26 (s, 6H, H_1).
^{13}C NMR (151 MHz, CD_3CN, 298 K) δ 146.70 (C_{12}), 143.13 (C_8), 142.15 (C_{11}), 138.51 (C_2), 137.85 (C_{17}), 136.52 (C_{15}), 130.95 (C_5), 130.17 (C_{16}), 129.72 (C_9, C_{18}), 128.13 (C_3), 127.37 (C_4), 126.33 (C_{10}), 125.39 (C_{13}), 71.17 (C_A), 54.27 (C_{14}), 37.84, 37.80 (C_6, C_7), 21.35, 21.33 (C_1, C_{19}).
HRMS (ESI$^+$): calculated for $C_{43}H_{61}N_3O_8Na$ $[M+Na]^+$: 770.4351, found 770.4347.

2·HPF_6

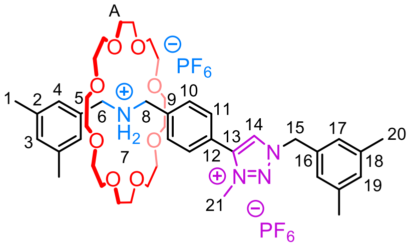

A solution of **3**·HPF_6 (25 mg, 0.028 mmol, 1 eq.) in CH_3I (3 mL) was stirred at room temperature, under nitrogen atmosphere, for 7 days. The solvent was removed under reduced pressure. The residue was dissolved in CH_3CN (2 mL) and NH_4PF_6 (23 mg, 0.14 mmol, 5 eq.) was added. The reaction mixture was stirred at room temperature, for 30 minutes. The solvent was removed under reduced pressure. The residue was partially dissolved in $CHCl_3$ and filtered. The filtrate was evaporated under reduced pressure, to yield **2**·HPF_6 (29 mg, quant.) as a yellow foam, which was used without further purification.
^{1}H NMR (600 MHz, CD_3CN, 298 K) δ 8.45 (s, 1H, H_{14}), 7.81 (d, ^3J = 8.3 Hz, 2H, H_{10}), 7.63 (d, ^3J = 8.3 Hz, 2H, H_{11}), 7.65 – 7.55 (m, 2H, H_7), 7.15 (s, 2H, H_4), 7.12 (s, 3H, H_{17}, H_{19}), 7.09 (s, 1H, H_3), 5.68 (s, 2H, H_{15}), 4.72 – 4.67 (m, 2H, H_8), 4.44 – 4.40 (m, 2H, H_6), 4.15 (s, 3H, H_{21}), 3.49 – 3.39 (m, 32H, H_A), 2.33 (s, 6H, H_1), 2.32 (s, 6H, H_{20}).
^{13}C NMR (151 MHz, CD_3CN, 298 K) δ 144.00 (C_{13}), 140.19, 139.36 (C_2, C_{18}), 137.34 (C_9), 132.91 (C_{10}), 132.78, 132.76 (C_5, C_{16}), 132.12 (C_{19}), 131.52 (C_3), 130.38 (C_{11}), 129.30 (C_{14}), 128.86 (C_4), 127.83 (C_{17}), 123.90 (C_{12}), 71.24 (C_A), 58.22 (C_{15}), 53.57 (C_6), 52.47 (C_8), 39.70 (C_{21}), 21.31, 21.22 (C_1, C_{20}).
HRMS (ESI$^+$): calculated for $C_{44}H_{65}N_4O_8$ $[M-PF_6-HPF_6]^+$: 777.4797, found 777.4790.

6

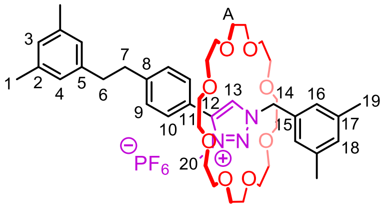

To a solution of **2**·HPF_6 (29 mg, 0.028 mmol, 1 eq.) in dry and degassed THF (1 mL), was added polymer-bound BEMP (28 mg, 0.056 mmol, 2 eq., 2-2.5 mmol/g). The reaction mixture was stirred at room temperature, under a nitrogen atmosphere, for 2 hours. The mixture was filtered and the polymer resin was rinsed with dry THF. The filtrate was evaporated under reduced pressure at room temperature,[3] to give **2** (assumed quant.), which was used directly.

A solution of **1** (22 mg, 0.056 mmol, 2 eq.) in dry and degassed THF (500 µL), was added to the freshly deprotonated rotaxane. The reaction mixture was stirred at room temperature, under a nitrogen atmosphere, for 2 days. The solvent was removed under reduced pressure at room temperature. The residue was directly purified by automated flash column chromatography (SiO_2, CH_2Cl_2/MeOH 100:0 to 99:1) to yield **6** (8 mg, 32%) as a colorless film.
^{1}H NMR (600 MHz, CD_3CN, 298 K) δ 9.26 (s, 1H, H_{13}), 7.59 (d, ^3J = 8.3 Hz, 2H, H_9), 7.43 (s, 2H, H_{16}), 7.41 (d, ^3J = 8.3 Hz, 2H, H_{10}), 7.04 (s, 1H, H_{18}), 6.84 (s, 2H, H_4), 6.83 (s, 1H, H_3), 6.07 (s, 2H, H_{14}), 4.30 (s, 3H, H_{20}), 3.36 – 3.26 (m, 32H, H_A), 2.99 (t, ^3J = 7.9 Hz, 2H, H_7), 2.87 (t, ^3J = 7.8 Hz, 2H, H_6), 2.32 (s, 6H, H_{19}), 2.23 (s, 6H, H_1).
^{13}C NMR (151 MHz, CD_3CN, 298 K) δ 146.36 (C_8), 142.26 (C_5), 141.78 (C_{12}), 138.71 (C_2), 138.48 (C_{17}), 133.07 (C_{15}), 132.42 (C_{13}), 131.11 (C_{18}), 130.88 (C_{16}), 130.27, 130.25 (C_9, C_{10}), 128.40 (C_3), 127.25 (C_4), 122.43 (C_{11}), 71.61 (C_A), 57.60 (C_{14}), 39.24 (C_{20}), 37.97, 37.74 (C_6, C_7), 21.50, 21.33 (C_1, C_{19}).
HRMS (ESI$^+$): calculated for $C_{44}H_{64}N_3O_8$ $[M\text{-}PF_6]^+$: 762.4688, found 762.4687.

S7

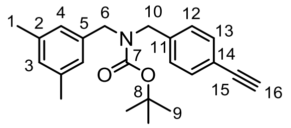

A solution of 3,5-dimethylbenzylamine **S1** (1.05 mL, 7.39 mmol, 1 eq.), 4-ethynylbenzaldehyde **S2** (1.15 g, 8.87 mmol, 1.2 eq.) and anhydrous $MgSO_4$ (4.7 g, 39.05 mmol, 5.3 eq.) in MeOH (70 mL) and dry THF (9 mL) was stirred overnight under a nitrogen atmosphere. The reaction mixture was cooled to 0 °C and $NaBH_4$ (1.18 g, 31.11 mmol, 4.2 eq.) was added portion-wise over 0.5 h. The reaction was stirred for 50 minutes at 0 °C, followed by 25 minutes at room temperature, before being quenched with distilled water (6 mL). The solid particles were filtered off through celite and the cake was washed with CH_2Cl_2 (100 mL). The organic phase was dried over $MgSO_4$, filtered, and the solvent was removed under reduced pressure. The crude residue was dissolved in dry CH_2Cl_2 (60 mL), under a nitrogen atmosphere, and Boc anhydride (1.840 g, 8.43 mmol, 1.14 eq.) was added and stirred at room temperature for 16 hours. The reaction mixture was evaporated onto celite under reduced pressure and purified by automated flash column chromatography (SiO_2, PE: EtOAc 100:0 to 90:10) to yield **S7** (2.269 g, 88%) as a colourless oil which upon standing solidified to a colourless solid.
Note*: The molecule exists as a mixture of rotamers at room temperature, due to restricted rotation around the C-N bonds.
^{1}H NMR (600 MHz, $CDCl_3$, 298 K) δ 7.45 (d, ^3J = 7.7 Hz, 2H, H_{13}), 7.22 – 7.09 (m, 2H, H_{12}), 6.90 (s, 1H, H_3), 6.86 – 6.73 (m, 2H, H_4), 4.45 – 4.20 (m, 4H, H_{6+10}), 3.07 (s, 1H, H_{16}), 2.29 (s, 6H, H_1), 1.51 – 1.45 (m, 9H, H_9).
^{13}C NMR (151 MHz, $CDCl_3$, 298 K) δ 156.18 (C_7), 139.27 (C_{11}), 139.15 ($C_{11'}$), 138.24 (C_2), 137.80 (C_5), 137.67 ($C_{5'}$), 132.41 (C_{13}), 129.10 (C_3), 129.00 ($C_{3'}$), 128.00 (C_{12}), 127.34 ($C_{12'}$), 125.98 (C4), 125.35 ($C_{4'}$), 121.02 (C_{14}), 85.33 (C_8), 83.65 (C_{15}), 77.22 (C_{16}), 49.58 – 48.87 (C_{6+10}), 28.58 – 27.57 (C_9), 21.44 (C_1).
HRMS (ESI$^+$): calculated for $C_{23}H_{27}NO_2Na$ $[M+Na]^+$: 372.1939, found 372.1945.

S8

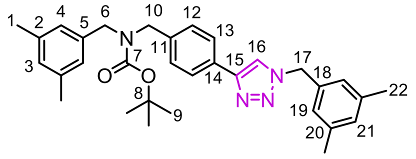

A solution of **S7** (486 mg, 1.39 mmol, 1 eq.), **S6** (225 mg, 1.40 mmol, 1 eq.) and $Cu(CH_3CN)_4PF_6$ (532.4 mg, 1.43 mmol 1 eq.) in dry and degassed CH_2Cl_2 (20 mL) was stirred at room temperature, under a nitrogen atmosphere, for 18 hours. The reaction mixture was diluted with CH_2Cl_2 (100 mL) and washed with a 10% aqueous solution of EDTA (2 × 80 mL). The organic layer was dried over $MgSO_4$, filtered, and the solvent

was removed under reduced pressure. The crude was purified by automated flash column chromatography (SiO_2, hexane/EtOAc 100:0 to 70:30) to yield **S8** (522 mg, 74%) as a colourless oil.
Note*: The molecule exists as a mixture of rotamers at room temperature, due to restricted rotation around the C-N bonds.
^{1}H NMR (600 MHz, $CDCl_3$, 298 K) δ 7.77 (d, ^3J = 7.8 Hz, 2H, H_{13}), 7.65 (s, 1H, H_{16}), 7.29 – 7.19 (m, 2H, H_{12}), 7.00 (s, 1H, H_{21}), 6.93 (s, 2H, H_{19}), 6.90 (s, 1H, H_3), 6.86 – 6.77 (m, 2H, H_4), 5.49 (s, 2H, H_{17}), 4.45 – 4.24 (m, 4H, H_{6+10}), 2.31 (s, 6H, H_{22}), 2.29 (s, 6H, H_1), 1.50 (s, 9H, H_9).
^{13}C NMR (151 MHz, $CDCl_3$, 298 K) δ 156.22 (C_7), 148.07 (C_{15}), 139.02 (C_{20}), 138.37 (C_{11}), 138.20 (C_2), 137.93 (C_5), 137.81 ($C_{5'}$) 134.64 (C_{18}), 130.53 (C_{21}), 129.73 (C_{14}), 129.06 (C_3), 128.94 ($C_{3'}$), 128.63 (C_{12}) 127.90 ($C_{12'}$), 126.02 (C_{19}), 125.95 (C_{13+4}), 125.36 ($C_{4'}$), 119.41 (C_{16}), 80.21 (C_8), 54.38 (C_{17}), 49.34 – 49.09 (m, C_{6+10}), 48.80 ($C_{10'}$), 28.59 (C_9), 21.44 ($C_{1/22}$), 21.36 ($C_{1/22}$).
HRMS (ESI^+): calculated for $C_{32}H_{38}N_4O_2Na$ $[M+Na]^+$: 533.2892, found 533.2903.

S9

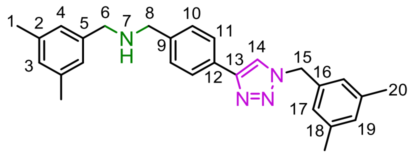

S8 (25 mg, 49 µmol, 1 eq.) was dissolved in CF_3CO_2H/CH_2Cl_2 (0.25 mL, 1:4 *v/v*) and the reaction mixture was stirred at room temperature, under a nitrogen atmosphere, for 45 minutes. The solution was quenched with a 10% aqueous solution of K_2CO_3 (6 mL). The organic layer was separated, dried over $MgSO_4$, filtered, and the solvent was removed under reduced pressure to yield **S9** (16 mg, 80%) as a colourless solid, which was used without further purification.
^{1}H NMR (600 MHz, CD_3CN, 298 K) δ 8.06 (s, 1H, H_{14}), 7.77 (d, ^3J = 8.1 Hz, 2H, H_{11}), 7.39 (d, ^3J = 7.7 Hz, 2H, H_{10}), 6.99 (s, 1H, H_{19}), 6.97 (s, 2H, H_{17}), 6.95 (s, 2H, H_4), 6.88 (s, 1H, H_3), 5.48 (s, 2H, H_{15}), 3.75 (s, 2H, H_8), 3.66 (s, 2H, H_6), 2.27 (s, 12H, H_{1+20}).
^{13}C NMR (151 MHz, CD_3CN, 298 K) δ 148.30 (C_{13}), 141.92 (C_9), 141.78 (C_5), 139.68 ($C_{2/18}$), 138.71 ($C_{2/18}$), 136.74 (C_{16}), 130.78 (C_{19}), 130.48 (C_{12}), 129.57 (C_{10}), 129.13 (C_3), 126.86 ($C_{4/17}$), 126.70 ($C_{4/17}$), 126.30 (C_{11}), 121.52 (C_{14}), 54.56 (C_{15}), 53.61 (C_6), 53.36 (C_8), 21.33 ($C_{1/20}$), 21.24 ($C_{1/20}$).
HRMS (ESI^+): calculated for $C_{27}H_{31}N_4$ $[M+H]^+$: 411.2549, found 411.2546.

S10

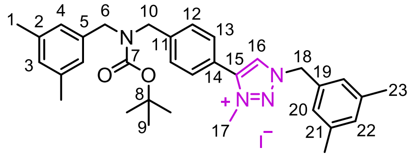

S8 (98 mg, 0.192 mmol, 1 eq.) was dissolved in iodomethane (5 mL), heated to 30 °C, and stirred under a nitrogen atmosphere for 7 days. The reaction mixture was evaporated onto celite under reduced pressure and purified by automated flash column chromatography (SiO_2, CH_2Cl_2/MeOH 100:0 to 90:10) to yield **S10** (59 mg, 47%) as a yellow oil.
Note*: The molecule exists as a mixture of rotamers at room temperature, due to restricted rotation around the C-N bonds.
^{1}H NMR (600 MHz, CD_3CN, 298 K) δ 8.70 (s, 1H, H_{16}), 7.58 (d, ^3J = 8.1 Hz, 2H, H_{13}), 7.45 (d, ^3J = 7.7 Hz, 2H, H_{12}), 7.16 (s, 2H, H_{20}), 7.11 (s, 1H, H_{22}), 6.91 (s, 1H, H_3), 6.84 (s, 2H, H_4), 5.73 (s, 2H, H_{18}), 4.50 – 4.34 (m, 4H, H_{6+10}), 4.18 (s, 3H, H_{17}), 2.32 (s, 6H, H_{23}), 2.26 (s, 6H, H_1), 1.44 (s, 9H, H_9).
^{13}C NMR (151 MHz, CD_3CN, 298 K) δ 156.61 (C_7), 144.17 (C_{15}), 144.03 – 143.84 (m, C_{14}), 140.04 (C_{21}), 139.07 (C_5), 139.02 (C_2), 132.89 (C_{19}), 131.99 (C_{22}), 130.44 (C_{13}), 129.57 (C_3), 129.39 (C_{12}), 129.29 (C_{16}), 127.80 (C_{20}), 126.32 (C_4), 121.95 (C_{11}), 80.77 (C_8), 58.01 (C_{18}), 51.39 – 50.30 (m, C_{6+10}), 39.71 (C_{17}), 28.52 (C_9), 21.31 ($C_{1/23}$), 21.20 ($C_{1/23}$).

HRMS (ESI^+): calculated for $C_{33}H_{41}N_4O_2^+$ $[M]^+$: 525.3230, found 525.3228.

S11

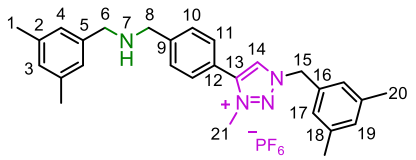

S10 (30 mg, 46 µmol, 1 eq.) was dissolved in CF_3CO_2H/CH_2Cl_2 (0.45 mL, 1:8 *v/v*) and the reaction mixture was stirred at room temperature, under a nitrogen atmosphere, for 30 minutes. The solution was quenched with a 10% aqueous solution of K_2CO_3 (4 mL). The organic layer was separated, dried over $MgSO_4$, filtered, and the solvent was removed under reduced pressure. The residue was dissolved in a mixture of CH_2Cl_2/H_2O (15 mL, 2:1 *v/v*) and KPF_6 (103 mg, 0.56 mmol, 12.2 eq.) was added. The biphasic solution was stirred vigorously at room temperature for 30 minutes. The aqueous layer was extracted with CH_2Cl_2 (3 × 10 mL). The combined organic layers were dried over $MgSO_4$, filtered, and the solvent removed under reduced pressure to yield **S11** (26 mg, 46 µmol, quant.) as a yellow solid.
^{1}H NMR (600 MHz, CD_3CN, 298 K) δ 8.43 (s, 1H, H_{14}), 7.61 (d, ^3J = 7.9 Hz, 2H, H_{10}), 7.55 (d, ^3J = 8.0 Hz, 2H, H_{11}), 7.12 (s, 3H, H_{17+19}), 6.97 (s, 2H, H_4), 6.91 (s, 1H, H_3), 5.66 (s, 2H, H_{15}), 4.18 (s, 3H, H_{21}), 3.87 (s, 2H, H_8), 3.72 (s, 2H, H_6), 2.32 (s, 6H, H_{20}), 2.28 (s, 6H, H_1).
^{13}C NMR (151 MHz, CD_3CN, 298 K) δ 145.78 (C_{12}), 144.55 (C_{13}), 140.95 (C_5), 140.18 (C_{18}), 138.86 (C_2), 132.86 (C_{16}), 132.09 (C_{19}), 130.29 ($C_{10/11}$), 130.15 ($C_{10/11}$), 129.42 (C_3), 129.08 (C_{14}), 127.80 (C_{17}), 127.00 (C_4), 121.76 (C_9), 58.15 (C_{15}), 53.63 (C_6), 52.97 (C_8), 39.58 (C_{21}), 21.35 ($C_{1/20}$), 21.24 ($C_{1/20}$).
HRMS (ESI^+): calculated for $C_{28}H_{33}N_4^+$ $[M]^+$: 425.2705, found 425.2689.

S13

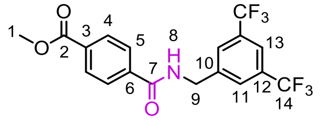

To a solution of 4-(methoxycarbonyl)benzoic acid **S12** (241 mg, 1.34 mmol, 1 eq.) and 3,5-bis(trifluoromethyl)benzylamine **15** (325 mg, 1.34 mmol, 1 eq.) in dry DMF (10 mL), was added DMAP (326 mg, 2.67 mmol, 1 eq.) and EDC·HCl (384 mg, 2.00 mmol, 1.5 eq.). The reaction mixture was stirred at room temperature, under a nitrogen atmosphere, for 18 h. The solvent was removed under reduced pressure. The residue was dissolved in $CHCl_3$ (10 mL) and washed with a 1M aqueous solution of HCl (3 × 10 mL). The combined aqueous layers were extracted with $CHCl_3$ (1 × 10 mL). The combined organic layers were washed with a saturated aqueous solution of $NaHCO_3$ (1 × 10 mL). The aqueous layer was extracted with $CHCl_3$ (1 × 10 mL). The combined organic layers were dried over $MgSO_4$, filtered, and the solvent was removed under reduced pressure. The residue was purified by automated flash column chromatography (SiO_2, CH_2Cl_2/MeOH 100:0 to 99:1) to yield **S13** (488 mg, 90%) as a colorless solid.
^{1}H NMR (600 MHz, CD_3CN, 298 K) δ 8.07 (d, ^3J = 8.7 Hz, 2H, H_4), 7.95 (s, 2H, H_{11}), 7.92 (d, ^3J = 8.6 Hz, 2H, H_5), 7.91 (s, 1H, H_{13}), 7.81 (br t, 1H, ^3J = 5.7 Hz, H_8), 4.68 (d, ^3J = 6.0 Hz, 2H, H_9), 3.89 (s, 3H, H_1).
^{13}C NMR (151 MHz, CD_3CN, 298 K) δ 167.37 (C_7), 167.05 (C_2), 143.49 (C_{10}), 139.01 (C_3), 133.85 (C_6), 131.96 (q, $^2J_{C\text{-}F}$ = 33.0 Hz, C_{12}), 130.41 (C_4), 129.22 (q, $^3J_{C\text{-}F}$ = 3.9 Hz, C_{11}), 128.39 (C_5), 124.57 (q, $^1J_{C\text{-}F}$ = 271.9 Hz, C_{14}), 122.01 (sept, $^3J_{C\text{-}F}$ = 4.0 Hz, C_{13}), 52.98 (C_1), 43.47 (C_9).
HRMS (ESI^+): calculated for $C_{18}H_{13}F_6NO_3Na$ $[M+Na]^+$: 428.0692, found 428.0692.

S14

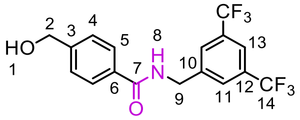

To a 2M solution of $LiBH_4$ in THF (2.58 mL, 5.16 mmol, 5 eq.) at 0 °C, was added dropwise a solution of **S13** (418 mg, 1.03 mmol, 1 eq.) in dry THF (10 mL). The reaction mixture was warmed to room temperature, and stirred, under a nitrogen atmosphere, for 7 days. The reaction was quenched by dropwise addition of a saturated aqueous solution of NH_4Cl (10 mL). The residual THF was removed under reduced pressure and the resulting aqueous solution was extracted with $CHCl_3$ (3 × 10 mL). The combined organic layers were dried over $MgSO_4$, filtered, and the solvent was removed under reduced pressure, to yield **S14** (388 mg, quant.) as a colorless oil, which was used without further purification.

^{1}H NMR (600 MHz, CD_3CN, 298 K) δ 7.93 (s, 2H, H_{11}), 7.90 (s, 1H, H_{13}), 7.80 (d, ^3J = 8.3 Hz, 2H, H_4), 7.72 (br t, 1H, H_8), 7.42 (d, ^3J = 8.8 Hz, 2H, H_5), 4.66 (d, ^3J = 6.1 Hz, 2H, H_9), 4.63 (d, ^3J = 5.8 Hz, 2H, H_2), 3.39 (t, ^3J = 5.9 Hz, 1H, H_1).

^{13}C NMR (151 MHz, CD_3CN, 298 K) δ 168.08 (C_7), 147.01 (C_3), 143.86 (C_{10}), 133.65 (C_6), 131.92 (q, $^2J_{C-F}$ = 33.0 Hz, C_{12}), 129.10 (C_{11}), 128.13 (C_4), 127.42 (C_5), 124.58 (q, $^1J_{C-F}$ = 271.9 Hz, C_{14}), 121.90 (sept, $^3J_{C-F}$ = 3.9 Hz, C_{13}), 64.12 (C_2), 43.34 (C_9).

HRMS (ESI^+): calculated for $C_{17}H_{13}F_6NO_2Na$ $[M+Na]^+$: 400.0743, found 400.0742.

S15

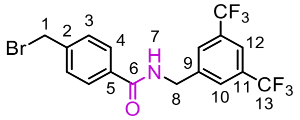

To a solution of **S14** (220 mg, 0.58 mmol, 1 eq.) in dry CH_2Cl_2 (5 mL) at 0 °C, was added dropwise PBr_3 (83 µL, 0.87 mmol, 1.5 eq.). The reaction mixture was allowed to warm to room temperature, and stirred under a nitrogen atmosphere, for 1 hour 30 minutes. The reaction mixture was cooled at 0 °C and was quenched by dropwise addition of distilled water (5 mL). The two layers were separated, and the aqueous layer was extracted with $CHCl_3$ (3 × 5 mL). The combined organic layers were washed with a saturated aqueous solution of NH_4Cl (1 × 5 mL). The aqueous layer was extracted with $CHCl_3$ (1 × 5 mL). The combined organic layers were dried over $MgSO_4$, filtered, and the solvent was removed under reduced pressure. The residue was purified by automated flash column chromatography (SiO_2, CH_2Cl_2) to yield **S15** (168 mg, 66%) as a colorless solid.

^{1}H NMR (600 MHz, CD_3CN, 298 K) δ 7.94 (s, 2H, H_{10}), 7.90 (s, 1H, H_{12}), 7.81 (d, ^3J = 8.4 Hz, 2H, H_3), 7.70 (br t, 1H, ^3J = 5.8 Hz, H_7), 7.52 (d, ^3J = 7.9 Hz, 2H, H_4), 4.66 (d, ^3J = 6.0 Hz, 2H, H_8), 4.62 (s, 2H, H_1).

^{13}C NMR (151 MHz, CD_3CN, 298 K) δ 167.58 (C_6), 143.72 (C_9), 142.87 (C_2), 134.95 (C_5), 131.94 (q, $^2J_{C-F}$ = 33.0 Hz, C_{11}), 130.20 (C_4), 129.13 (C_{10}), 128.65 (C_3), 124.58 (q, $^1J_{C-F}$ = 271.9 Hz, C_{13}), 121.93 (sept, $^3J_{C-F}$ = 4.0 Hz, C_{12}), 43.38 (C_8), 33.59 (C_1).

HRMS (ESI^+): calculated for $C_{17}H_{12}F_6BrNONa$ $[M+Na]^+$: 461.9899, found 461.9897.

4·HPF_6

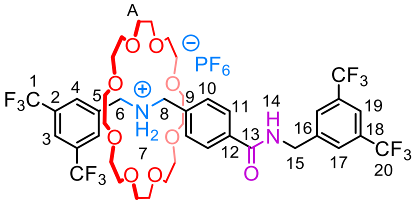

A solution of 3,5-bis(trifluoromethyl)benzylamine **15** (179 mg, 0.74 mmol, 2 eq.), 24C8 (261 mg, 0.74 mmol, 2 eq.) and **S15** (162 mg, 0.37 mmol, 1 eq.) in dry toluene (5 mL), was stirred at room temperature, under nitrogen atmosphere, for 15 hours. A solution of NH_4PF_6 (603 mg, 3.70 mmol, 10 eq.) in distilled water (5 mL) was added, and the biphasic solution was vigorously stirred at room temperature, for 30 minutes. The two layers were separated, and the organic layer was washed with distilled water (3 × 5 mL). The combined aqueous layers were extracted with toluene (1 × 5 mL). The combined organic layers were dried over $MgSO_4$, filtered, and the solvent was removed under reduced pressure. The residue was triturated with Et_2O (2 × 10 mL). The precipitate was purified by automated flash column chromatography (SiO_2, CH_2Cl_2/MeOH 100:0 to 99:1) to yield **4**·HPF_6 (75 mg, 18%) as a colorless film.
^{1}H NMR (600 MHz, CD_3CN, 298 K) δ 8.26 (s, 2H, H_4), 8.08 (s, 1H, H_3), 7.95 (s, 2H, H_{17}), 7.92 (d, ^3J = 8.1 Hz, 2H, H_{10}), 7.91 (s, 1H, H_{19}), 7.85 – 7.77 (m, 2H, H_7), 7.76 (br t, 1H, H_{14}), 7.63 (d, ^3J = 8.0 Hz, 2H, H_{11}), 4.84 – 4.78 (m, 2H, H_6), 4.68 (d, ^3J = 6.0 Hz, 2H, H_{15}), 4.58 – 4.47 (m, 2H, H_8), 3.49 – 3.42 (m, 16H, H_A), 3.39 – 3.33 (m, 16H, $H_{A'}$).
^{13}C NMR (151 MHz, CD_3CN, 298 K) δ 167.48 (C_{13}), 143.60 (C_{16}), 136.17 & 136.13 (C_5, C_{12}), 135.77 (C_9), 133.17 (C_4), 132.34 – 131.42 (m, C_2, C_{18}), 131.50 (C_{11}), 129.24 (q, $^3J_{C-F}$ = 4.1 Hz, C_{17}), 128.64 (C_{10}), 124.58 & 124.44 (2q, $^1J_{C-F}$ = 271.8 Hz, C_1, C_{20}), 124.01 (sept, $^3J_{C-F}$ = 4.0 Hz, C_3), 122.02 (sept, $^3J_{C-F}$ = 4.0 Hz, C_{19}), 71.12 (C_A), 53.28 (C_8), 51.97 (C_6), 43.44 (C_{15}).
HRMS (ESI$^+$): calculated for $C_{42}H_{51}F_{12}N_2O_9$ $[M-PF_6]^+$: 955.3397, found 955.3390.

8

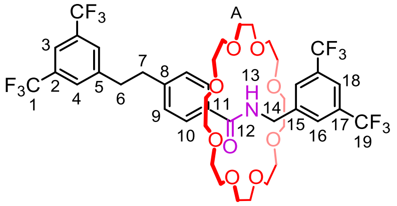

To a solution of **4**·HPF_6 (75 mg, 0.068 mmol, 1 eq.) in dry CH_3CN (5 mL), was added polymer-bound DBU (273 mg, 0.406 mmol, 6 eq., 1.5-2.5 mmol/g). The reaction mixture was stirred at room temperature, under a nitrogen atmosphere, for 2 hours. The mixture was filtered and the polymer resin was rinsed with dry CH_3CN. The filtrate was evaporated under reduced pressure at room temperature,[3] to give **4** (assumed quant.), which was used directly without further purification.
A solution of **1** (54 mg, 0.136 mmol, 2 eq.) in dry and degassed THF (3 mL), was added to the freshly deprotonated rotaxane. The reaction mixture was stirred at room temperature, under nitrogen atmosphere, for 17 hours 30 minutes. The solvent was removed under reduced pressure at room temperature. The residue was directly purified by automated flash column chromatography (SiO_2, CH_2Cl_2/MeOH 100:0 to 99:1) to yield **8** (18 mg, 28%) as a colorless film.
^{1}H NMR (600 MHz, CD_3CN, 298 K) δ 8.69 (s, 2H, H_{16}), 7.88 (d, ^3J = 8.3 Hz, 2H, H_9), 7.82 (s, 1H, H_{18}), 7.80 (s, 1H, H_3), 7.77 (s, 2H, H_4), 7.75 (br t, 1H, H_{13}), 7.19 (d, ^3J = 8.3 Hz, 2H, H_{10}), 4.78 (d, ^3J = 3.8 Hz, 2H, H_{14}), 3.35 – 3.27 (m, 16H, H_A), 3.15 – 3.07 (m, 18H, H_6, $H_{A'}$), 3.01 (t, ^3J = 7.7 Hz, 2H, H_7).
^{13}C NMR (151 MHz, CD_3CN, 298 K) δ 167.07 (C_{12}), 145.68 (C_5), 144.38 (C_8), 142.37 (C_{15}), 136.55 (q, $^3J_{C-F}$ = 3.8 Hz, C_{16}), 134.55 (C_{11}), 131.69 (q, $^2J_{C-F}$ = 32.9 Hz, C_2), 130.29 (q, $^3J_{C-F}$ = 3.9 Hz, C_4), 129.53 (q,

$^{2}J_{C\text{-}F}$ = 32.4 Hz, C_{17}), 128.97 & 128.93 (C_9, C_{10}), 125.32 & 124.66 (2q, $^{1}J_{C\text{-}F}$ = 271.8 Hz, C_1, C_{19}), 121.10 – 120.50 (m, C_3, C_8), 71.17 (C_A), 45.30 (C_{14}), 37.21 & 37.05 (C_6, C_7).
HRMS (ESI^+): calculated for calculated for $C_{42}H_{49}F_{12}NO_9Na$ $[M+Na]^+$: 962.3108, found 962.3097.

S18

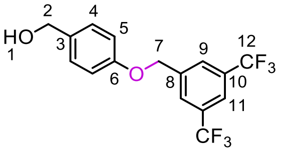

To a solution of 4-hydroxybenzyl alcohol **S16** (146 mg, 1.18 mmol, 1.2 eq.) in dry DMF (15 mL), was added K_2CO_3 (542 mg, 3.92 mmol, 4 eq.). The reaction mixture was stirred at room temperature, under nitrogen atmosphere, for 15 minutes. 3,5-Bis(trifluoromethyl)benzyl bromide **S17** (179 µL, 0.98 mmol, 1 eq.) was added dropwise, and the reaction mixture was heated at 80 °C, under nitrogen atmosphere, for 16 hours. The solvent was removed under reduced pressure. The residue was dissolved in $CHCl_3$ (10 mL) and washed with distilled water (1 × 10 mL). The aqueous layer was extracted with $CHCl_3$ (2 × 10 mL). The combined organic layers were dried over $MgSO_4$, filtered, and the solvent was removed under reduced pressure. The residue was purified by automated flash column chromatography (SiO_2, Hexane/EtOAc 100/0 to 50/50) to yield **S18** (269 mg, 78%) as a pale-yellow solid.
^{1}H NMR (600 MHz, CD_3CN, 298 K) δ 8.05 (s, 2H, H_9), 7.97 (s, 1H, H_{11}), 7.29 (d, ^{3}J = 8.8 Hz, 2H, H_4), 6.99 (d, ^{3}J = 8.7 Hz, 2H, H_5), 5.23 (s, 2H, H_7), 4.50 (d, ^{3}J = 5.9 Hz, 2H, H_2), 3.09 (t, ^{3}J = 5.9 Hz, 1H, H_1).
^{13}C NMR (151 MHz, CD_3CN, 298 K) δ 158.30 (C_6), 141.79 (C_8), 136.14 (C_3), 132.09 (q, $^{2}J_{C\text{-}F}$ = 33.2 Hz, C_{10}), 129.37 (C_4), 128.80 (q, $^{3}J_{C\text{-}F}$ = 3.9 Hz, C_9), 124.54 (q, $^{1}J_{C\text{-}F}$ = 271.8 Hz, C_{12}), 122.67 (sept, $^{3}J_{C\text{-}F}$ = 3.9 Hz, C_{11}), 115.59 (C_5), 69.02 (C_7), 64.28 (C_2).
HRMS ($APCI^+$): calculated for $C_{16}H_{11}F_6O$ $[M\text{-}OH]^+$: 333.0709, found 333.0699.

S19

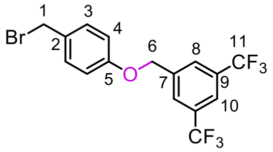

To a solution of **S18** (258 mg, 0.74 mmol, 1 eq.) in dry CH_2Cl_2 (5 mL) at 0 °C, was added dropwise PBr_3 (105 µL, 1.10 mmol, 1.5 eq.). The reaction mixture was allowed to warm to room temperature, and stirred under nitrogen atmosphere, for 1 hour. The reaction mixture was cooled at 0 °C and was quenched by dropwise addition of distilled water (5 mL). The two layers were separated, and the aqueous layer was extracted with $CHCl_3$ (3 × 5 mL). The combined organic layers were washed with a saturated aqueous solution of NH_4Cl (1 × 5 mL). The aqueous layer was extracted with $CHCl_3$ (1 × 5 mL). The combined organic layers were dried over $MgSO_4$, filtered, and the solvent was removed under reduced pressure, to yield **S19** (285 mg, 93%) as a colorless solid, which was used without further purification.
^{1}H NMR (600 MHz, CD_3CN, 298 K) δ 8.05 (s, 2H, H_8), 7.98 (s, 1H, H_{10}), 7.39 (d, ^{3}J = 8.7 Hz, 2H, H_3), 7.01 (d, ^{3}J = 8.7 Hz, 2H, H_4), 5.24 (s, 2H, H_6), 4.59 (s, 2H, H_1).
^{13}C NMR (151 MHz, CD_3CN, 298 K) δ 159.21 (C_5), 141.45 (C_7), 132.26 (C_2), 132.12 (q, $^{2}J_{C\text{-}F}$ = 33.2 Hz, C_9), 131.70 (C_3), 128.87 (q, $^{3}J_{C\text{-}F}$ = 3.9 Hz, C_8), 124.52 (q, $^{1}J_{C\text{-}F}$ = 271.9 Hz, C_{11}), 122.81 (sept, $^{3}J_{C\text{-}F}$ = 3.9 Hz, C_{10}), 118.33 (C_4), 69.07 (C_6), 34.91 (C_1).
HRMS ($APCI^+$): calculated for $C_{16}H_{11}F_6O$ $[M\text{-}Br]^+$: 333.0709, found 333.0699.

5·HPF$_6$

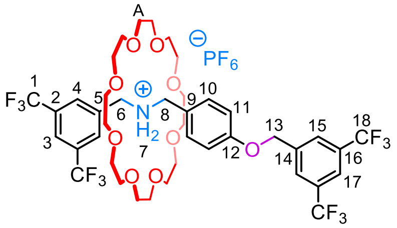

A solution of 3,5-bis(trifluoromethyl)benzylamine **15** (294 mg, 1.21 mmol, 2 eq.), 24C8 (426 mg, 1.21 mmol, 2 eq.) and **S19** (250 mg, 0.61 mmol, 1 eq.) in dry toluene (5 mL), was stirred at room temperature, under a nitrogen atmosphere, for 18 hours. A solution of NH_4PF_6 (986 mg, 6.05 mmol, 10 eq.) in distilled water (5 mL) was added, and the biphasic solution was vigorously stirred at room temperature, for 30 minutes. The two layers were separated, and the organic layer was washed with distilled water (3 × 5 mL). The combined aqueous layers were extracted with EtOAc (2 × 5 mL). The combined organic layers were dried over $MgSO_4$, filtered, and the solvent was removed under reduced pressure. The residue was purified by automated flash column chromatography (SiO_2, CH_2Cl_2/MeOH 100:0 to 99:1) to yield **5**·HPF$_6$ (430 mg, 66%) as a colorless solid.

^{1}H NMR (600 MHz, CD_3CN, 298 K) δ 8.25 (s, 2H, H_4), 8.06 (s, 1H, H_3), 8.04 (s, 2H, H_{15}), 7.97 (s, 1H, H_{17}), 7.70 – 7.58 (m, 2H, H_7), 7.46 (d, ^3J = 8.7 Hz, 2H, H_{10}), 7.09 (d, ^3J = 8.6 Hz, 2H, H_{11}), 5.29 (s, 2H, H_{13}), 4.78 – 4.73 (m, 2H, H_6), 4.38 – 4.33 (m, 2H, H_8), 3.46 – 3.41 (m, 16H, H_A), 3.37 – 3.31 (m, 16H, $H_{A'}$).

^{13}C NMR (151 MHz, CD_3CN, 298 K) δ 159.91 (C_{12}), 141.39 (C_{14}), 136.35 (C_5), 133.15 (q, $^3J_{C\text{-}F}$ = 3.9 Hz, C_4), 133.10 (C_{10}), 132.14 (q, $^2J_{C\text{-}F}$ = 33.6 Hz, C_2/C_{16}), 131.70 (q, $^2J_{C\text{-}F}$ = 33.6 Hz, C_2/C_{16}), 128.83 (q, $^3J_{C\text{-}F}$ = 33.6 Hz, C_{15}), 125.22 (C_9), 124.50 (q, $^1J_{C\text{-}F}$ = 271.8 Hz, C_1/C_{18}), 124.47 (q, $^1J_{C\text{-}F}$ = 271.8 Hz, C_1/C_{18}), 123.84 (sept, $^3J_{C\text{-}F}$ = 4.1 Hz, C_3), 122.82 (sept, $^3J_{C\text{-}F}$ = 3.9 Hz, C_{17}), 116.21 (C_{11}), 71.11 (C_A), 69.00 (C_{13}), 53.27 (C_8), 51.58 (C_6).

HRMS (ESI$^+$): calculated for $C_{41}H_{50}F_{12}NO_9$ $[M\text{-}PF_6]^+$: 928.3288, found 928.3284.

9

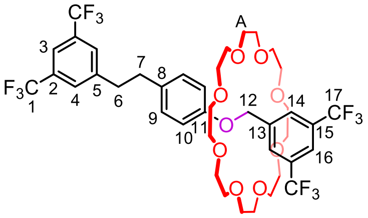

To a solution of **5**·HPF$_6$ (209 mg, 0.195 mmol, 1 eq.) in dry CH_3CN (5 mL), was added polymer-bound BEMP (487 mg, 0.974 mmol, 5 eq., 2-2.5 mmol/g). The reaction mixture was stirred at room temperature, under nitrogen atmosphere, for 2 hours. The mixture was filtered and the polymer resin was rinsed with dry CH_3CN. The filtrate was evaporated under reduced pressure, at room temperature,[3] to give **5** (assumed quant.) which was used directly.

A solution of **1** (154 mg, 0.390 mmol, 2 eq.) in dry and degassed THF (10 mL), was added to the freshly deprotonated rotaxane. The reaction mixture was stirred at room temperature, under nitrogen atmosphere, for 19 hours. The solvent was removed under reduced pressure at room temperature. The residue was directly purified by automated flash column chromatography (SiO_2, CH_2Cl_2/MeOH 100:0 to 99:1) to yield **9** (57 mg, 32%) as a colorless film.

^{1}H NMR (600 MHz, CD_3CN, 298 K) δ 8.45 (s, 2H, H_{14}), 8.01 (s, 2H, H_4), 7.90 (s, 1H, H_{16}), 7.78 (s, 1H, H_3), 7.21 (d, ^3J = 8.4 Hz, 2H, H_9), 7.09 (d, ^3J = 8.6 Hz, 2H, H_{10}), 5.45 (s, 2H, H_{12}), 3.39 – 3.27 (m, 32H, H_A), 3.23 – 3.13 (m, 2H, H_6), 3.11 – 2.99 (m, 2H, H_7).

^{13}C NMR (151 MHz, CD_3CN, 298 K) δ 158.40 (C_{11}), 147.10 (C_5), 142.92 (C_{13}), 134.81 (C_8), 131.82 – 130.29 (m, C_2, C_4, C_{14}, C_{15}), 130.23 (C_9), 124.97 (q, $^1J_{C\text{-}F}$ = 271.9 Hz, C_1), 124.97 (q, $^1J_{C\text{-}F}$ = 271.9 Hz, C_{17}),

121.66 (sept, $^{3}J_{C\text{-}F}$ = 4.1 Hz, C_{16}), 120.09 (sept, $^{3}J_{C\text{-}F}$ = 4.1 Hz, C_3), 115.98 (C_{10}), 71.32 (C_A), 69.03 (C_{12}), 37.46 (C_6), 35.80 (C_7).
HRMS ($APCI^+$): calculated for $C_{41}H_{48}F_{12}O_9Na$ $[M+Na]^+$: 935.2999, found 935.2981.

S22

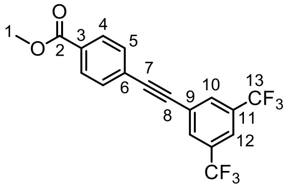

A solution of methyl-4-ethynylbenzoate **S20** (615 mg, 3.84 mmol, 1.4 eq.), 1,3-bis(trifluoromethyl)-5-bromobenzene **S21** (473 µL, 2.74 mmol, 1 eq.) and CuI (51 mg, 0.27 mmol, 0.1 eq.) in NEt_3 (20 mL) was sparged with nitrogen gas for 15 minutes, after which $Pd(PPh_3)_4$ (158 mg, 0.13 mmol, 0.05 eq.) was added. The reaction mixture was refluxed, under nitrogen atmosphere, for 17 hours 30 minutes. The NEt_3 was removed under reduced pressure and the residue was dissolved in $CHCl_3$ (20 mL). The organic solution was washed with a 1M aqueous solution of HCl (2 × 20 mL). The combined aqueous layers were extracted with $CHCl_3$ (1 × 20 mL). The combined organic layers were drier over $MgSO_4$, filtered, and the solvent was removed under reduced pressure. The residue was purified by automated flash column chromatography (SiO_2, CH_2Cl_2) to yield **S22** (1.02 g, quant.) as a yellow solid.
^{1}H NMR (600 MHz, CD_3CN, 298 K) δ 8.13 (s, 2H, H_{10}), 8.04 (d, ^{3}J = 8.5 Hz, 2H, H_4), 8.02 (s, 1H, H_{12}), 7.69 (d, ^{3}J = 8.5 Hz, 2H, H_5), 3.89 (s, 3H, H_1).
^{13}C NMR (151 MHz, CD_3CN, 298 K) δ 166.98 (C_2), 132.90 (q, $^{3}J_{C\text{-}F}$ = 3.8 Hz, C_{10}), 132.82 (C_4), 132.63 (q, $^{2}J_{C\text{-}F}$ = 33.6 Hz, C_{11}), 131.69 (C_6), 130.53 (C_5), 127.28 (C_3), 125.99 (C_9), 124.14 (q, $^{1}J_{C\text{-}F}$ = 272.3 Hz, C_{13}), 123.39 (sept, $^{3}J_{C\text{-}F}$ = 3.9 Hz, C_{12}), 92.19 (C_7), 89.53 (C_8), 52.97 (C_1).
HRMS ($APCI^+$): calculated for $C_{18}H_{11}F_6O_2$ $[M+H]^+$: 373.0658, found 373.0649.

S23

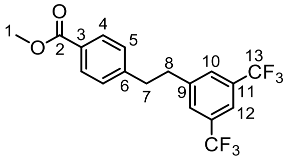

To a solution of **S22** (1.02 g, 2.74 mmol, 1 eq.) in dry and degassed THF (15 mL), was added Pd/C (500 mg, 50% w/w). The reaction mixture was stirred at 35 °C, with hydrogen gas bubbling (balloon), for 5 days. The mixture was filtered through a celite pad and rinsed with THF. The filtrate was evaporated under reduced pressure, to yield **S23** (936 mg, 91%) as a yellow solid, which was used without further purification.
^{1}H NMR (600 MHz, CD_3CN, 298 K) δ 7.90 (d, ^{3}J = 8.3 Hz, 2H, H_4), 7.82 (s, 1H, H_{12}), 7.76 (s, 2H, H_{10}), 7.30 (d, ^{3}J = 8.4 Hz, 2H, H_5), 3.85 (s, 3H, H_1), 3.13 – 3.06 (m, 2H, H_8), 3.05 – 3.01 (m, 2H, H_7).
^{13}C NMR (151 MHz, CD_3CN, 298 K) δ 167.61 (C_2), 147.50 (C_3), 145.52 (C_9), 131.79 (q, $^{2}J_{C\text{-}F}$ = 32.8 Hz, C_{11}), 130.40 (C_4), 130.28 (q, $^{3}J_{C\text{-}F}$ = 4.1 Hz, C_{10}), 129.80 (C_5), 129.26 (C_6), 124.67 (q, $^{1}J_{C\text{-}F}$ = 271.8 Hz, C_{13}), 120.92 (sept, $^{3}J_{C\text{-}F}$ = 4.0 Hz, C_{12}), 52.59 (C_1), 37.56 (C_7), 37.25 (C_8).

S24

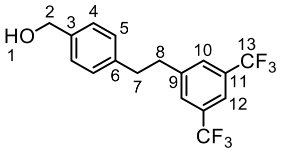

To a suspension of $LiAlH_4$ (112 mg, 2.94 mmol, 1.2 eq.) in dry THF (10 mL) at 0°C, was added dropwise a solution of **S23** (922 mg, 2.45 mmol, 1 eq.) in dry THF (10 mL). The reaction mixture was stirred at 0 °C, under nitrogen atmosphere, for 2 hours. 10 mL of saturated aqueous solution of NH_4Cl was added dropwise at 0 °C. The THF was removed under reduced pressure and the resulting aqueous layer was extracted with Et_2O (3 × 10 mL). The combined organic layers were dried over $MgSO_4$, filtered, and the solvent was removed under reduced pressure, to yield **S24** (806 mg, 94%) as a pale-yellow solid which was used without further purification.
^{1}H NMR (600 MHz, CD_3CN, 298 K) δ 7.82 (s, 1H, H_{12}), 7.78 (s, 2H, H_{10}), 7.24 (d, ^3J = 8.0 Hz, 2H, H_4), 7.17 (d, ^3J = 8.1 Hz, 2H, H_5), 4.52 (s, 2H, H_2), 3.10 – 3.04 (m, 2H, H_8), 2.98 – 2.92 (m, 2H, H_7).
^{13}C NMR (151 MHz, CD_3CN, 298 K) δ 145.99 (C_9), 141.02 (C_3), 140.62 (C_6), 131.75 (q, $^2J_{C\text{-}F}$ = 32.8 Hz, C_{11}), 130.25 (q, $^3J_{C\text{-}F}$ = 3.8 Hz, C_{10}), 129.40 (C_4), 127.84 (C_5), 124.71 (q, $^1J_{C\text{-}F}$ = 271.7 Hz, C_{13}), 120.79 (sept, $^3J_{C\text{-}F}$ = 3.9 Hz, C_{12}), 64.51 (C_2), 37.75 (C_7), 37.32 (C_8).
HRMS ($APCI^+$): calculated for $C_{17}H_{13}F_6$ $[M\text{-}OH]^+$: 331.0916, found 331.0906.

16

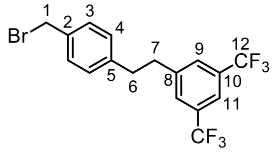

To a solution of **S24** (798 mg, 2.29 mmol, 1 eq.) in dry CH_2Cl_2 (15 mL) at 0 °C, was added dropwise PBr_3 (327 µL, 3.44 mmol, 1.5 eq.). The reaction mixture was allowed to warm to room temperature, and stirred under nitrogen atmosphere, for 40 minutes. The reaction mixture was cooled at 0 °C and was quenched by dropwise addition of distilled water (15 mL). The two layers were separated, and the aqueous layer was extracted with $CHCl_3$ (3 × 15 mL). The combined organic layers were washed with a saturated aqueous solution of NH_4Cl (1 × 15 mL). The aqueous layer was extracted with $CHCl_3$ (1 × 15 mL). The combined organic layers were dried over $MgSO_4$, filtered, and the solvent was removed under reduced pressure. The residue was purified by automated flash chromatography (SiO_2, Hexane/EtOAc 100:0 to 95:5) to yield **12** (470 mg, 50%) as a yellow solid.
^{1}H NMR (600 MHz, CD_3CN, 298 K) δ 7.82 (s, 1H, H_{11}), 7.77 (s, 2H, H_9), 7.34 (d, ^3J = 8.1 Hz, 2H, H_3), 7.19 (d, ^3J = 8.1 Hz, 2H, H_4), 4.57 (s, 2H, H_1), 3.10 – 3.04 (m, 2H, H_7), 2.99 – 2.93 (m, 2H, H_6).
^{13}C NMR (151 MHz, CD_3CN, 298 K) δ 145.78 (C_8), 142.36 (C_5), 137.24 (C_2), 131.77 (q, $^2J_{C\text{-}F}$ = 32.9 Hz, C_{10}), 130.25 (q, $^3J_{C\text{-}F}$ = 4.0 Hz, C_9), 130.19 (C_4), 129.97 (C_5), 124.68 (q, $^1J_{C\text{-}F}$ = 271.7 Hz, C_{12}), 120.85 (sept, $^3J_{C\text{-}F}$ = 4.0 Hz, C_{11}), 37.52 (C_6), 37.31 (C_7), 34.70 (C_1).
HRMS ($APCI^+$): calculated for $C_{17}H_{13}F_6$ $[M\text{-}Br]^+$: 331.0916, found 331.0907.

14·HPF_6

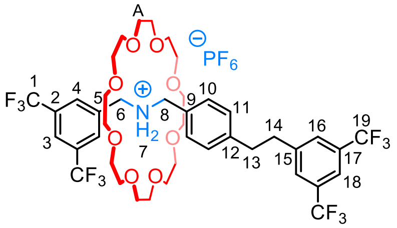

A solution of 3,5-bis(trifluoromethyl)benzylamine **15** (294 mg, 1.21 mmol, 2 eq.), 24C8 (426 mg, 1.21 mmol, 2 eq.) and **16** (249 mg, 0.61 mmol, 1 eq.) in dry toluene (5 mL), was stirred at room temperature, under nitrogen atmosphere, for 21 hours. The solvent was removed under reduced pressure and the residue was dissolved in $CHCl_3$ (5 mL). A solution of NH_4PF_6 (986 mg, 6.05 mmol, 10 eq.) in distilled water (5 mL) was added, and the biphasic solution was vigorously stirred at room temperature, for 20 minutes. The two layers were separated, and the aqueous layer was extracted with $CHCl_3$ (3 × 5 mL). The combined organic layers were dried over $MgSO_4$, filtered, and the solvent was removed under reduced pressure. The residue was purified by automated flash column chromatography (SiO_2, CH_2Cl_2/Acetone 100:0 to 97:3) to yield **10**·HPF_6 (370 mg, 57%) as a colorless solid.
^{1}H NMR (600 MHz, CD_3CN, 298 K) δ 8.25 (s, 2H, H_4), 8.06 (s, 1H, H_3), 7.83 (s, 2H, H_{16}), 7.82 (s, 1H, H_{18}), 7.74 – 7.60 (m, 2H, H_7), 7.42 (d, ^3J = 8.1 Hz, 2H, H_{10}), 7.32 (d, ^3J = 8.1 Hz, 2H, H_{11}), 4.80 – 4.74 (m, 2H, H_6), 4.40 – 4.35 (m, 2H, H_8), 3.48 – 3.39 (m, 16H, H_A), 3.37 – 3.30 (m, 16H, $H_{A'}$), 3.14 – 3.08 (m, 2H, H_{14}), 3.07 – 3.02 (m, 2H, H_{13}).
^{13}C NMR (151 MHz, CD_3CN, 298 K) δ 145.63 (C_{15}), 143.72 (C_{12}), 136.35 (C_5), 133.21 (q, $^3J_{C\text{-}F}$ = 3.8 Hz, C_4), 131.82 (q, $^2J_{C\text{-}F}$ = 32.8 Hz, C_2), 131.72 (q, $^2J_{C\text{-}F}$ = 32.8 Hz, C_{17}), 131.43 (C_9, C_{10}), 130.23 (q, $^3J_{C\text{-}F}$ = 3.9 Hz, C_{16}), 129.99 (C_{11}), 124.68 (q, $^1J_{C\text{-}F}$ = 271.9 Hz, C_1), 124.49 (q, $^1J_{C\text{-}F}$ = 271.9 Hz, C_{19}) 123.88 (sept, $^3J_{C\text{-}F}$ = 4.0 Hz, C_3), 120.92 (sept, $^3J_{C\text{-}F}$ = 4.1 Hz, C_{18}), 71.14 (C_A), 53.59 (C_8), 51.76 (C_6), 37.22 (C_{14}), 36.65 (C_{13}).
HRMS (ESI$^+$): calculated for $C_{42}H_{52}F_{12}NO_8$ $[M\text{-}PF_6]^+$: 926.3501, found 926.3498.

17

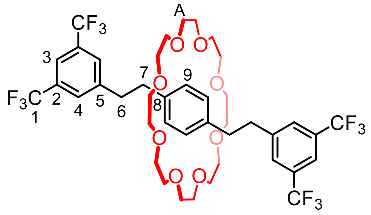

To a solution of **14**·HPF_6 (360 mg, 0.34 mmol, 1 eq.) in dry CH_3CN (10 mL), was added a 2-2.5 mmol/g polymer-bound BEMP (840 mg, 1.68 mmol, 5 eq.). The reaction mixture was stirred at room temperature, under nitrogen atmosphere, for 2 h. The mixture was filtered and the polymer resin was rinsed with dry CH_3CN. The filtrate was evaporated under reduced pressure at room temperature,[5] to give **14** (assumed quant.) which was used directly without further purification.
A solution of **1** (269 mg, 0.68 mmol, 2 eq.) in dry and degassed THF (5 mL), was added to the freshly deprotonated rotaxane. The reaction mixture was stirred at room temperature, under nitrogen atmosphere, for 16 hours. The solvent was removed under reduced pressure at room temperature. The residue was directly purified by automated flash column chromatography (SiO_2, CH_2Cl_2), followed by size exclusion chromatography (Bio-Beads S-X1, $CHCl_3$) to yield **17** (70 mg, 23%) as a colorless solid.
^{1}H NMR (600 MHz, CD_3CN, 298 K) δ 8.16 (s, 4H, H_9), 7.75 (s, 2H, H_3), 7.26 (s, 4H, H_4), 3.33 (s, 32H, H_A), 3.26 – 3.19 (m, 4H, H_7), 3.14 – 3.09 (m, 4H, H_6).
^{13}C NMR (151 MHz, CD_3CN, 298 K) δ 147.76 (C_8), 141.17 (C_5), 131.81 (C_9), 130.74 (q, $^2J_{C\text{-}F}$ = 33.0 Hz, C_2), 129.46 (C_4), 125.15 (q, $^1J_{C\text{-}F}$ = 271.8 Hz, C_1), 119.69 (sept, $^3J_{C\text{-}F}$ = 3.5 Hz, C_3), 71.63 (C_A), 37.13 (C_7), 35.79 (C_6).

4. ¹H NMR comparison of protonated, deprotonated, and skeletally edited rotaxanes

4.1. ¹H NMR comparison of rotaxanes **2**·HPF₆, **2**, **6** and thread **S11**

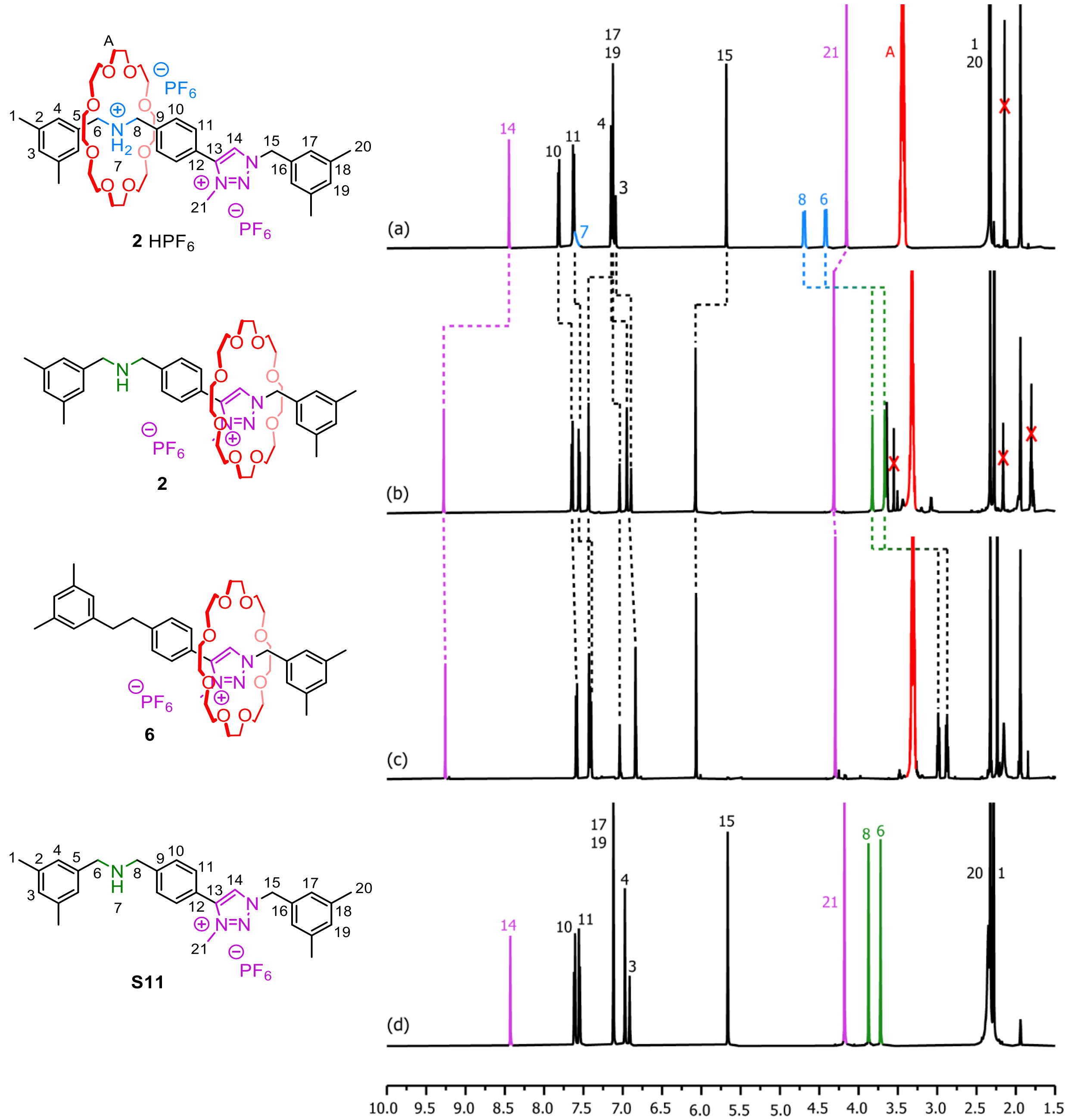

Figure S1. ¹H NMR spectra (CD_3CN, 600 MHz, 298 K) of: (a) protonated [2]rotaxane **2**·HPF_6, (b) deprotonated [2]rotaxane **2**, (c) [2]rotaxane **6** (after nitrogen deletion), (d) thread **S11**.

4.2. ^{1}H NMR comparison of rotaxanes **3**·HPF_6, **3**, **7** and thread **S9**

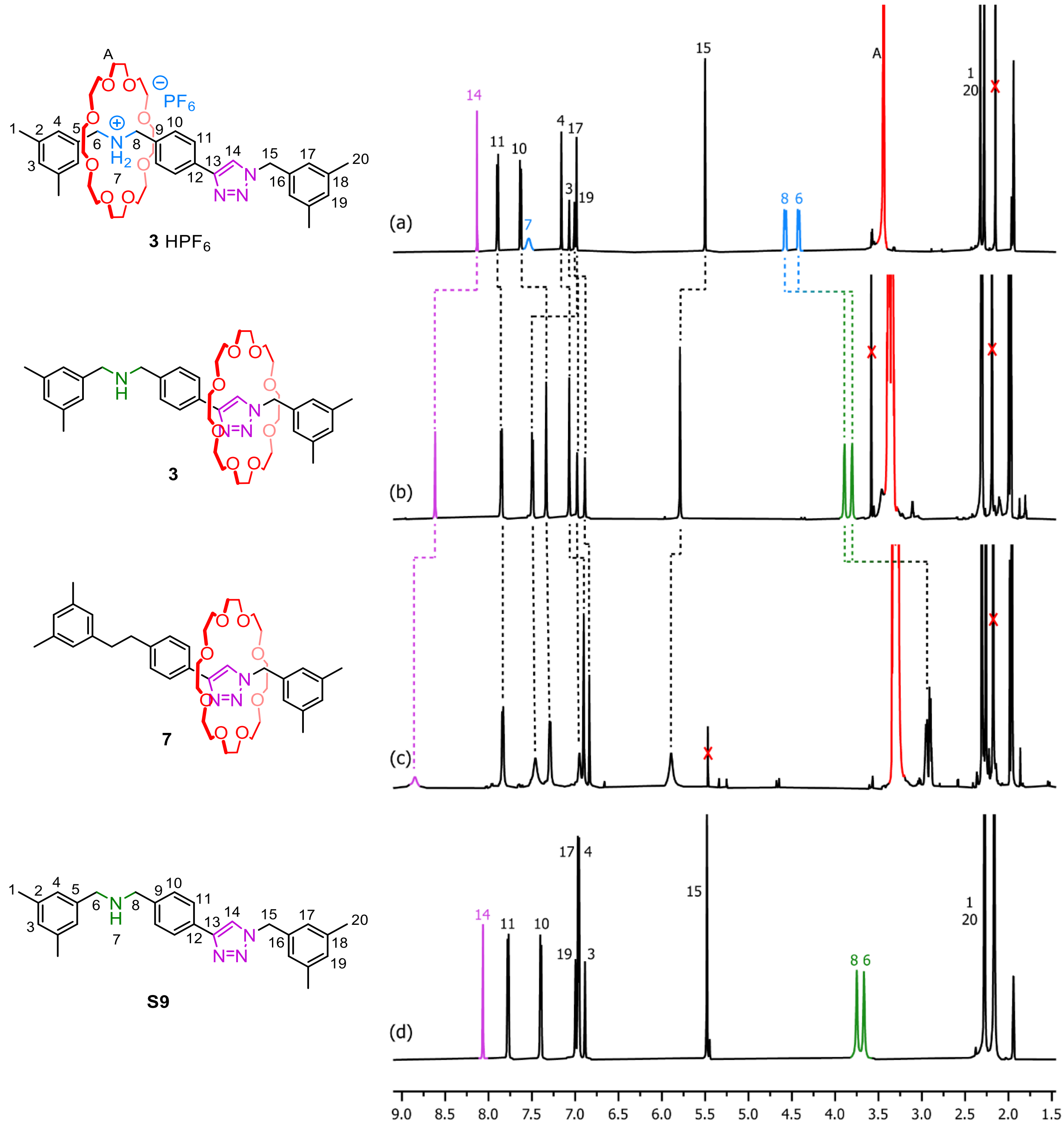

Figure S2. ^{1}H NMR spectra (CD_3CN, 600 MHz, 298 K) of: (a) protonated [2]rotaxane **3**·HPF_6, (b) deprotonated [2]rotaxane **3**, (c) [2]rotaxane **7** (after nitrogen deletion), (d) thread **S9**.

4.3. ^{1}H NMR comparison of rotaxanes **4**·HPF$_6$, **4** and **8**

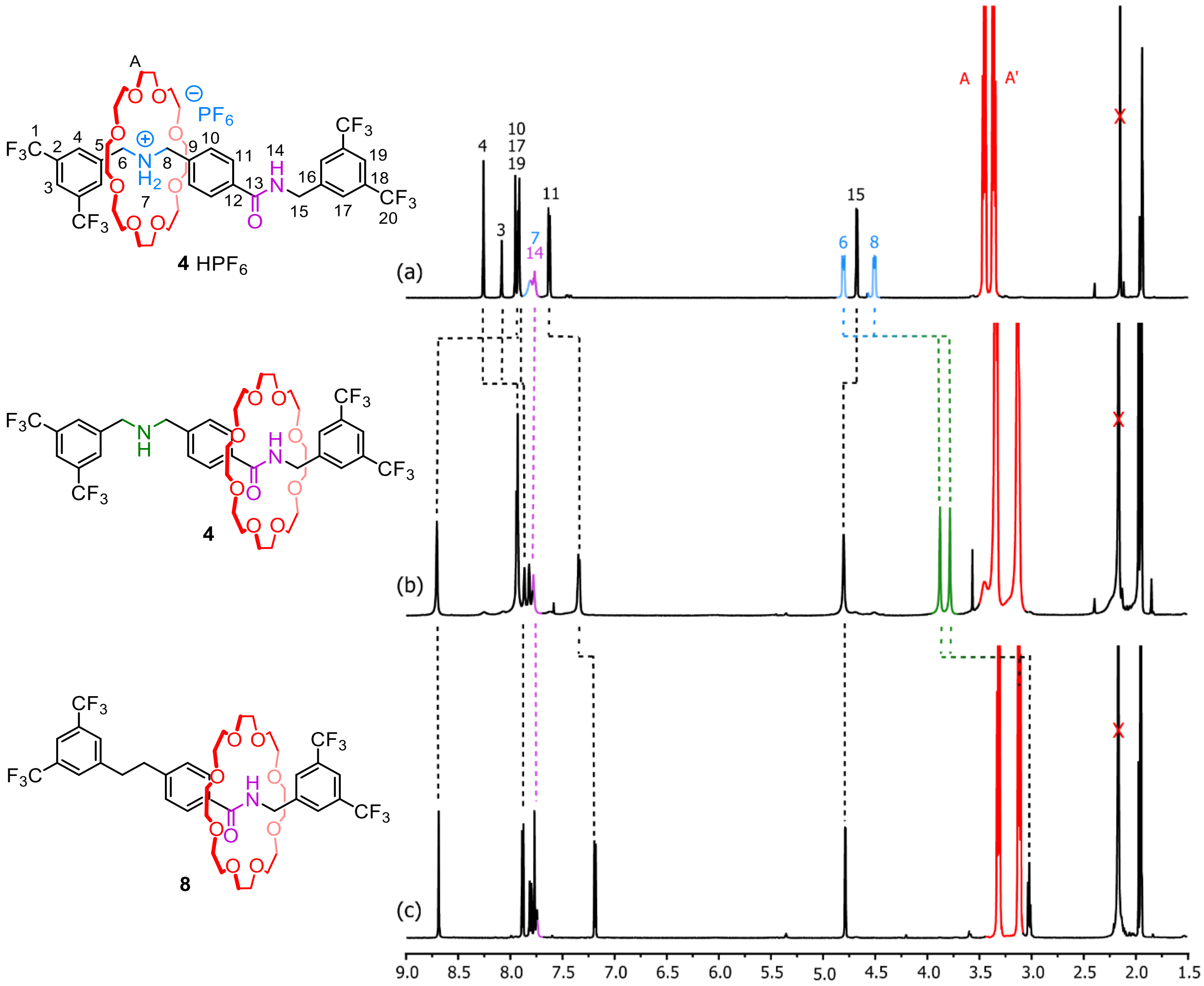

Figure S3. ^{1}H NMR spectra (CD_3CN, 600 MHz, 298 K) of: (a) protonated [2]rotaxane **4**·HPF$_6$, (b) deprotonated [2]rotaxane **4**, (c) [2]rotaxane **8** (after nitrogen deletion).

4.4. ^{1}H NMR comparison of rotaxanes **5**·HPF$_6$, **5** and **9**

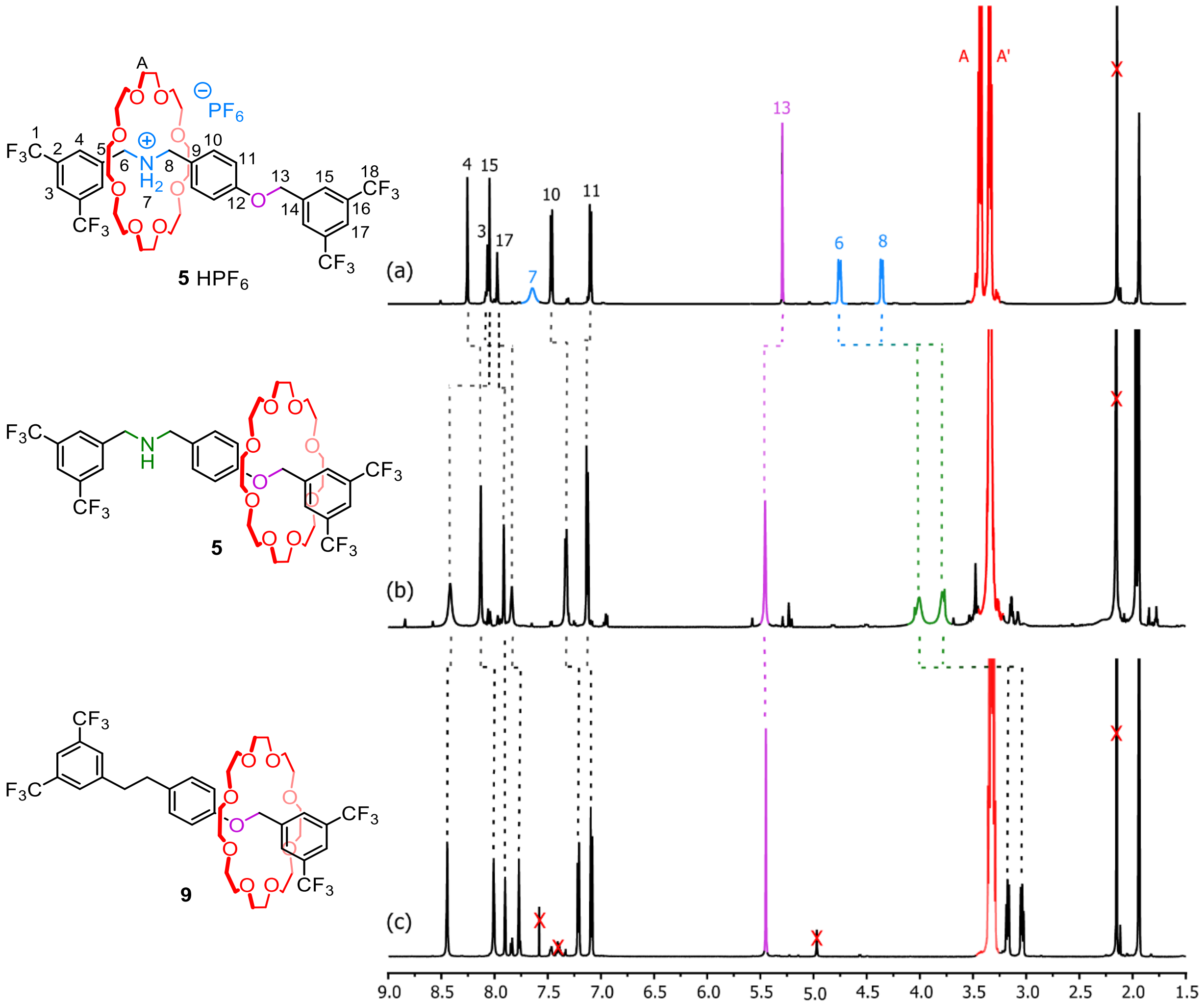

Figure S4. ^{1}H NMR spectra (CD_3CN, 600 MHz, 298 K) of: (a) protonated [2]rotaxane **5**·HPF$_6$, (b) deprotonated [2]rotaxane **5**, (c) [2]rotaxane **9** (after nitrogen deletion).

4.5. ^{1}H NMR comparison of rotaxanes **14**·HPF$_6$, **14** and **17**

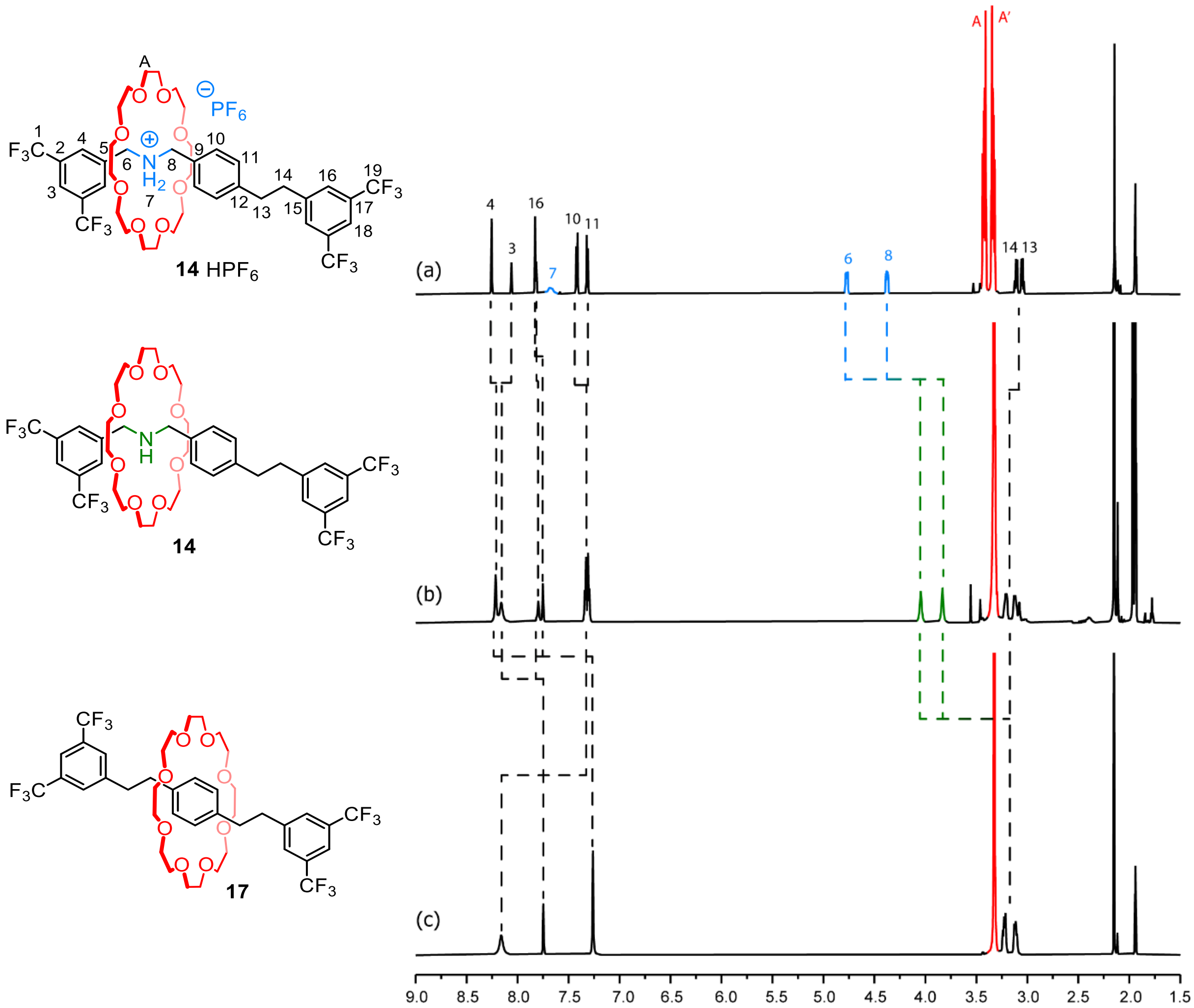

Figure S5. ^{1}H NMR spectra (CD_3CN, 600 MHz, 298 K) of: (a) protonated [2]rotaxane **14**·HPF$_6$, (b) deprotonated [2]rotaxane **14**, (c) [2]rotaxane **17** (after nitrogen deletion).

5. Limitations

5.1. Summary

In the course of our investigations, we uncovered some limitations of the methodology.[4]

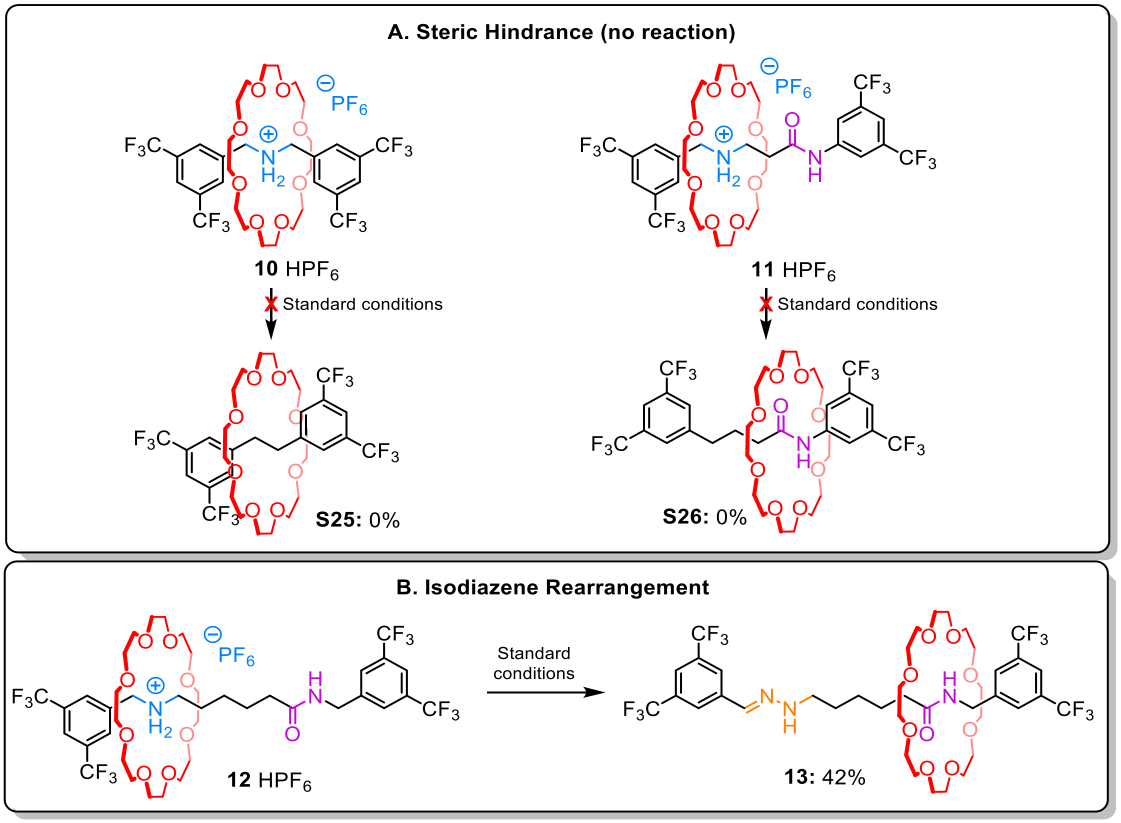

Figure S6. Examples of: (A) unreactive rotaxanes due to steric hindrance from the crown ether macrocycle, and (B) competitive isodiazene rearrangement.

[4] These limitations are line with observations made in Levin's original report, see Supporting Information of reference 10a

5.2. Synthetic schemes

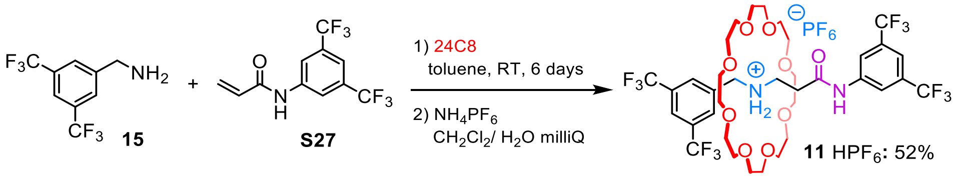

Scheme S7. Synthetic pathway to amide-containing rotaxane **11**.

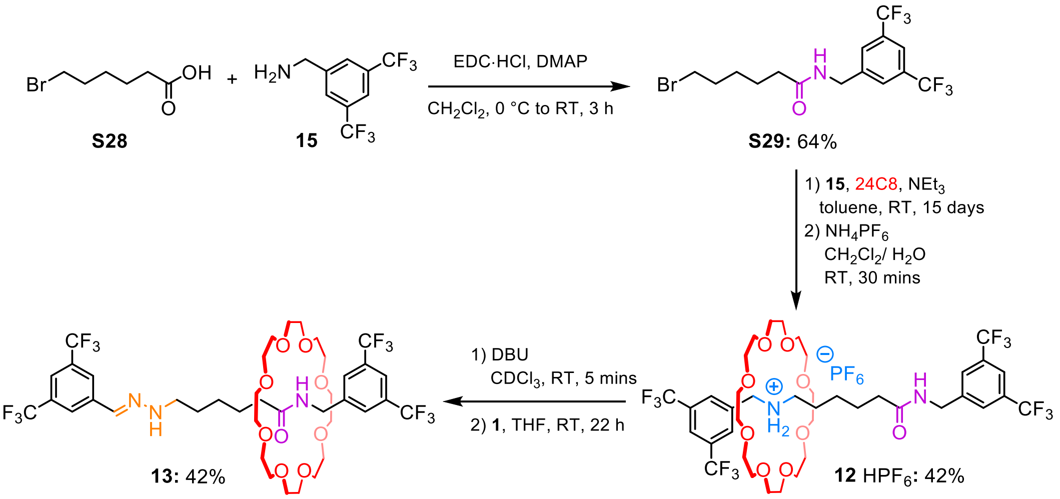

Scheme S8. Synthetic pathway to hydrazone-containing rotaxane **13**.

5.3. Experimental procedures and characterization data

11·HPF_6

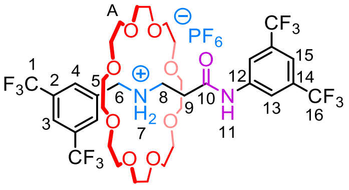

A solution of 3,5-bis(trifluoromethyl)benzylamine **15** (486 mg, 2.00 mmol, 2 eq.), 24C8 (352 mg, 1.00 mmol, 1 eq.) and **S27** (284 mg, 1.00 mmol, 1 eq.) in dry toluene (8 mL), was stirred at room temperature, under nitrogen atmosphere, for 6 days. The solvent was removed under reduced pressure and the residue was dissolved in EtOAc (10 mL). The organic solution was washed with a 1M aqueous solution of NaOH (1 × 10 mL). The aqueous layer was extracted with EtOAc (1 × 10 mL). The combined organic layers were evaporated under reduced pressure and the residue was dissolved in $CHCl_3$ (10 mL). The organic solution was washed with a saturated aqueous solution of NH_4PF_6 (1 × 10 mL). The aqueous layer was extracted with CH_2Cl_2 (1 × 10 mL). The combined organic layers were dried over $MgSO_4$, filtered, and the solvent was removed under reduced pressure. The residue was purified by automated flash column chromatography (SiO_2, CH_2Cl_2/EtOAc 100:0 to 45:55) to yield **11**·HPF_6 (532 mg, 52%) as a colorless solid.

^{1}H NMR (600 MHz, CD_3CN, 298 K) δ 8.97 (s, 1H, H_{11}), 8.30 (s, 2H, H_4), 8.16 (s, 2H, H_{13}), 8.08 (s, 1H, H_3), 7.74 (s, 1H, H_{15}), 7.61 – 7.50 (m, 2H, H_7), 4.77 – 4.71 (m, 2H, H_6), 3.62 – 3.52 (m, 18H, H_8, H_A), 3.41 – 3.35 (m, 16H, $H_{A'}$), 2.88 (t, ^3J = 6.9 Hz, 2H, H_9).

^{13}C NMR (151 MHz, CD_3CN, 298 K) δ 169.29 (C_{10}), 141.08 (C_{12}), 136.40 (C_5), 133.58 (q, $^3J_{C\text{-}F}$ = 3.7 Hz, C_4), 132.70 (q, $^2J_{C\text{-}F}$ = 33.3 Hz, C_{14}), 131.72 (q, $^2J_{C\text{-}F}$ = 33.2 Hz, C_2), 124.51 (q, $^1J_{C\text{-}F}$ = 272.1 Hz, C_1), 124.37

(q, $^1J_{C\text{-}F}$ = 271.9 Hz, C_{16}), 123.93 (sept, $^3J_{C\text{-}F}$ = 3.8 Hz, C_3), 120.23 (q, $^3J_{C\text{-}F}$ = 4.2 Hz, C_{13}), 118.32 (C_{12}) 71.27 (C_A), 52.00 (C_6), 45.54 (C_8), 33.81 (C_9).
HRMS (ESI^+): calculated for $C_{36}H_{47}F_{12}N_2O_9$ $[M\text{-}PF_6]^+$: 879.3084, found 879.3062.

S29

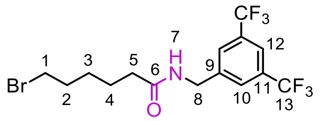

To a solution of 6-bromohexanoic acid **S28** (160 mg, 0.82 mmol, 1 eq.) and 3,5-bis(trifluoromethyl)benzylamine **15** (200 mg, 0.82 mmol, 1 eq.) in CH_2Cl_2 (10 mL) at 0 °C, was added DMAP (200 mg, 1.64 mmol, 2 eq.) and EDC·HCl (236 mg, 1.23 mmol, 1.5 eq.). The reaction mixture was allowed to warm to room temperature, and stirred under a nitrogen atmosphere, for 3 hours. The organic solution was successively washed with a 1M aqueous solution of HCl (3 × 10 mL) and a saturated aqueous solution of $NaHCO_3$ (2 × 10 mL). The organic layer was dried over $MgSO_4$, filtered, and the solvent was removed under reduced pressure, to yield **S29** (222 mg, 64%) as a colorless oil, which was used without further purification.
^{1}H NMR (600 MHz, $CDCl_3$, 298 K) δ 7.79 (s, 1H, H_{12}), 7.72 (s, 2H, H_{10}), 5.98 – 5.92 (m, 1H, H_7), 4.57 (d, ^3J = 6.2 Hz, 2H, H_8), 3.41 (t, ^3J = 6.7 Hz, 2H, H_1), 2.29 (t, ^3J = 7.5 Hz, 2H, H_5), 1.88 (quint, ^3J = 6.8 Hz, 2H, H_2), 1.72 (quint, ^3J = 7.4 Hz, 2H, H_4), 1.50 (quint, ^3J = 7.5 Hz, 2H, H_3).
^{13}C NMR (151 MHz, $CDCl_3$, 298 K) δ 172.99 (C_6), 141.30 (C_9), 132.10 (q, $^2J_{C\text{-}F}$ = 33.5 Hz, C_{11}), 127.84 (q, $^3J_{C\text{-}F}$ = 3.9 Hz, C_{10}), 123.32 (q, $^1J_{C\text{-}F}$ = 272.6 Hz, C_{13}), 121.60 (sept, $^3J_{C\text{-}F}$ = 3.9 Hz, C_{12}), 42.78 (C_8), 36.38 (C_5), 33.65 (C_1), 32.51 (C_2), 27.85 (C_3), 24.79 (C_4).
HRMS (ESI^+): calculated for $C_{15}H_{16}F_6BrNONa$ $[M+Na]^+$: 442.0212, found 442.0208.

13·HPF_6

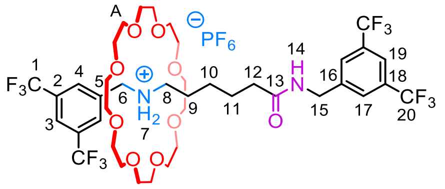

A solution of 3,5-bis(trifluoromethyl)benzylamine **15** (124 mg, 0.51 mmol, 1 eq.), 24C8 (180 mg, 0.51 mmol, 1 eq.), **S29** (215 mg, 0.51 mmol, 1 eq.) and NEt_3 (707 µL, 5.10 mmol, 10 eq.) in dry toluene (5 mL), was stirred at room temperature, under nitrogen atmosphere, for 15 days. The solvent was removed under reduced pressure and the residue was dissolved in CH_2Cl_2 (10 mL). A saturated aqueous solution of NH_4PF_6 (10 mL) was added, and the biphasic solution was vigorously stirred at room temperature, for 20 minutes. The two layers were separated, and the aqueous layer was extracted with CH_2Cl_2 (3 × 10 mL). The combined organic layers were dried over $MgSO_4$, filtered, and the solvent was removed under reduced pressure. The residue was purified by automated flash column chromatography (SiO_2, $CHCl_3$/MeOH 100:0 to 98:2) to yield **13**·HPF_6 (233 mg, 42%) as a colorless solid
^{1}H NMR (600 MHz, $CDCl_3$, 298 K) δ 8.19 (s, 2H, H_4), 7.91 (s, 1H, H_3), 7.78 (s, 2H, H_{17}), 7.70 (s, 1H, H_{19}), 7.40 – 7.32 (m, 2H, H_7), 7.13 (t, ^3J = 6.3 Hz, 1H, H_{14}), 4.73 – 4.67 (m, 2H, H_6), 4.49 (d, ^3J = 6.2 Hz, 2H, H_{15}), 3.62 – 3.55 (m, 16H, H_A), 3.42 – 3.35 (m, 16H, $H_{A'}$), 3.17 – 3.09 (m, 2H, H_8), 2.36 (t, ^3J = 7.5 Hz, 2H, H_{12}), 1.74 (quint, ^3J = 7.5 Hz, 2H, H_{11}), 1.70 (quint, ^3J = 7.9 Hz, 2H, H_9), 1.47 (quint, ^3J = 7.4 Hz, 2H, H_{10}).
^{13}C NMR (151 MHz, $CDCl_3$, 298 K) δ 173.81 (C_{13}), 142.29 (C_{16}), 135.77 (C_5), 132.31 (q, $^3J_{C\text{-}F}$ = 3.8 Hz, C_4), 131.48 (q, $^2J_{C\text{-}F}$ = 34.1 Hz, C_{18}), 131.41 (q, $^2J_{C\text{-}F}$ = 33.0 Hz, C_2), 128.04 (q, $^3J_{C\text{-}F}$ = 3.7 Hz, C_{17}), 123.57 (q, $^1J_{C\text{-}F}$ = 272.9 Hz, C_{20}), 123.33 (q, $^1J_{C\text{-}F}$ = 272.9 Hz, C_1), 122.57 (sept, $^3J_{C\text{-}F}$ = 3.7 Hz, C_3), 120.87 (sept,

$^{3}J_{C\text{-}F}$ = 4.0 Hz, C_{19}), 70.59 (C_A), 50.88 (C_6), 49.53 (C_8), 42.65 (C_{15}), 35.76 (C_{12}), 26.43 (C_9), 26.04 (C_{10}), 25.26 (C_{11}).
HRMS (ESI^+): calculated for $C_{40}H_{55}F_{12}N_2O_9$ $[M\text{-}PF_6]^+$: 935.3710, found 935.3710.

13

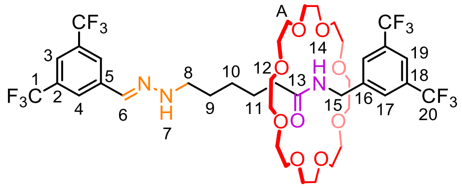

To a solution of **12**·HPF_6 (12.5 mg, 0.0116 mmol, 1 eq.) in $CDCl_3$ (700 µL), was added DBU (1.80 µL, 0.0116 mmol, 1 eq.). The reaction mixture was stirred at room temperature for 5 minutes. The solvent was evaporated under reduced pressure at room temperature, to give **12** (assumed quant.) which was used directly without further purification.
A solution of **1** (13.8 mg, 0.0348 mmol, 3 eq.) in dry and degassed THF (100 µL), was added to the freshly deprotonated rotaxane. The reaction mixture was stirred at room temperature, under nitrogen atmosphere, for 22 hours. The solvent was removed under reduced pressure at room temperature. The residue was directly purified by automated flash column chromatography (SiO_2, $CHCl_3$/MeOH 100:0 to 98:2) to yield **13** (4.5 mg, 42%) as a colorless film.
^{1}H NMR (600 MHz, $CDCl_3$, 298 K) δ 8.61 (s, 2H, H_{17}), 7.93 (s, 2H, H_4), 7.70 (s, 1H, H_{19}), 7.67 (s, 1H, H_3), 7.51 (s, 1H, H_6), 7.21 (t, ^{3}J = 4.2 Hz, 1H, H_{14}), 4.69 (d, ^{3}J = 4.0 Hz, 2H, H_{15}), 3.53 – 3.43 (m, 16H, H_A), 3.28 (t, ^{3}J = 7.0 Hz, 2H, H_8), 3.25 – 3.18 (m, 16H, $H_{A'}$), 2.17 (t, ^{3}J = 7.6 Hz, 2H, H_{12}), 1.73 – 1.62 (m, 4H, H_9, H_{11}), 1.44 (quint, ^{3}J = 7.7 Hz, 2H, H_{10}).
^{13}C NMR (151 MHz, $CDCl_3$, 298 K) δ 172.10 (C_{13}), 141.94 (C_{16}), 138.95 (C_5), 134.42 (C_{17}), 131.85 (q, $^{2}J_{C\text{-}F}$ = 33.0 Hz, C_{18}), 130.65 (C_6), 129.28 (q, $^{2}J_{C\text{-}F}$ = 32.0 Hz, C_2), 125.12 (C_4), 124.25 (q, $^{1}J_{C\text{-}F}$ = 272.1 Hz, C_{20}), 123.55 (q, $^{1}J_{C\text{-}F}$ = 272.1 Hz, C_1), 120.35 (sept, $^{3}J_{C\text{-}F}$ = 5.0 Hz, C_3), 119.72 (sept, $^{3}J_{C\text{-}F}$ = 4.5 Hz, C_{18}), 70.69 (C_A), 48.07 (C_8), 43.59 (C_{15}), 36.08 (C_{12}), 28.06 (C_9), 27.14 (C_{10}), 25.21 (C_{11}).
HRMS (ESI^+): calculated for $C_{40}H_{53}F_{12}N_3O_9Na$ $[M+Na]^+$: 970.3482, found 970.3491.

5.4. ^{1}H NMR comparison of rotaxanes **12**·HPF$_6$, **12** and **13**

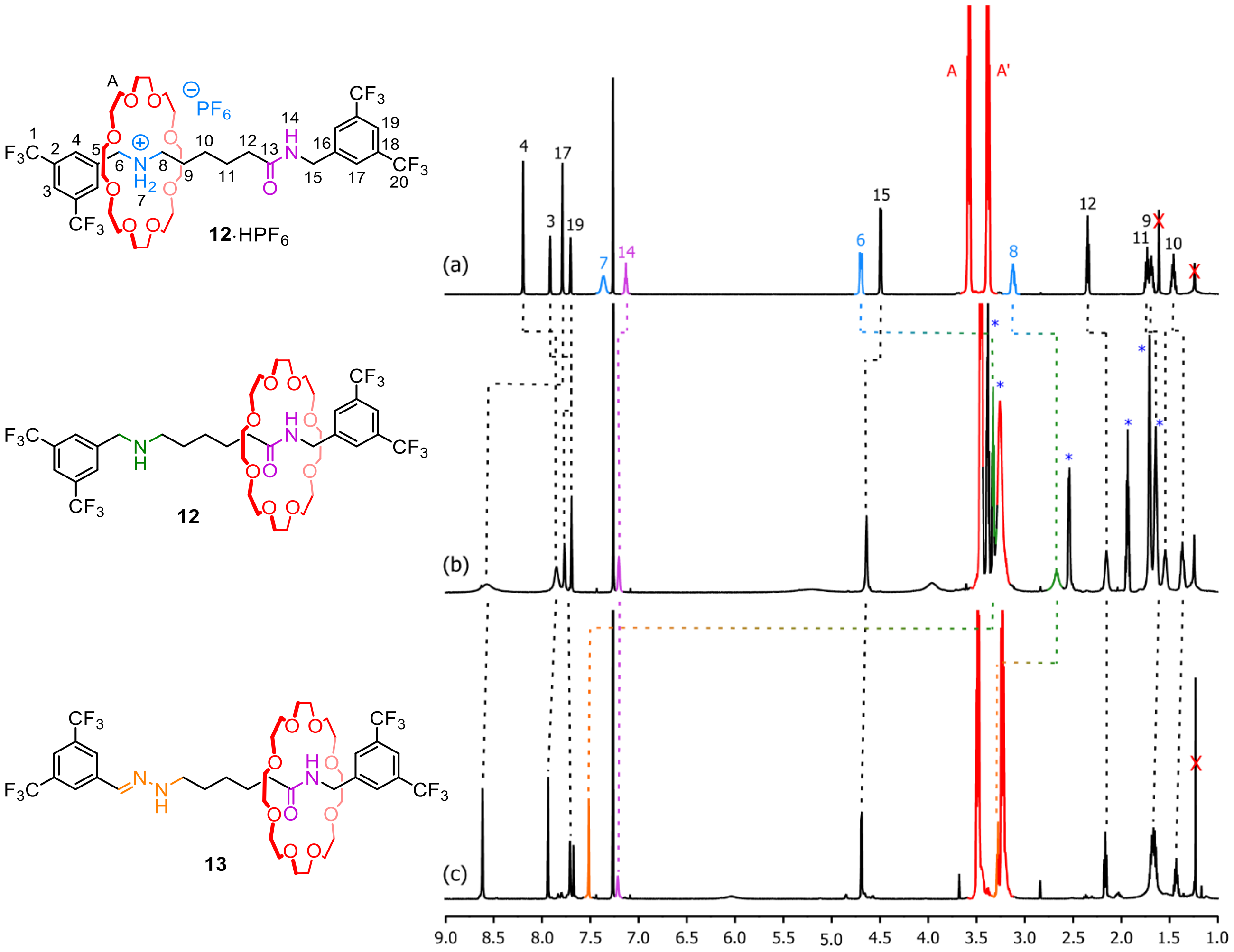

Figure S7. ^{1}H NMR spectra ($CDCl_3$, 600 MHz, 298 K) of: (a) [2]rotaxane **12**·HPF$_6$, (b) [2]rotaxane **12**, (c) hydrazone-containing [2]rotaxane **13**. DBU signals are marked with blue stars.

6. Comparison of Nitrogen Deletion Reagents 1 and *O*-diphenylphosphinylhydroxylamine (DPPH) 18

6.1. Synthetic scheme

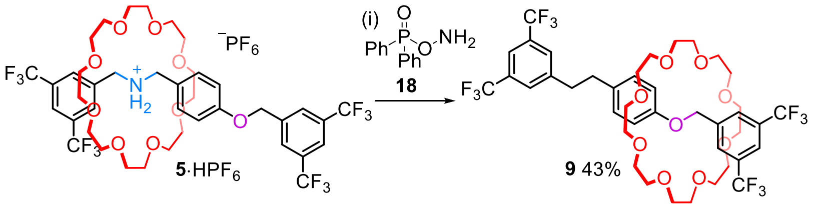

Scheme S9. Skeletal editing of **5**·HPF_6 with alternative nitrogen deletion reagent[23] *O*-diphenylphosphinylhydroxylamine (DPPH) **18**. Reagents and conditions: (i) 2-tert-Butylimino-2-diethylamino-1,3-dimethylperhydro-1,3,2-diazaphosphorine (BEMP), polymer-bound (5 equiv.), CH_3CN, RT, 2 h, then **18** (5 equiv.), K_2CO_3 (2 equiv.), THF/H_2O (1:1), RT, overnight, 43% over 2 steps.

6.2. Experimental procedure

9

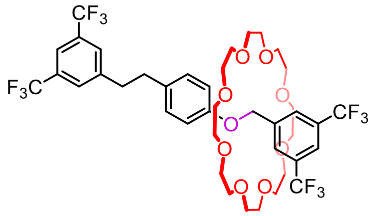

To a solution of **5**·HPF_6 (110 mg, 0.102 mmol, 1 eq.) in dry CH_3CN (2.7 mL), was added polymer-bound BEMP (235 mg, 0.517 mmol, 5 eq., 2.2 mmol/g). The reaction mixture was stirred at room temperature, under a nitrogen atmosphere, for 2 hours. The mixture was filtered, and the polymer resin was rinsed with dry CH_3CN. The filtrate was evaporated under reduced pressure, at room temperature, to give **5** (91 mg, 95%).
To a vial containing **5** (40.5 mg, 0.044 mmol, 1 eq.) was added THF (0.1 mL), H_2O (0.1 mL), K_2CO_3 (12.1 mg, 0.088 mmol, 2 eq.), and **18** (50.9 mg, 0.218 mmol, 5 eq.). The vial was closed, and the reaction mixture was stirred at 1400 rpm at room temperature for 24 hours. The reaction was diluted with H_2O (5 mL) and extracted with CH_2Cl_2 (10 mL). The aqueous layer was saturated with NaCl and extracted with CH_2Cl_2 (3 × 5 mL). The organic layers were combined, dried over $MgSO_4$, filtered, and the solvent was removed under reduced pressure. The residue was purified by automatic flash column chromatography (SiO_2, CH_2Cl_2/MeOH 100:0 to 99:1) to yield **9** (17.2 mg, 43%) as a colorless film.
The NMR data for the isolated product was identical to that obtained using **1** (Figure S8c).

6.3. ^{1}H NMR comparison of crude reaction mixtures and the isolated [2]rotaxane, **9**

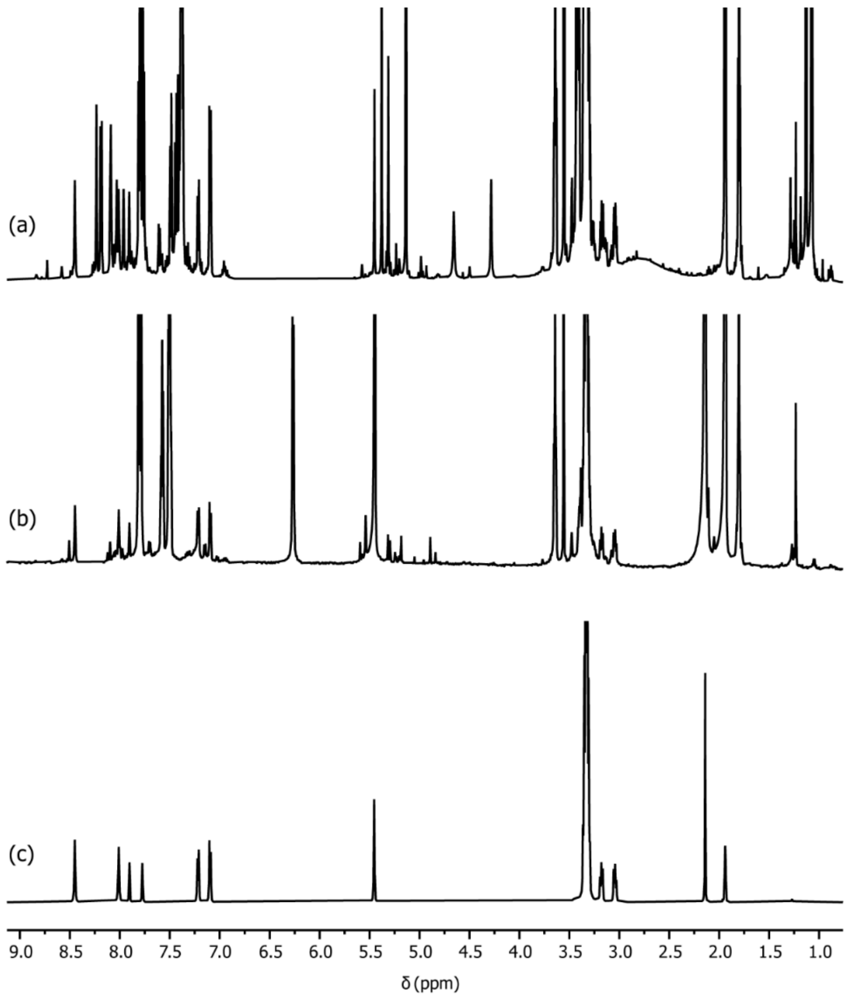

Figure S8. ^{1}H NMR spectra (CD_3CN, 600 MHz, 298 K) of: (a) crude reaction mixture after reaction of **5** with **1**, (b) crude reaction mixture after reaction of **5** with **18**, (c) isolated [2]rotaxane **9**.

7. X-Ray Crystallography

General Procedure – Crystals suitable for X-ray diffraction were grown by dissolving the [2]rotaxane **17** (70 mg) in 5 mL of Et_2O in a 25 mL round bottom flask. The flask was sealed with a septum pierced by a needle, and the solvent was allowed to slowly evaporate over a period of 1-2 weeks, resulting in the formation of crystals suitable for investigation by X-ray crystallography.

Data Collection, crystal structure determinations and refinements – A shaped crystal with dimensions 0.17×0.13×0.10 mm^3 was mounted on a suitable support. Data were collected using a SuperNova, Single source at offset/far, Eos diffractometer operating at T = 99.9(3) K.

Data were measured using ω scans of 0.8° per frame for 14.0 s using Mo K_α radiation. The diffraction pattern was indexed and the total number of runs and images was based on the strategy calculation from the program CrysAlisPro (Rigaku). The maximum resolution that was achieved was Θ = 26.411° (0.80 Å).

The diffraction pattern was indexed and the total number of runs and images was based on the strategy calculation from the program CrysAlisPro (Rigaku) and the unit cell was refined using CrysAlisPro (Rigaku, V1.171.43.91a, 2023) on 6956 reflections, 21% of the observed reflections.

Data reduction, scaling and absorption corrections were performed using CrysAlisPro (Rigaku, V1.171.43.91a, 2023). The final completeness is 99.70 % out to 26.411° in Θ. An analytical absorption correction was performed using CrysAlisPro 1.171.43.91a (Rigaku Oxford Diffraction, 2023). Analytical numeric absorption correction using a multifaceted crystal model based on expressions derived by R.C. Clark & J.S. Reid.(Clark, R. C. & Reid, J. S. (**1995**). *Acta Cryst.* A51, 887-897) using spherical harmonics, implemented in SCALE3 ABSPACK scaling algorithm. The absorption coefficient μ of this material is 0.132 mm^{-1} at this wavelength (λ = 0.711Å) and the minimum and maximum transmissions are 0.998 and 0.999.

The structure was solved, and the space group P-1 (# 2) determined by the ShelXT 2018/2 (Sheldrick, 2018) structure solution program using Intrinsic Phasing and refined by Least Squares using version 2019/3 of ShelXL 2019/3 (Sheldrick, 2015). All non-hydrogen atoms were refined anisotropically. Hydrogen atom positions were calculated geometrically and refined using the riding model. Hydrogen atom positions were calculated geometrically and refined using the riding model.

Table S1. Crystallographic parameters for the [2]rotaxane **17**.

**Formula**	$C_{84}H_{100}F_{24}O_{16}$
$D_{calc.}$ **/ g cm^{-3}**	1.431
μ **/ mm^{-1}**	0.132
**Formula Weight**	1821.63
**Size / mm^3**	0.17×0.13×0.10
T **/ K**	99.9(3)
**Crystal System**	Triclinic
**Space Group**	*P*-1
a **/Å**	11.4151(8)
b **/Å**	14.5396(9)
c **/Å**	26.4254(14)
α **/ °**	82.109(5)
β **/ °**	81.508(5)
γ **/ °**	78.742(5)
**V / Å^3**	4227.6(5)
Z	2
Z'	1
**Wavelength / Å**	0.71073
**Radiation type**	Mo K_α
Θ_{min} **/ °**	3.426
Θ_{max} **/ °**	26.411
**Measured Refl.**	32857
**Independent Refl.**	17262
**Reflections with I > 2(I)**	10212
R_{int}	0.0465
**Parameters**	1117
**Restraints**	0
**Largest Peak**	0.746
**Deepest Hole**	-0.420
**GooF**	1.021
wR_2 **(all data)**	0.2030
wR_2	0.1669
R_1 **(all data)**	0.1362
R_1	0.0751

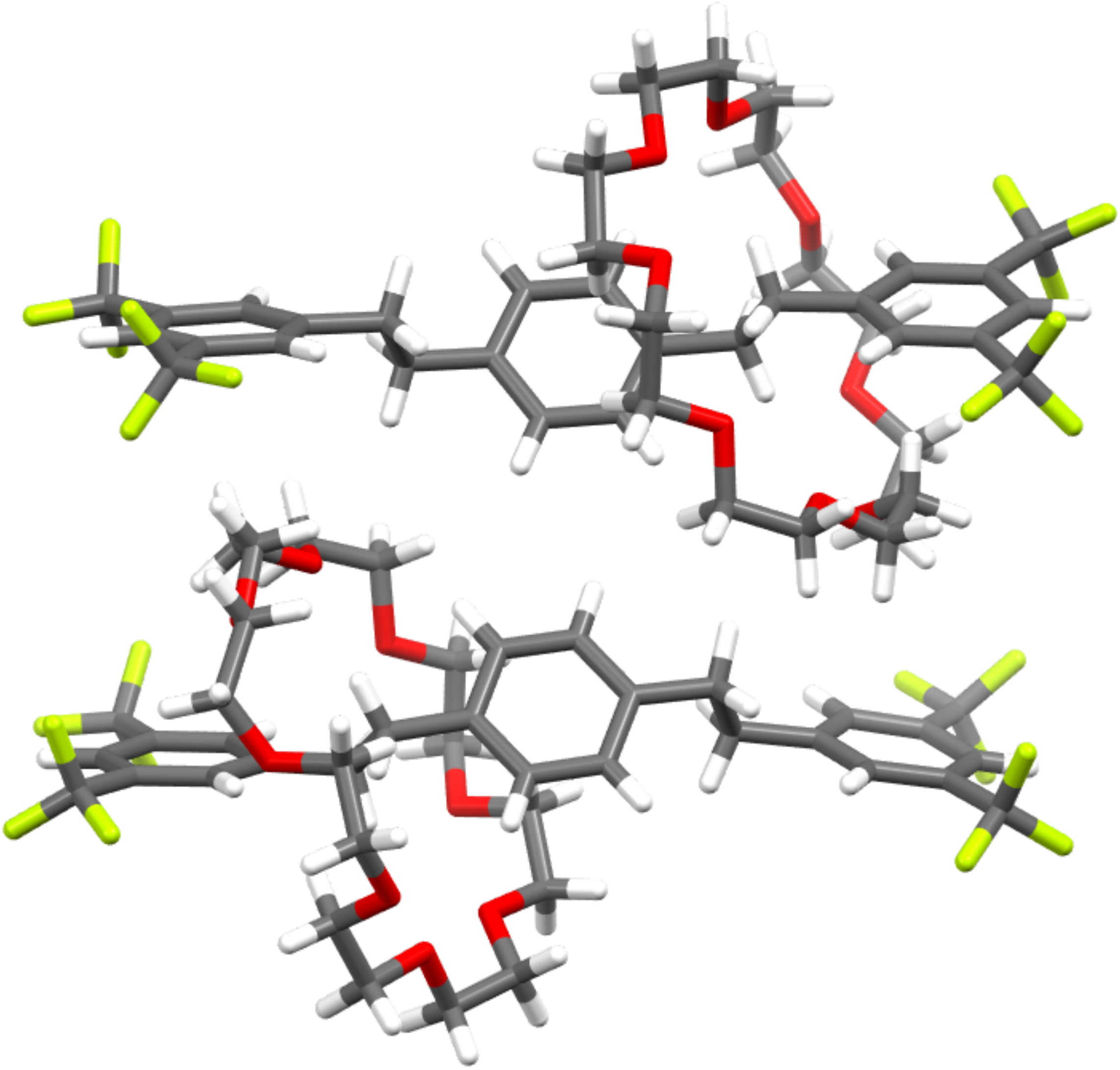

Figure S9. X-ray crystal structure of [2]rotaxane **17**.

8. NMR spectra

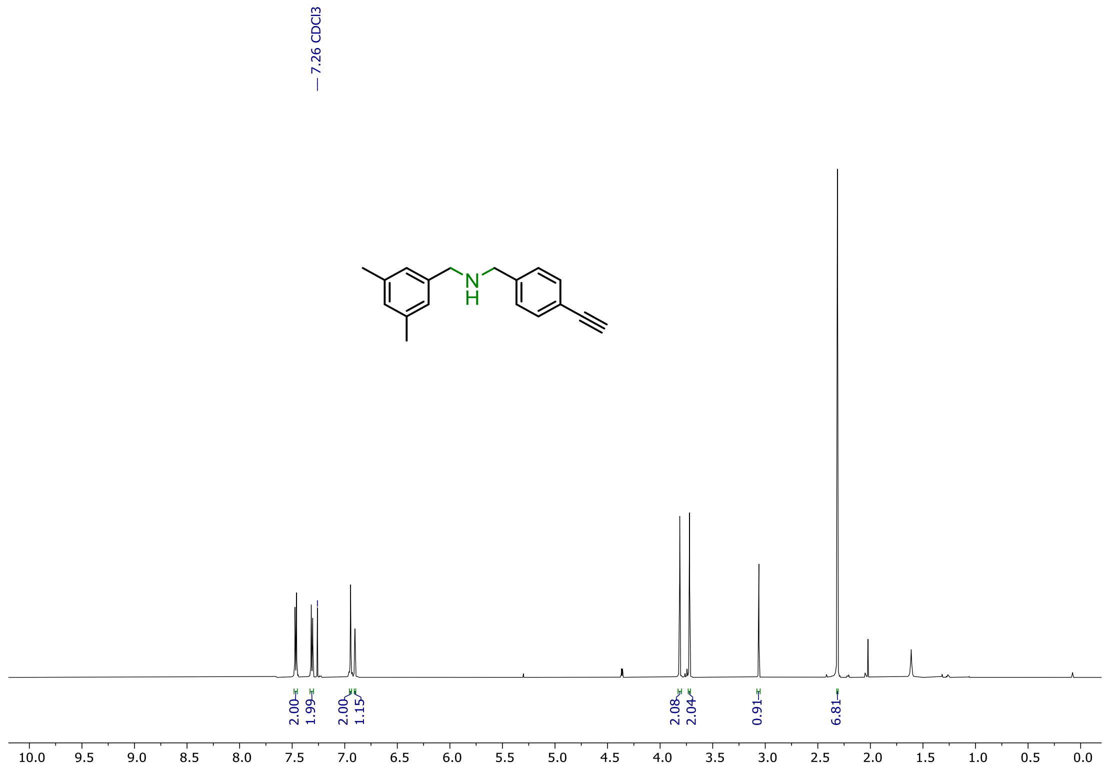

Spectrum S1. ^{1}H NMR spectrum (600 MHz, $CDCl_3$, 298 K) of compound **S3**.

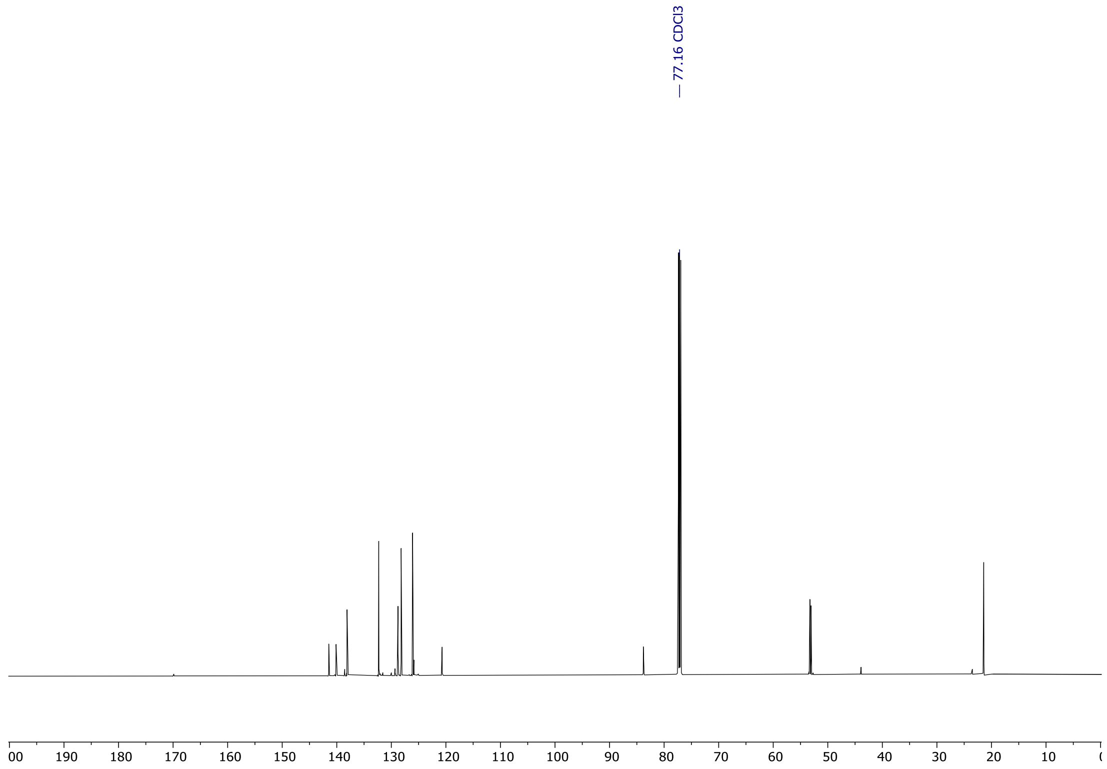

Spectrum S2. ^{13}C NMR spectrum (151 MHz, $CDCl_3$, 298 K) of compound **S3**.

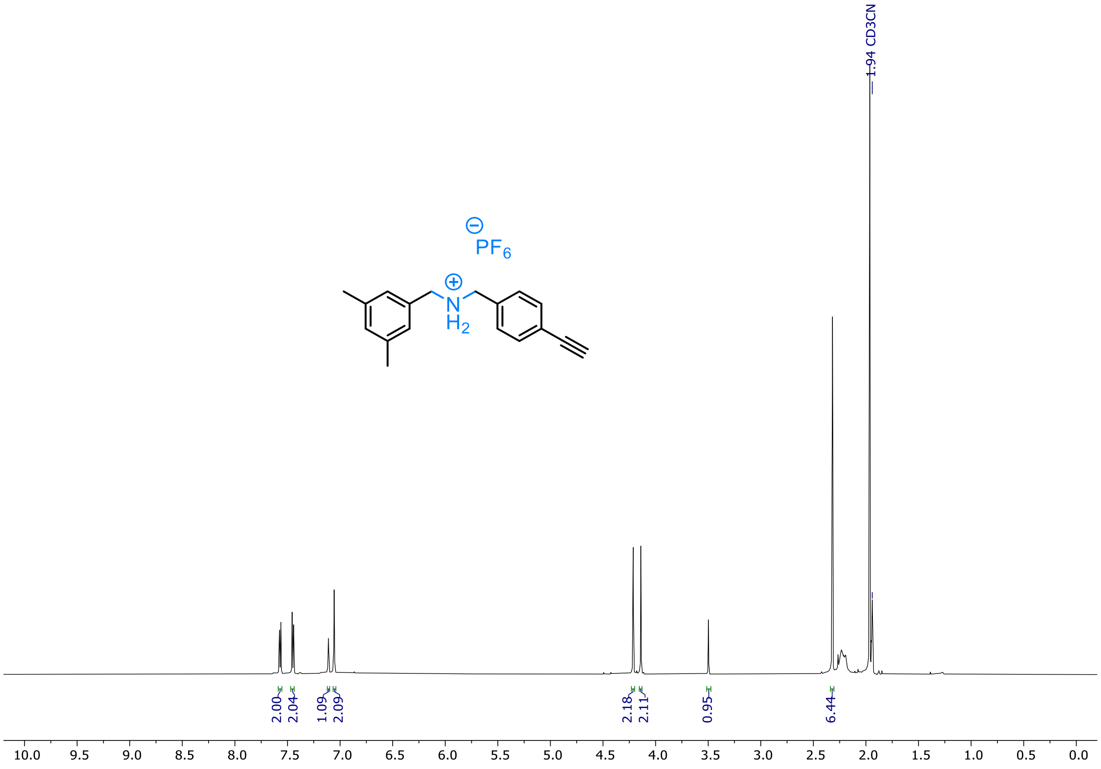

Spectrum S3. ^{1}H NMR spectrum (600 MHz, CD_3CN, 298 K) of compound **S4**.

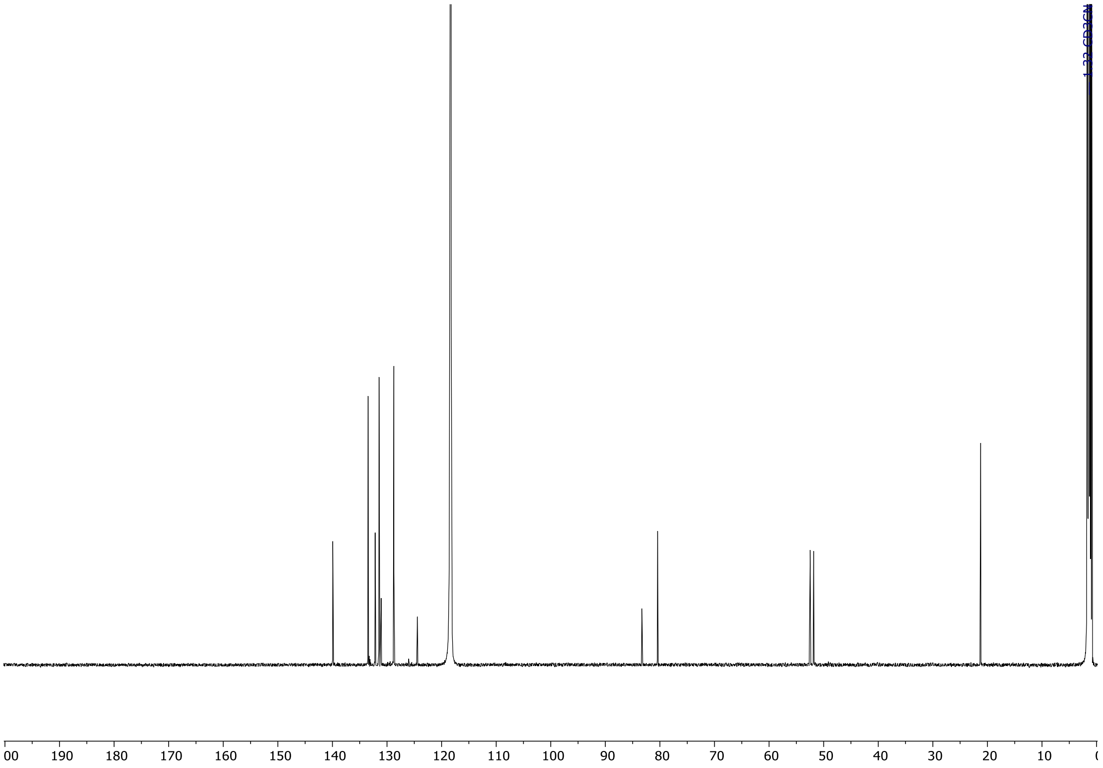

Spectrum S4. ^{13}C NMR spectrum (151 MHz, CD_3CN, 298 K) of compound **S4**.

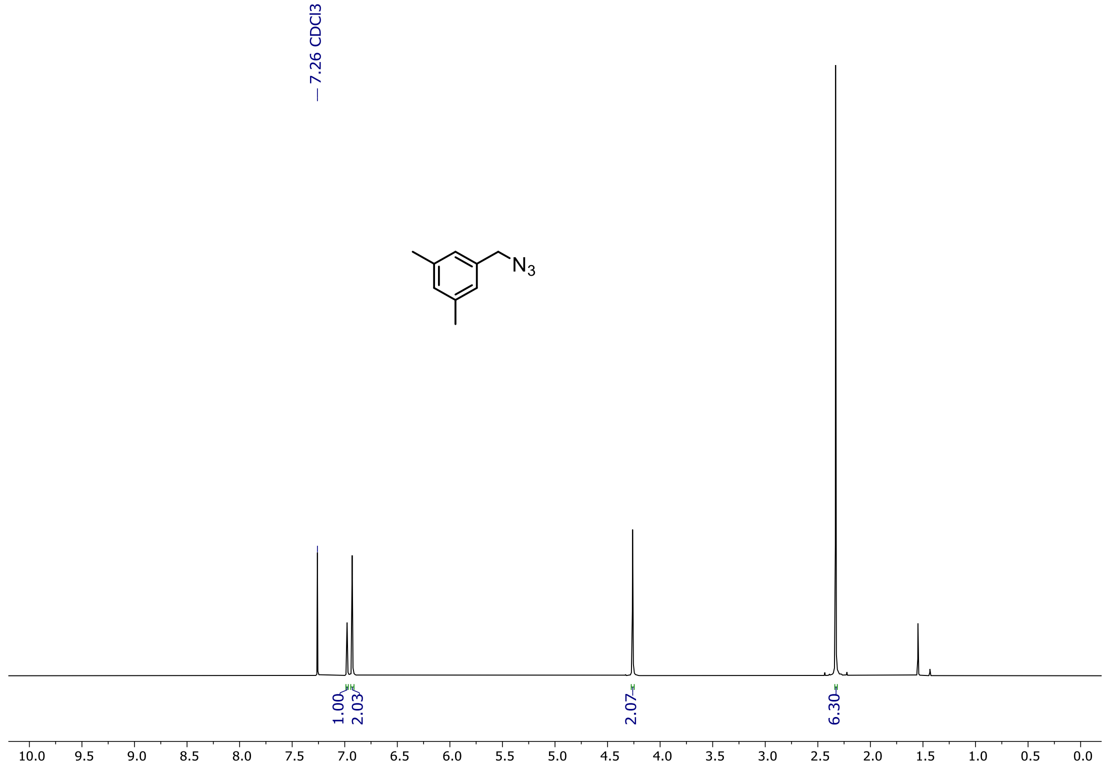

Spectrum S5. ^{1}H NMR spectrum (600 MHz, $CDCl_3$, 298 K) of compound **S6**.

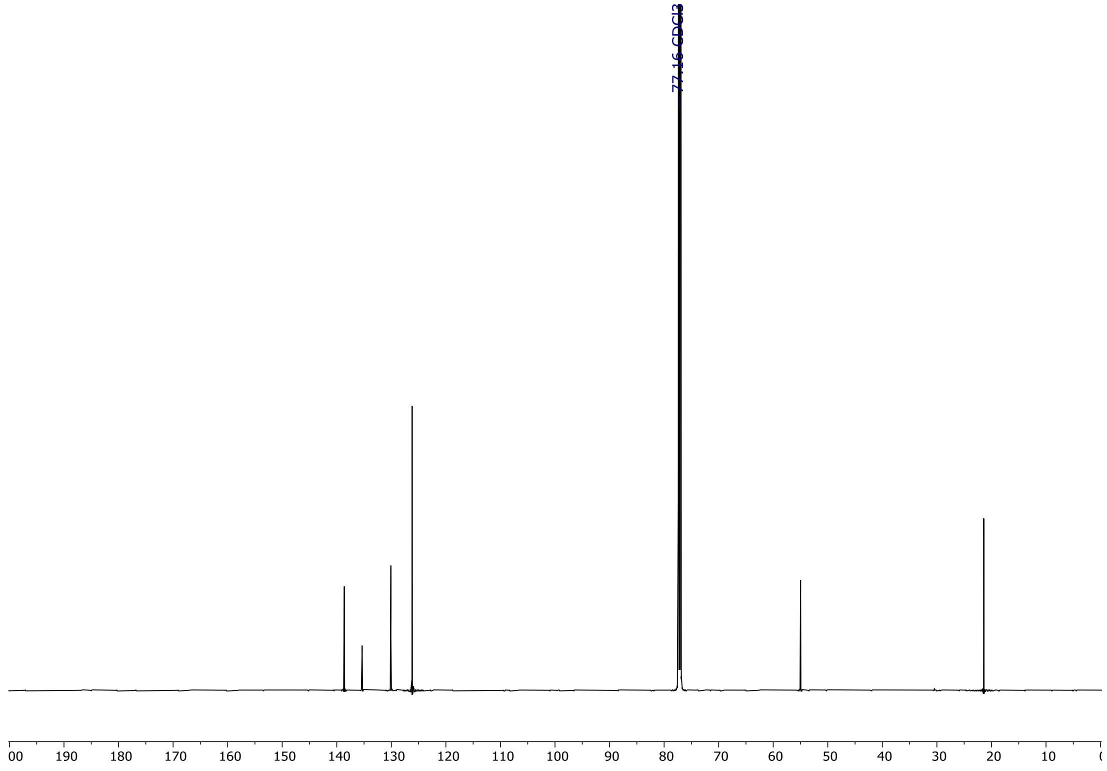

Spectrum S6. ^{13}C NMR spectrum (151 MHz, $CDCl_3$, 298 K) of compound **S6**.

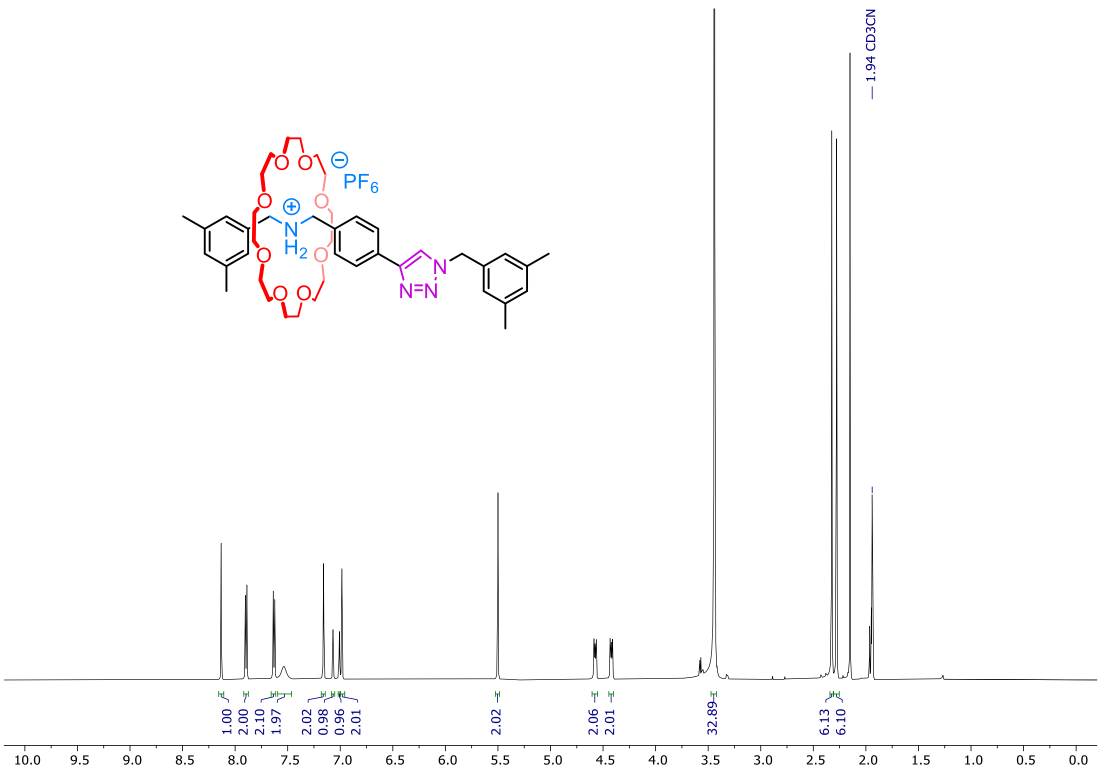

Spectrum S7. ^{1}H NMR spectrum (600 MHz, CD_3CN, 298 K) of compound **3**·HPF_6.

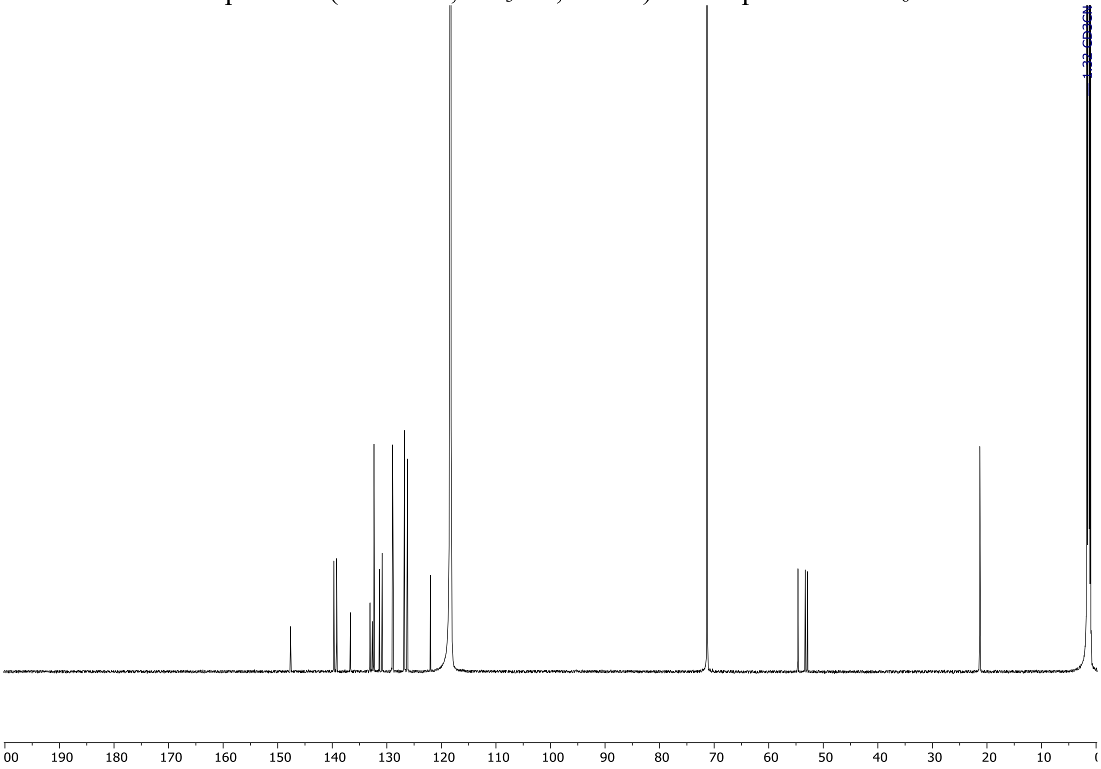

Spectrum S8. ^{13}C NMR spectrum (151 MHz, CD_3CN, 298 K) of compound **3**·HPF_6.

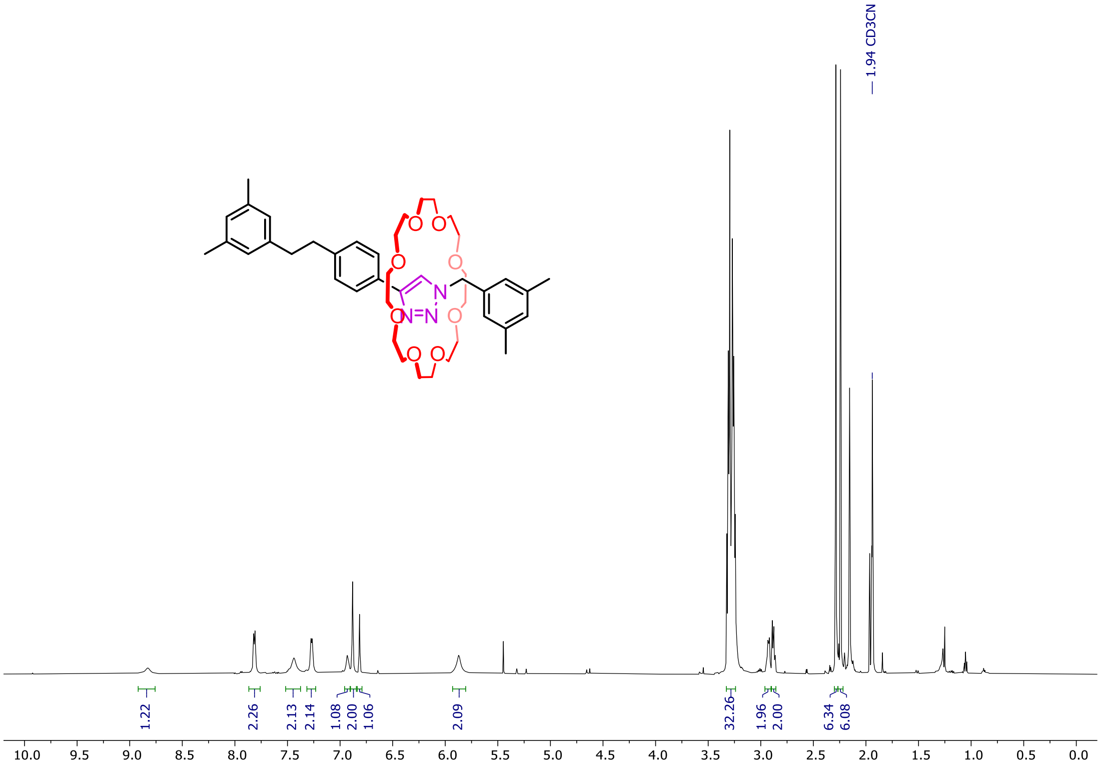

Spectrum S9. ^{1}H NMR spectrum (600 MHz, CD_3CN, 298 K) of compound **7**.

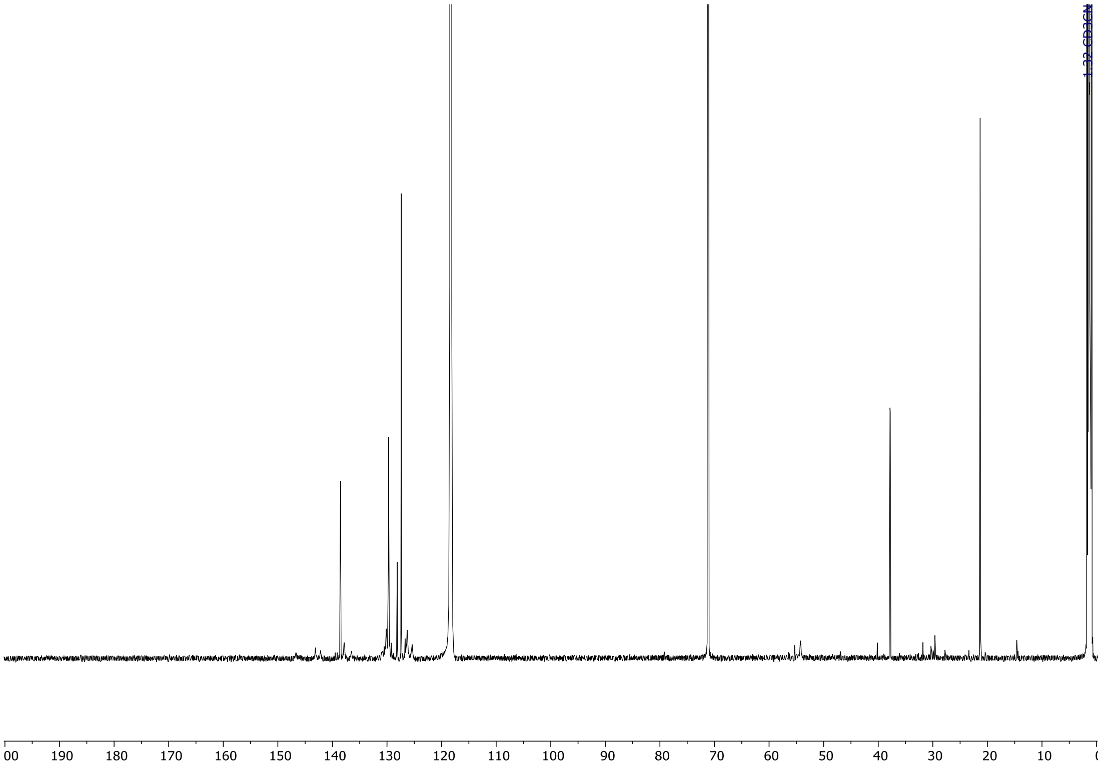

Spectrum S10. ^{13}C NMR spectrum (151 MHz, CD_3CN, 298 K) of compound **7**.

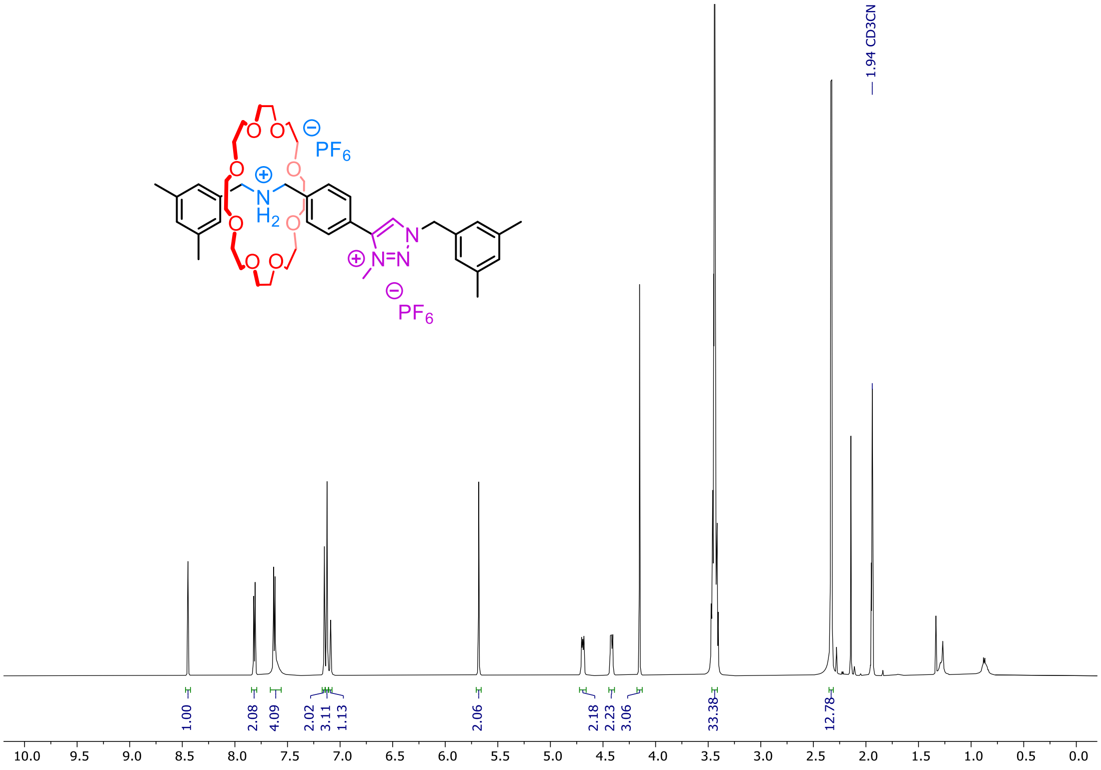

Spectrum S11. ^{1}H NMR spectrum (600 MHz, CD_3CN, 298 K) of compound **2**·HPF_6.

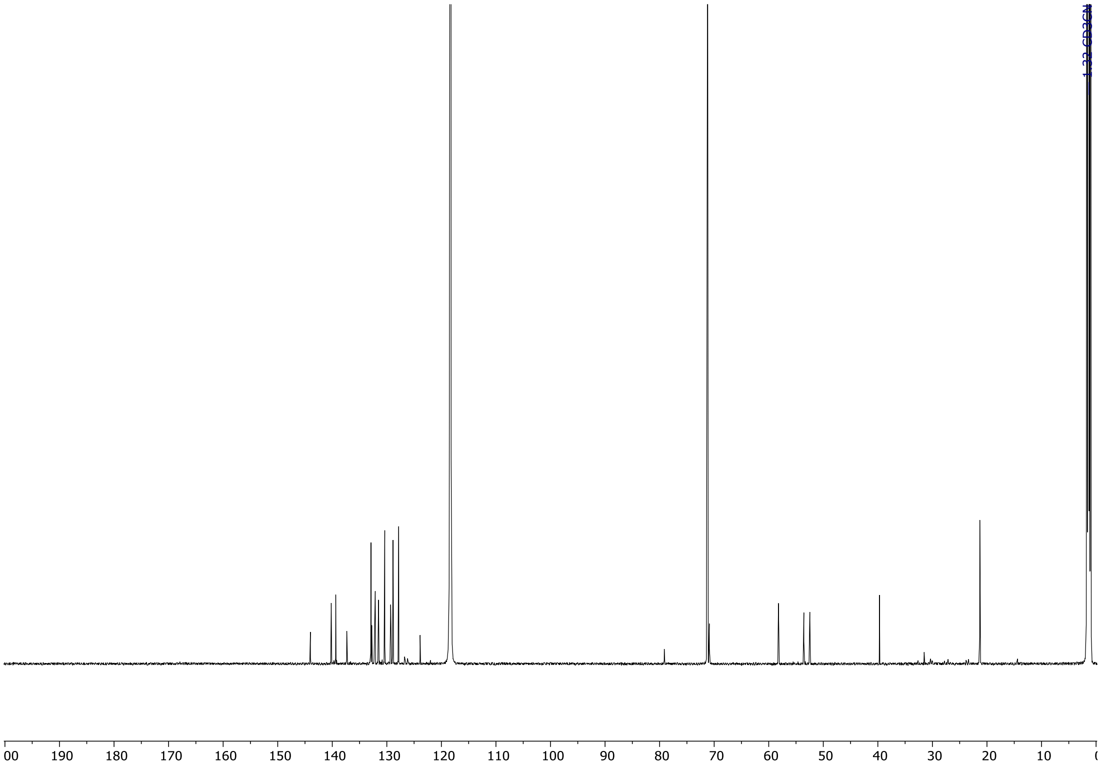

Spectrum S12. ^{13}C NMR spectrum (151 MHz, CD_3CN, 298 K) of compound **2**·HPF_6.

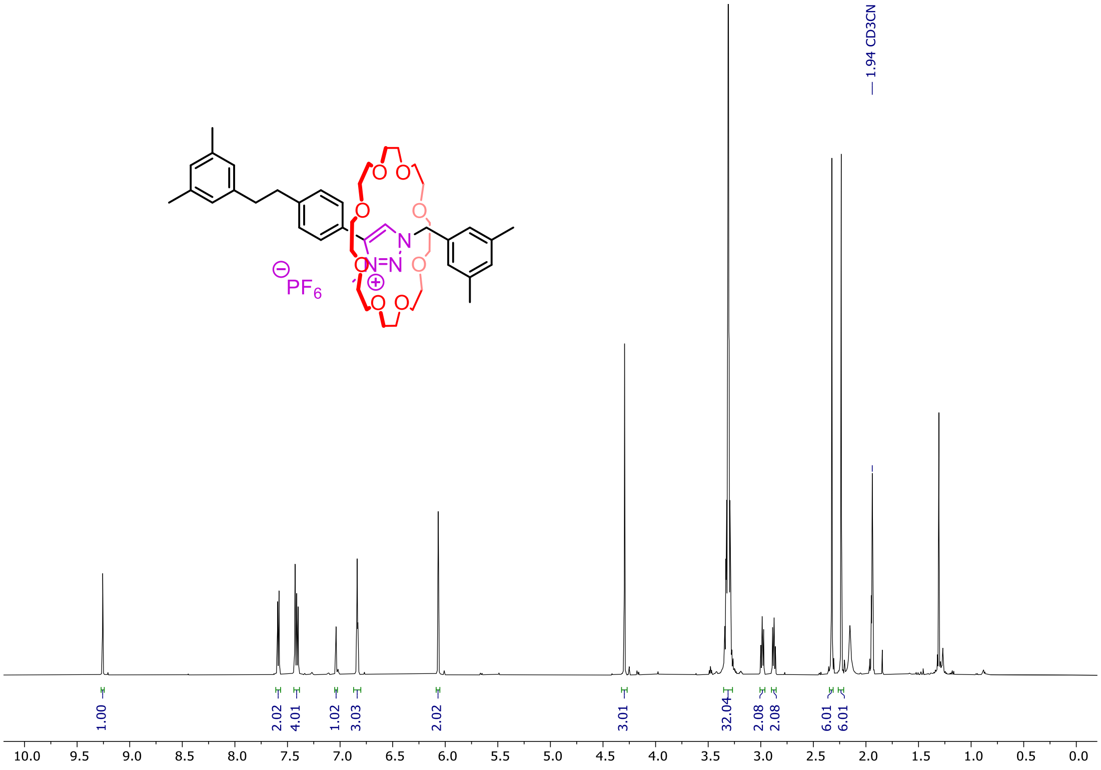

Spectrum S13. ^{1}H NMR spectrum (600 MHz, CD_3CN, 298 K) of compound **6**.

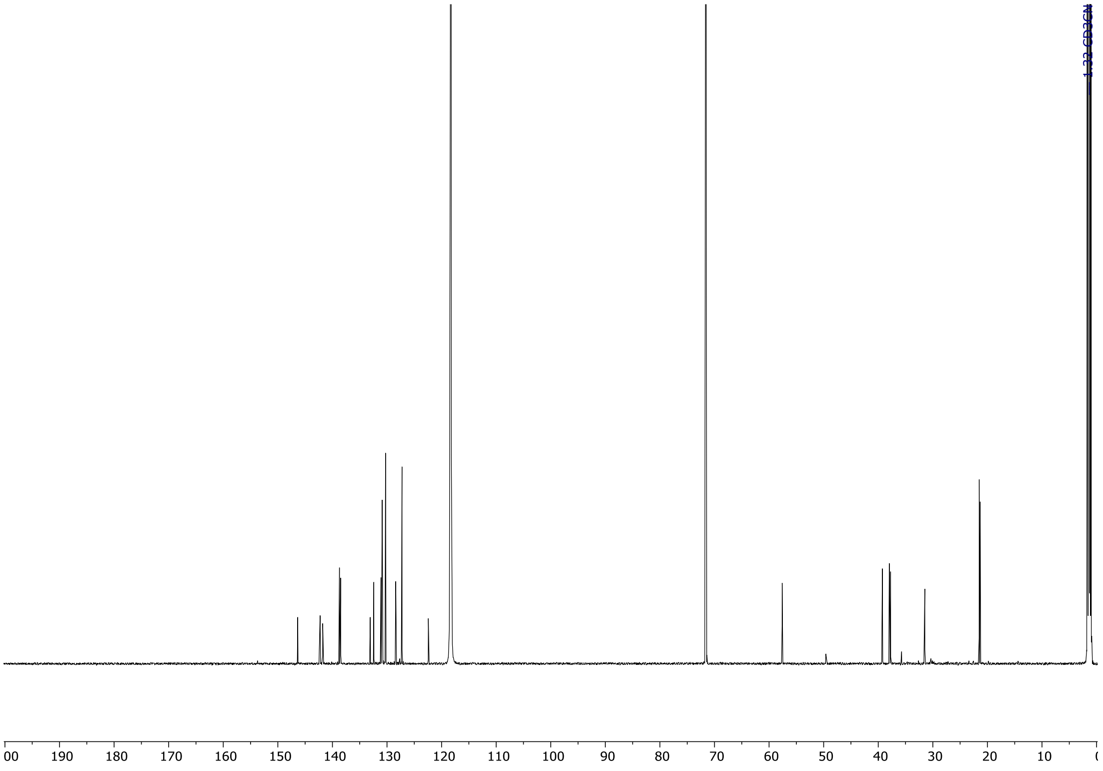

Spectrum S14. ^{13}C NMR spectrum (151 MHz, CD_3CN, 298 K) of compound **6**.

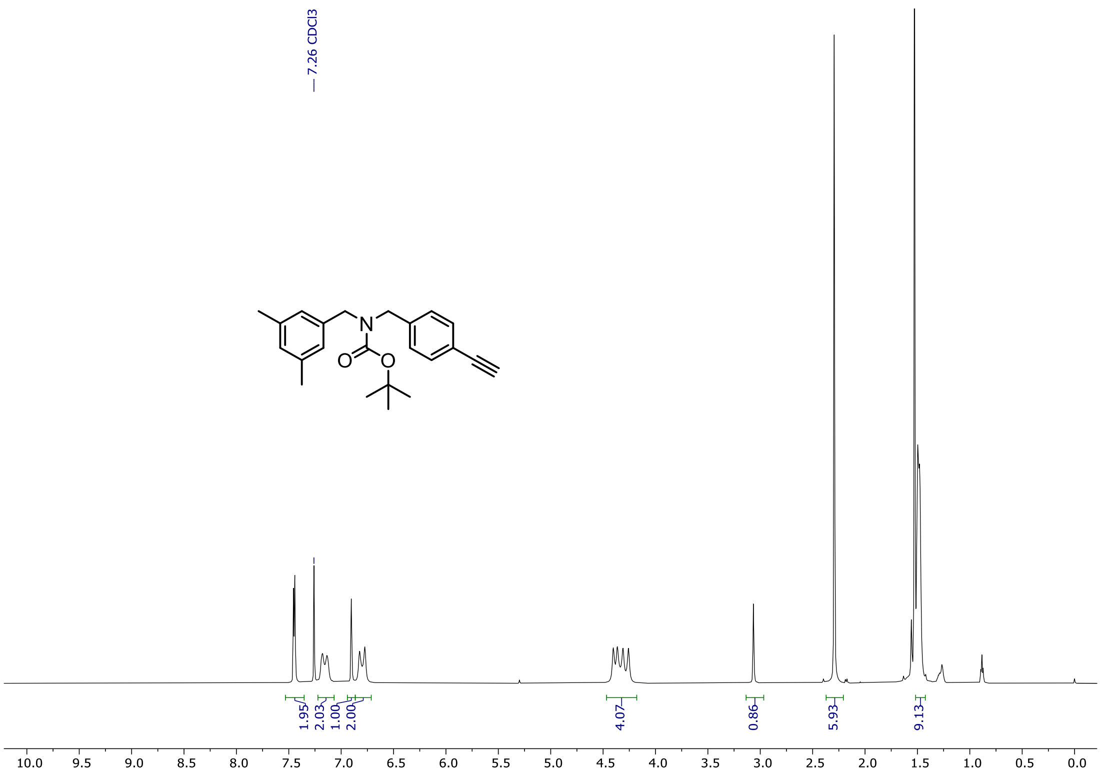

Spectrum S15. ^{1}H NMR spectrum (600 MHz, $CDCl_3$, 298 K) of compound **S7**.

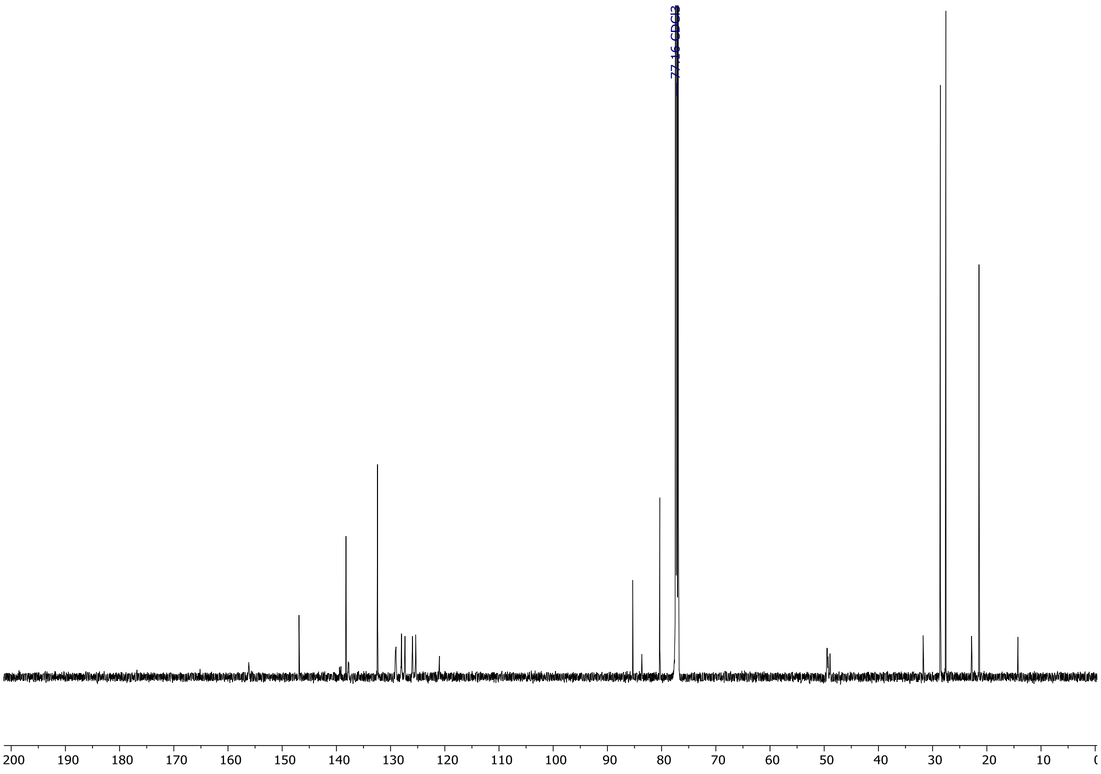

Spectrum S16. ^{13}C NMR spectrum (151 MHz, CD_3CN, 298 K) of compound **S7**.

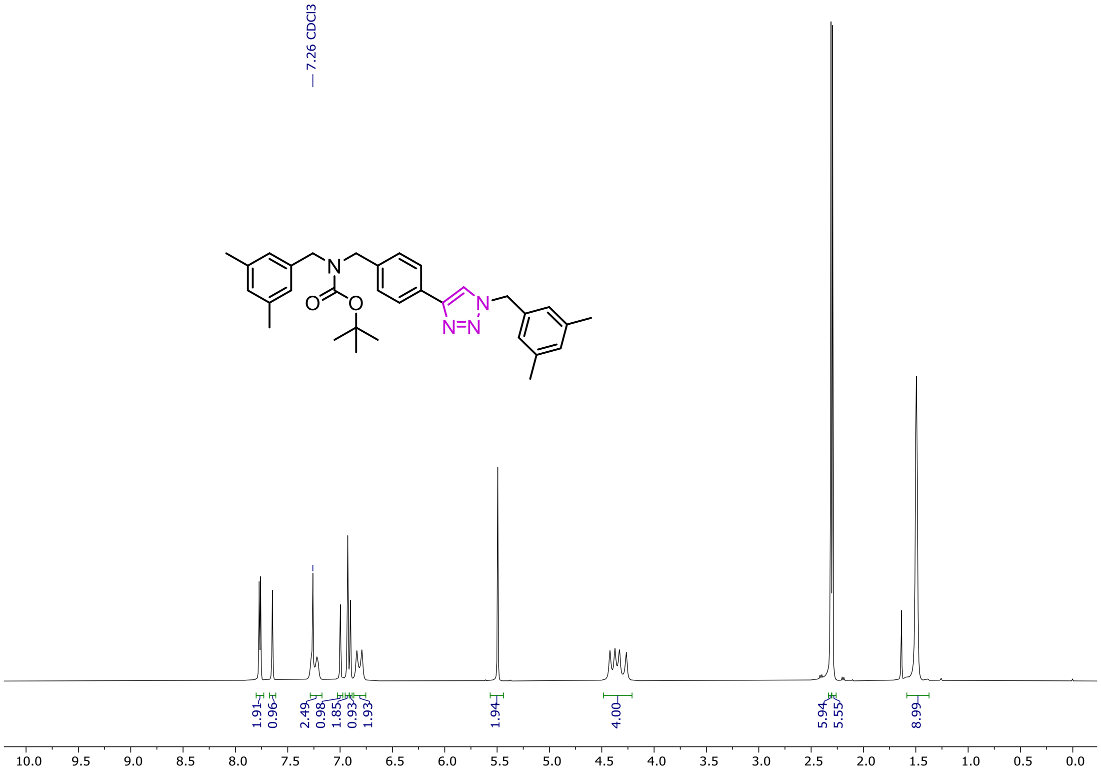

Spectrum S17. ^{1}H NMR spectrum (600 MHz, $CDCl_3$, 298 K) of compound **S8**.

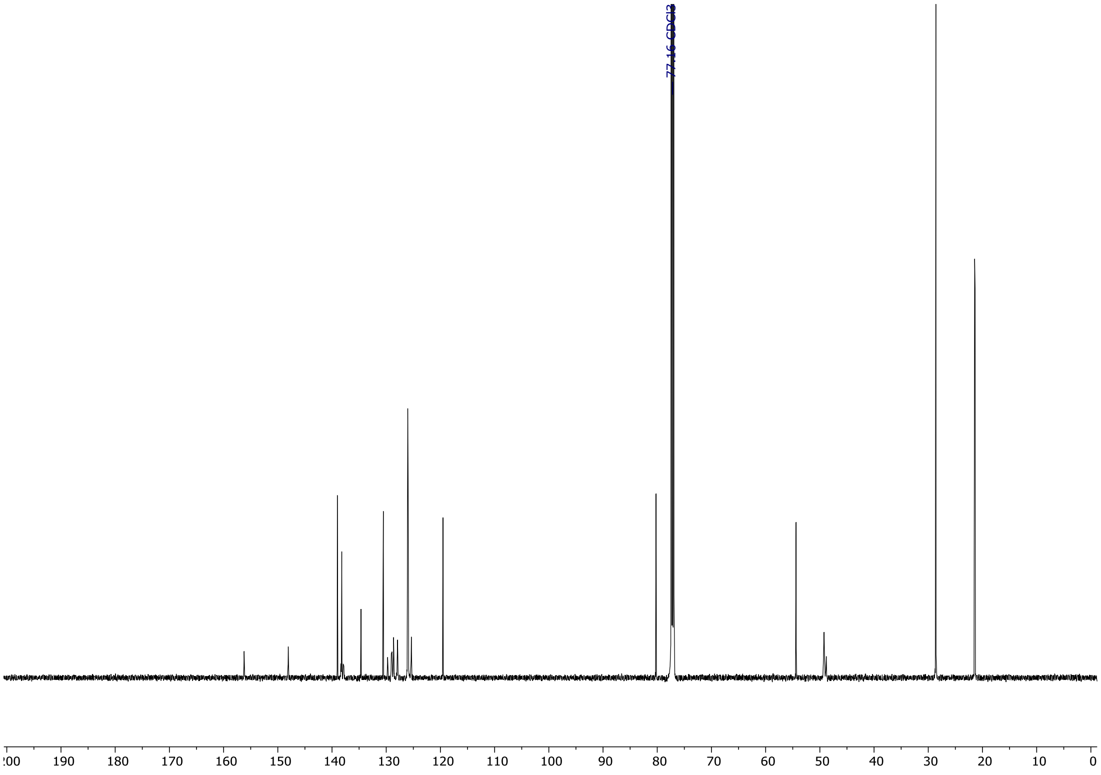

Spectrum S18. ^{13}C NMR spectrum (151 MHz, CD_3CN, 298 K) of compound **S8**.

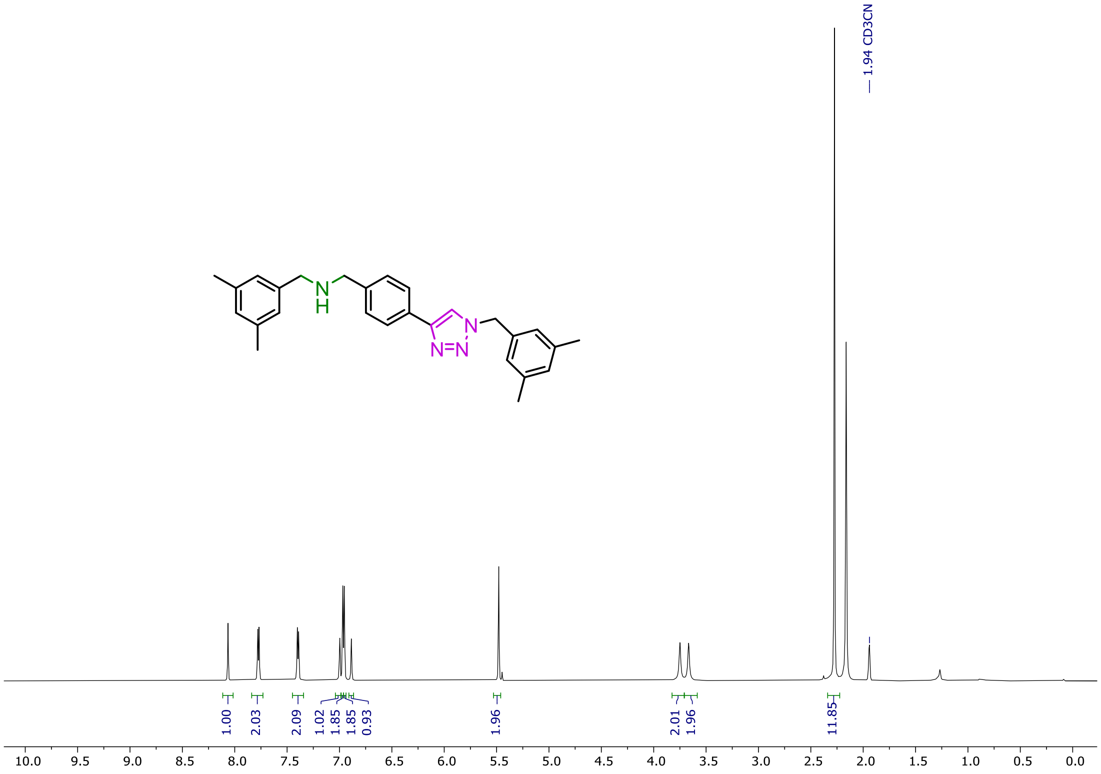

Spectrum S19. ^{1}H NMR spectrum (600 MHz, CD_3CN, 298 K) of compound **S9**.

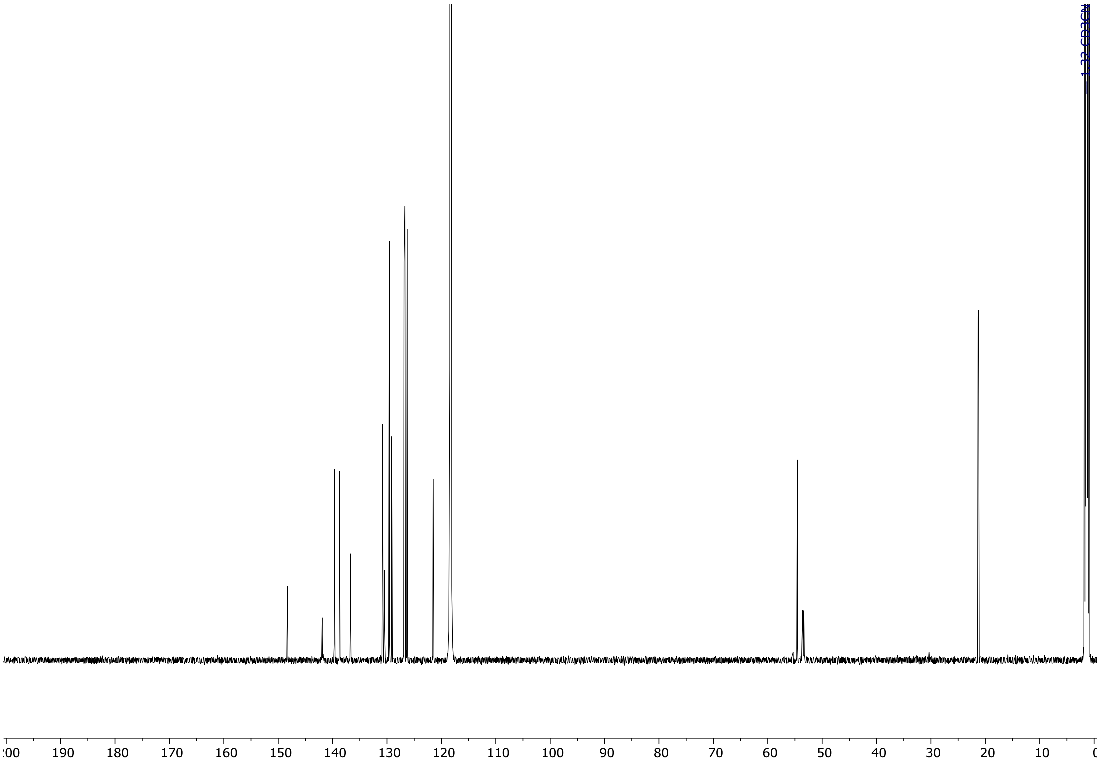

Spectrum S20. ^{13}C NMR spectrum (151 MHz, CD_3CN, 298 K) of compound **S9**.

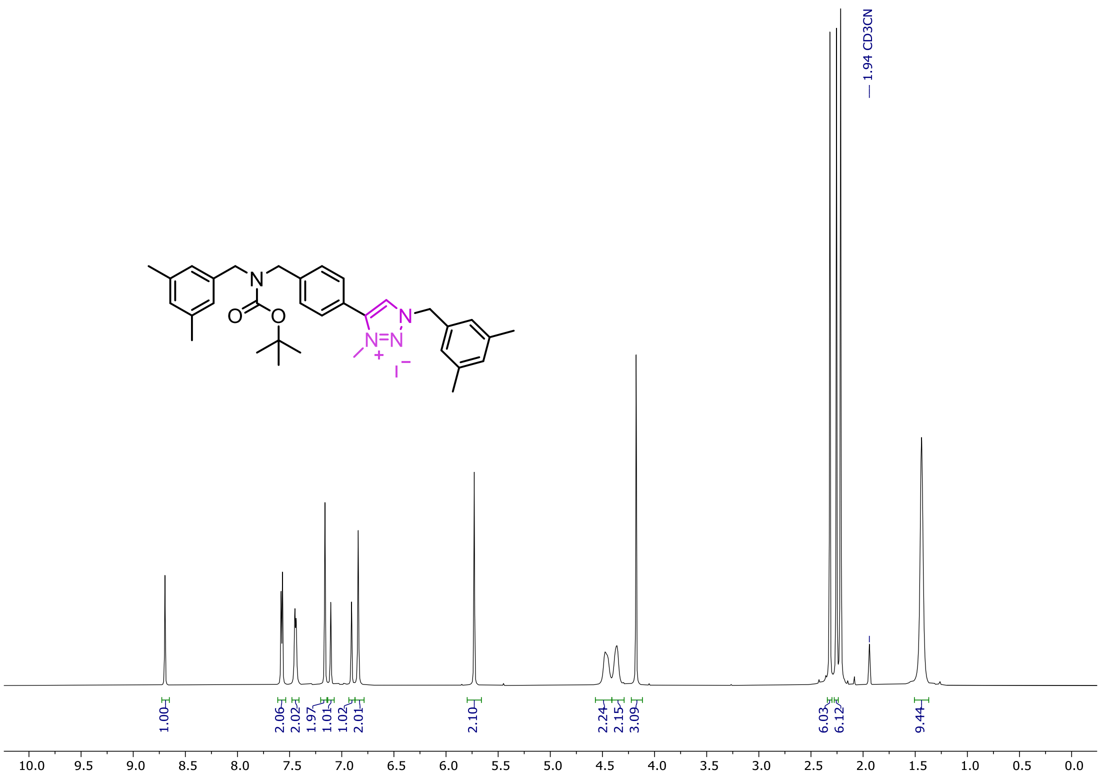

Spectrum S21. ^{1}H NMR spectrum (600 MHz, CD_3CN, 298 K) of compound **S10**.

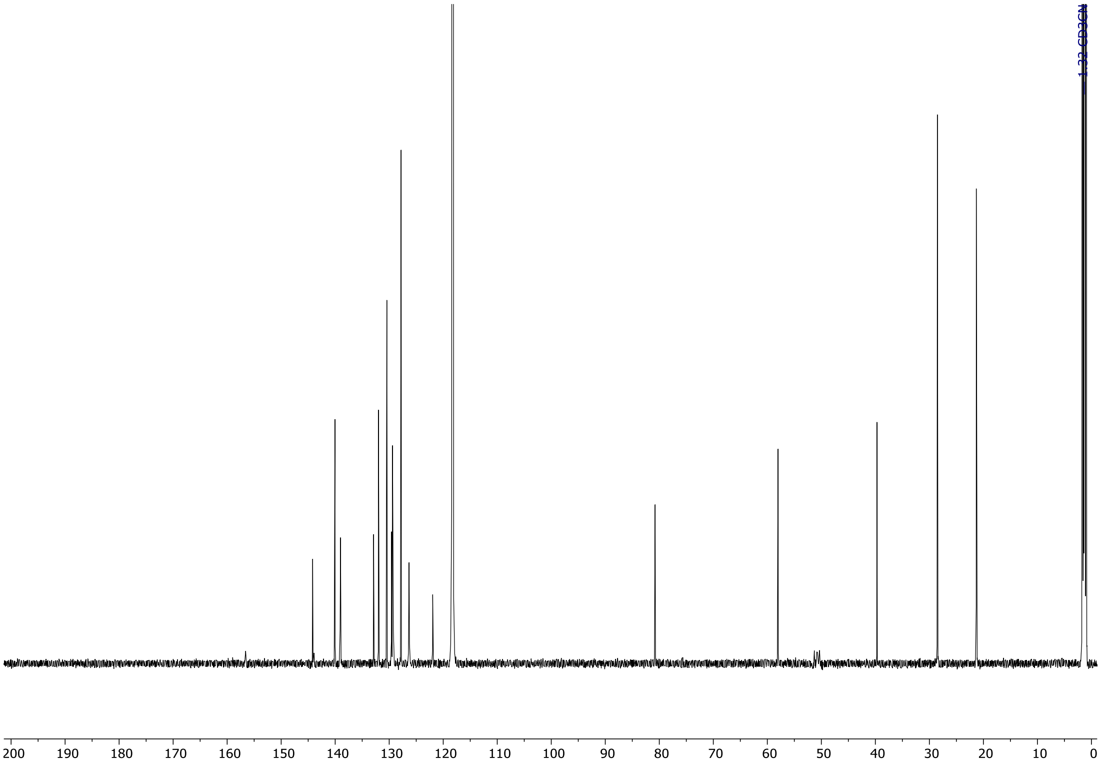

Spectrum S22. ^{13}C NMR spectrum (151 MHz, CD_3CN, 298 K) of compound **S10**.

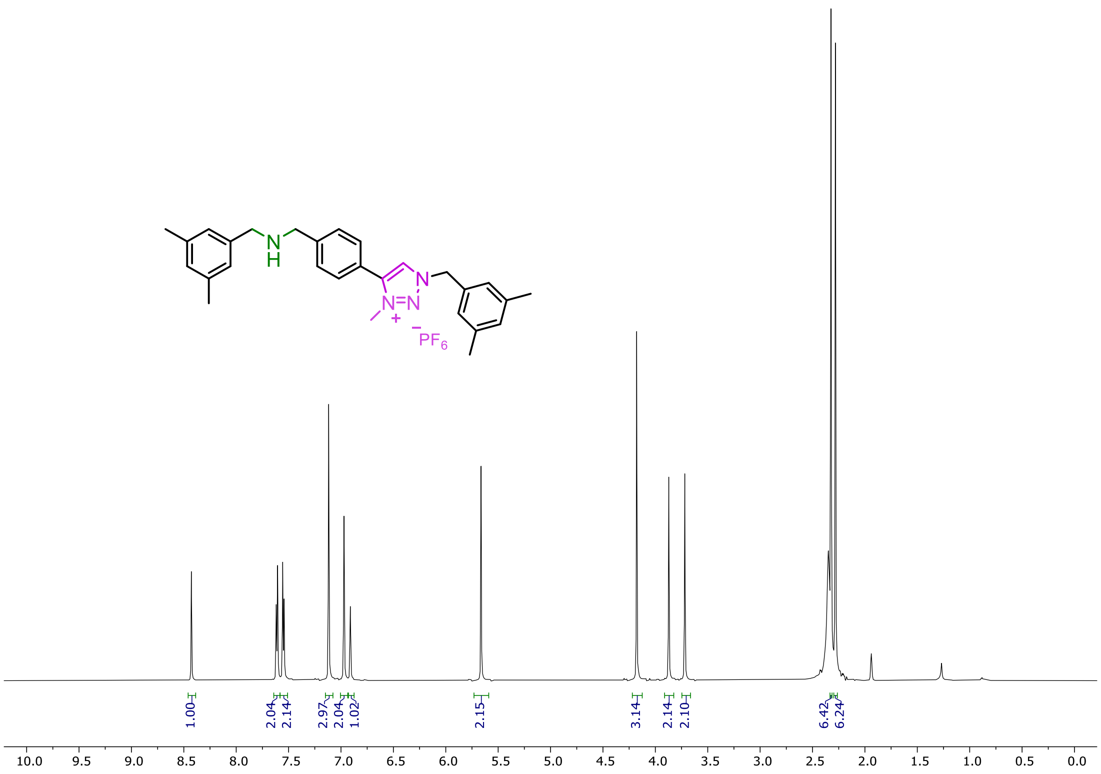

Spectrum S23. ^{1}H NMR spectrum (600 MHz, CD_3CN, 298 K) of compound **S11**.

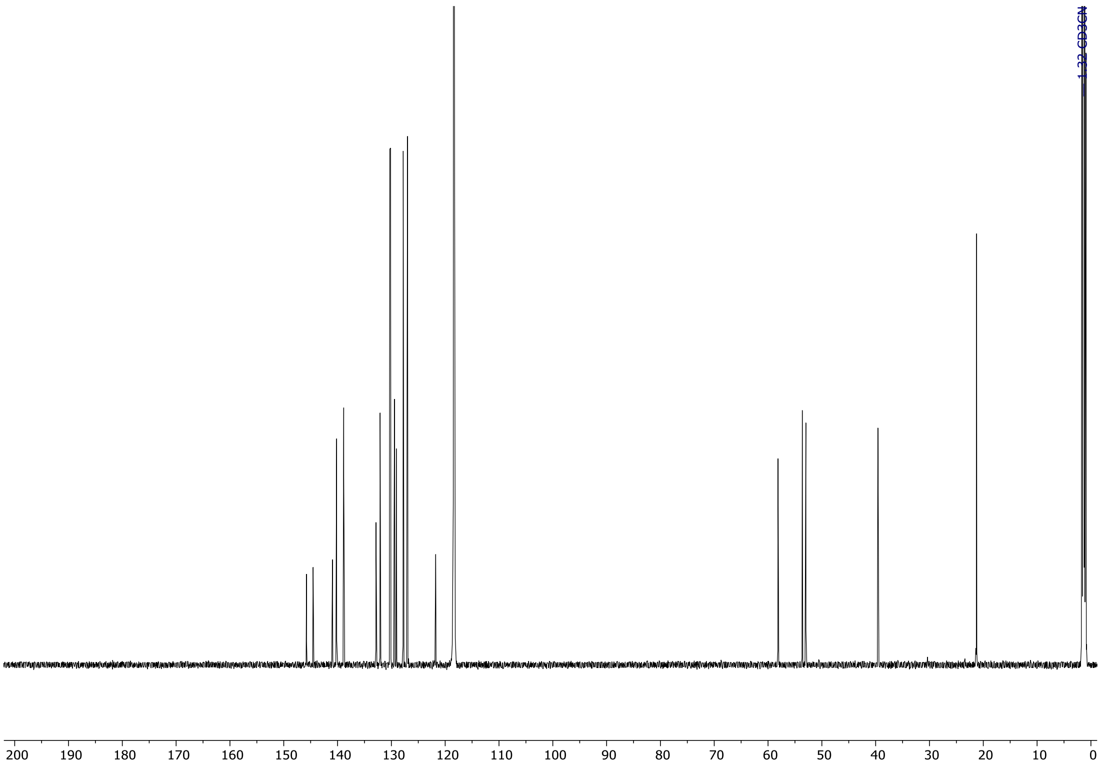

Spectrum S24. ^{13}C NMR spectrum (151 MHz, CD_3CN, 298 K) of compound **S11**.

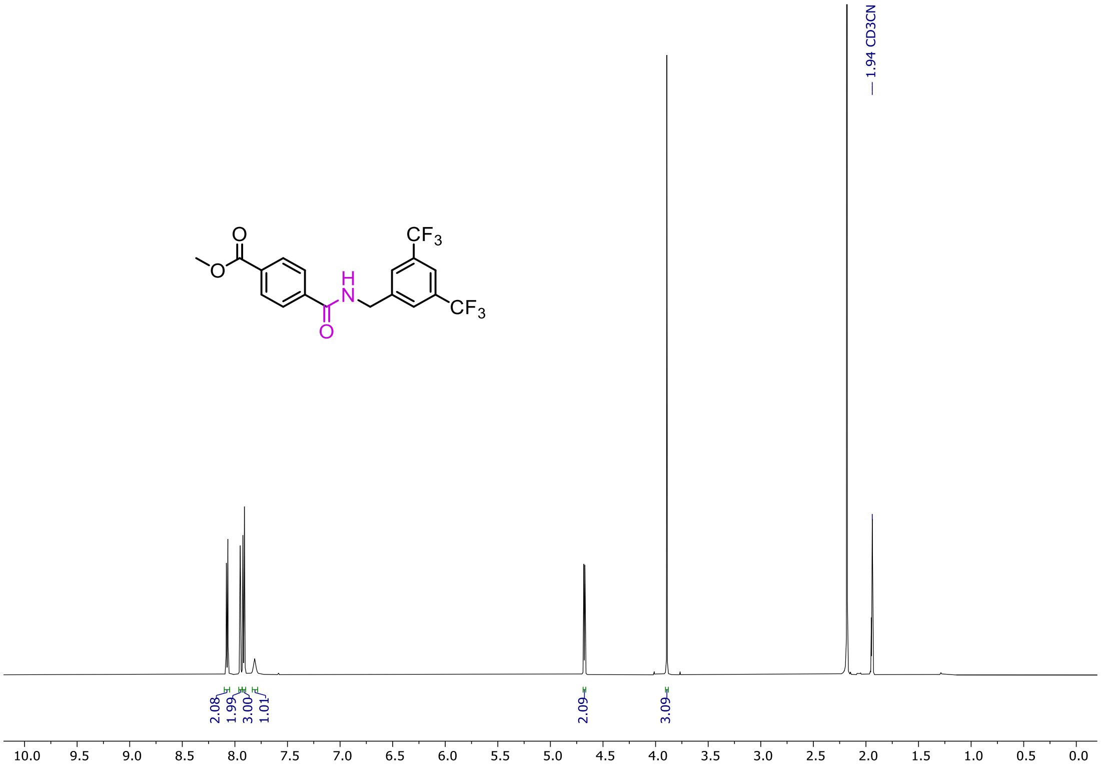

Spectrum S25. ^{1}H NMR spectrum (600 MHz, CD_3CN, 298 K) of compound **S13**.

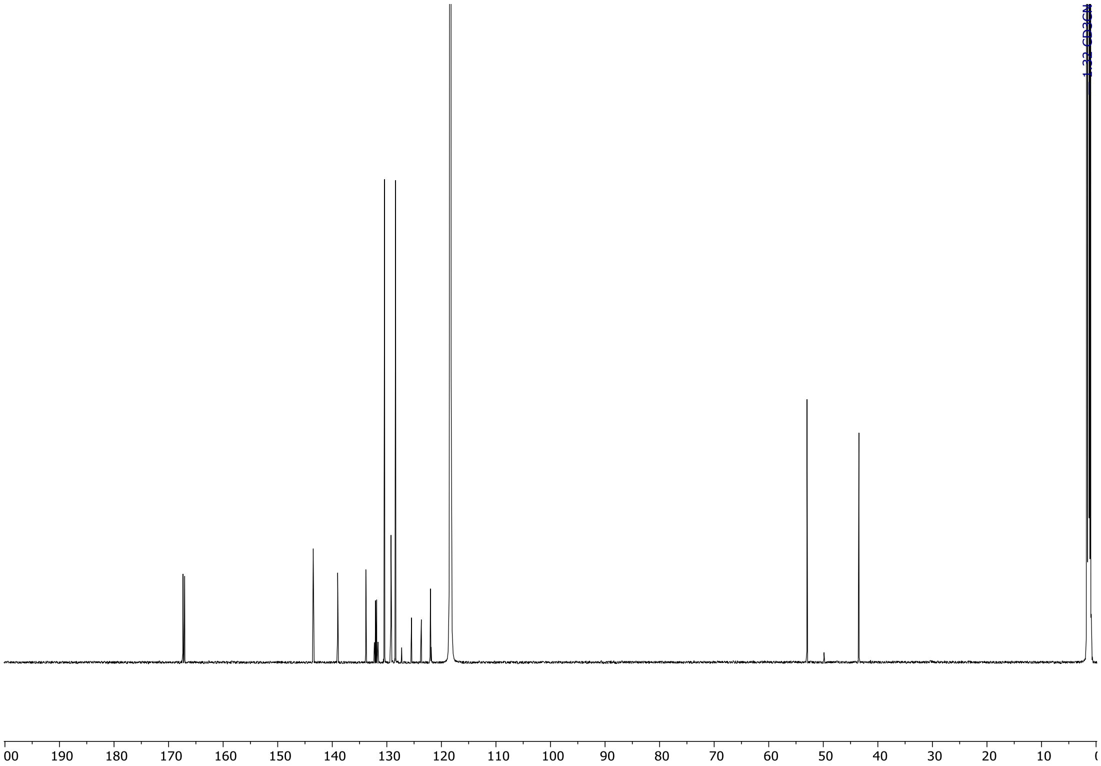

Spectrum S26. ^{13}C NMR spectrum (151 MHz, CD_3CN, 298 K) of compound **S13**.

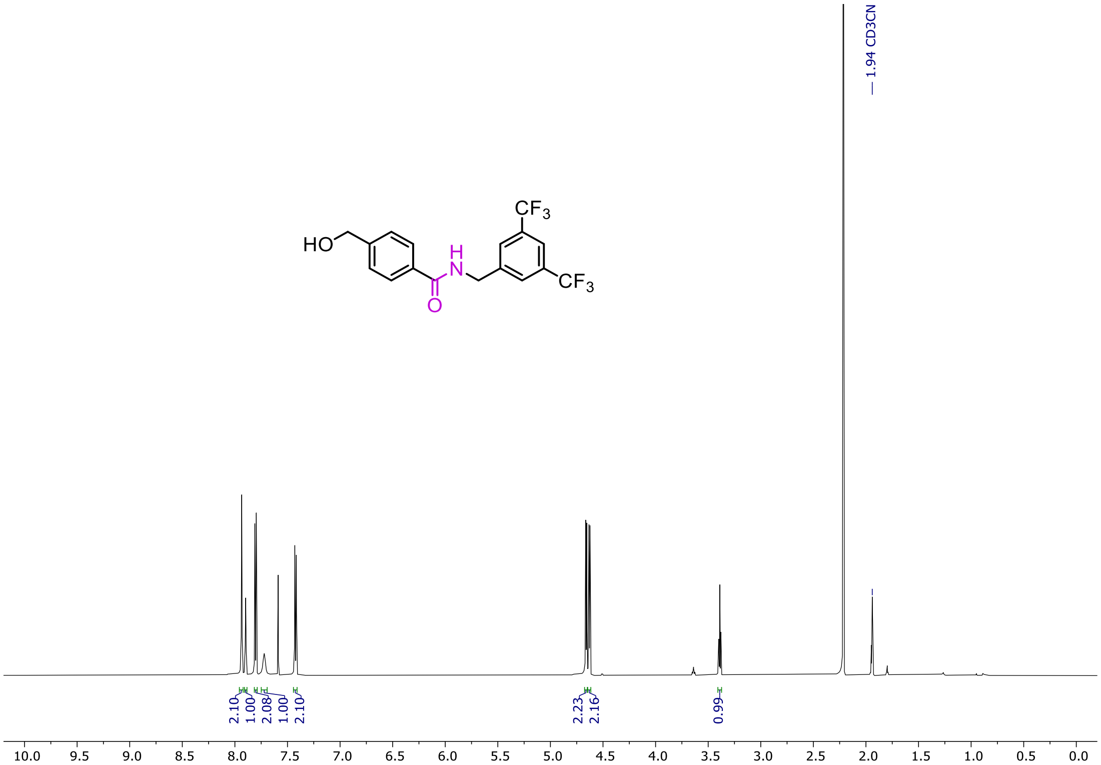

Spectrum S27. ^{1}H NMR spectrum (600 MHz, CD_3CN, 298 K) of compound **S14**.

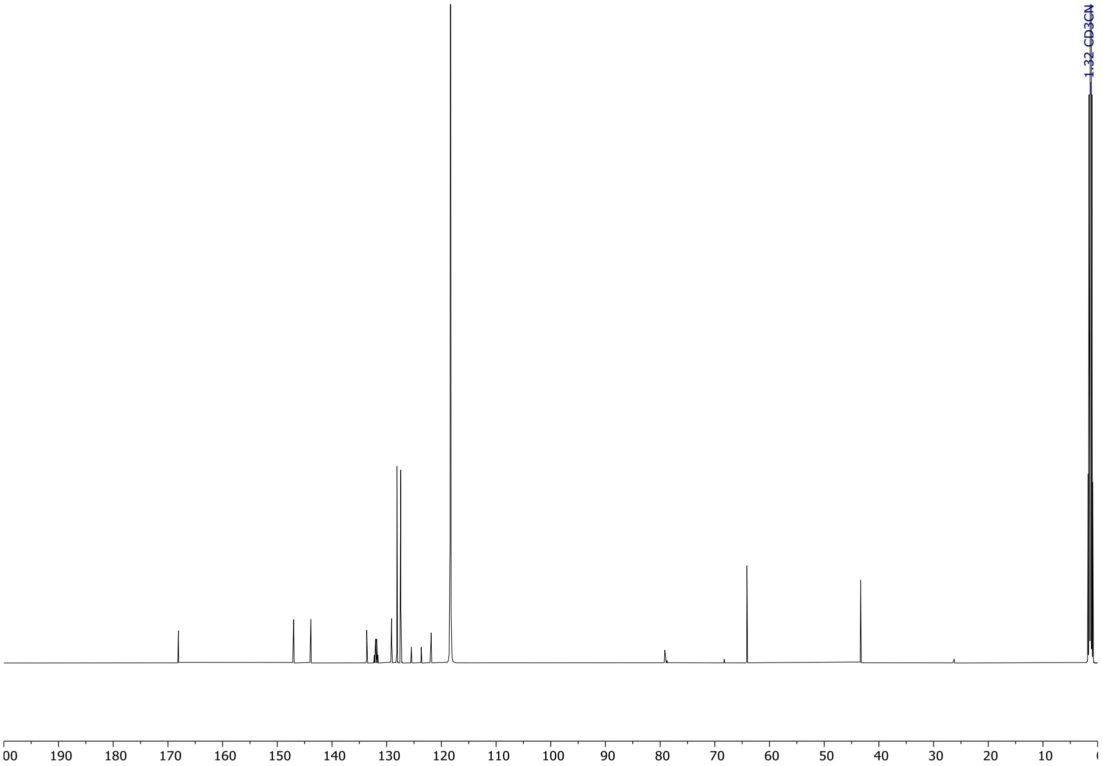

Spectrum S28. ^{13}C NMR spectrum (151 MHz, CD_3CN, 298 K) of compound **S14**.

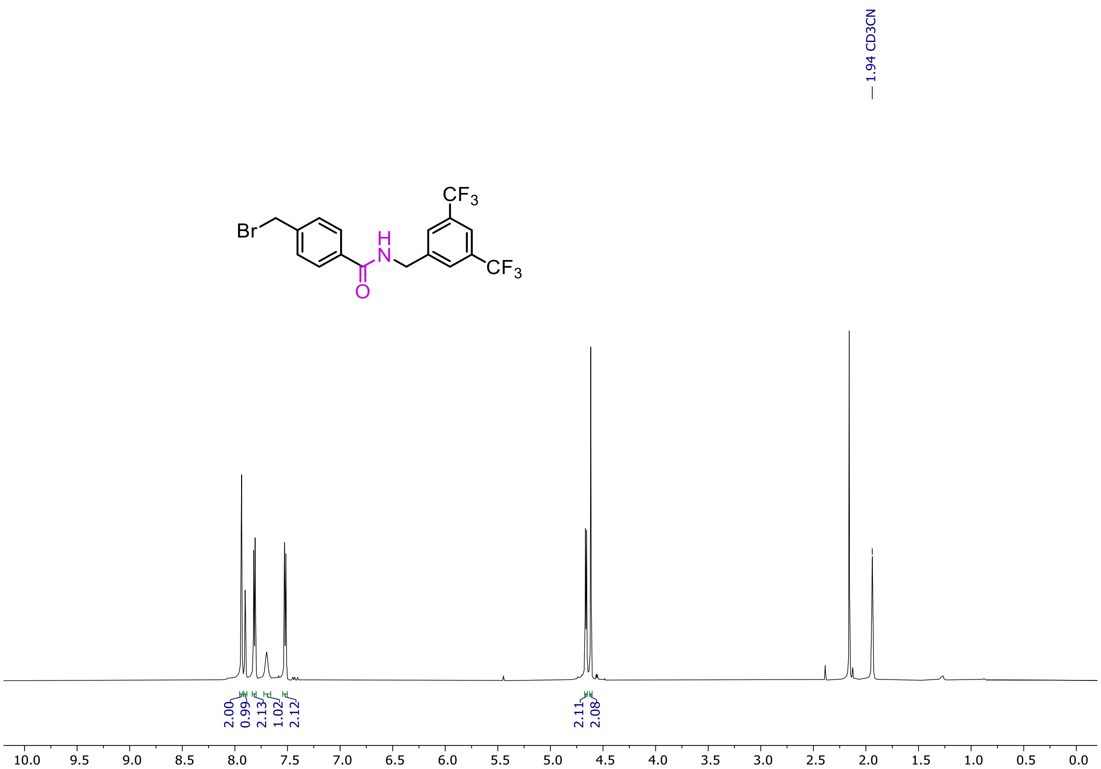

Spectrum S29. ^{1}H NMR spectrum (600 MHz, CD_3CN, 298 K) of compound **S15**.

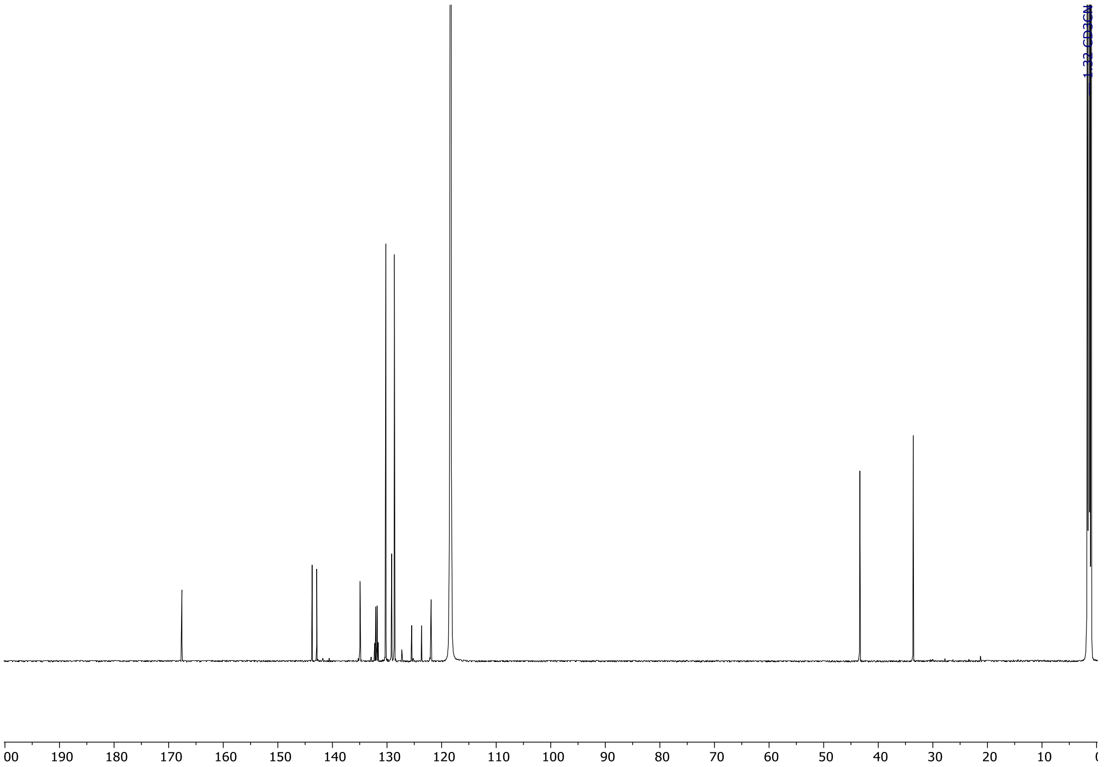

Spectrum S30. ^{13}C NMR spectrum (151 MHz, CD_3CN, 298 K) of compound **S15**.

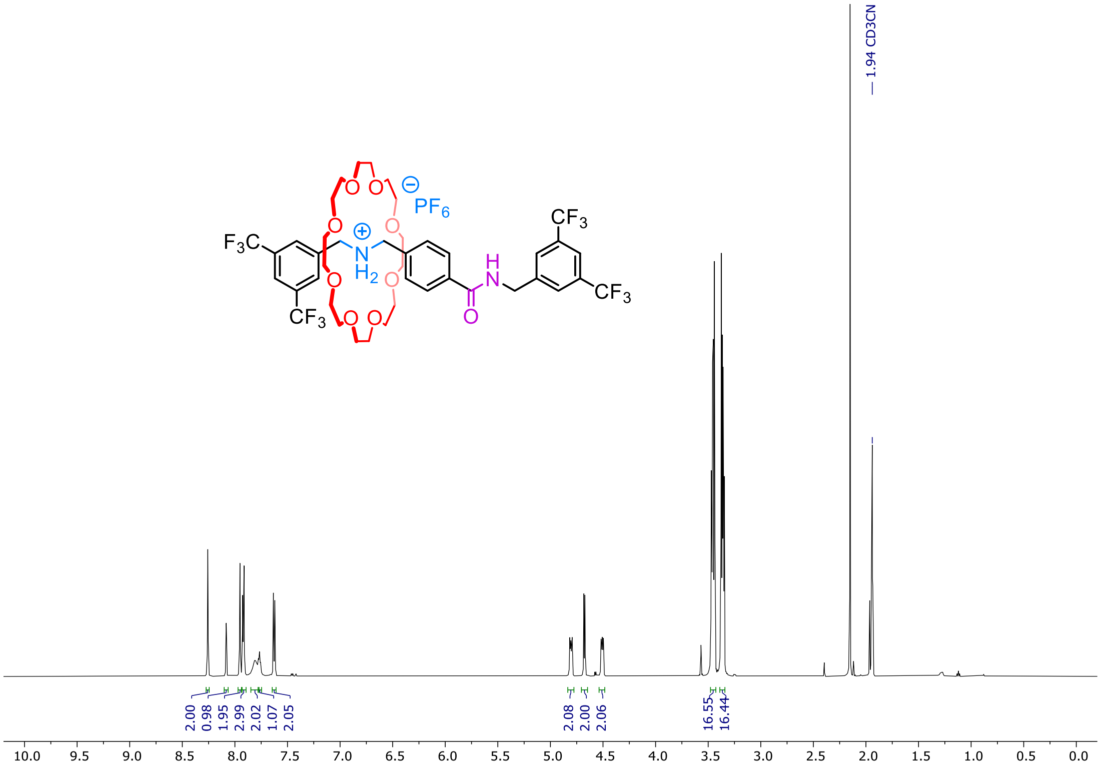

Spectrum S31. ^{1}H NMR spectrum (600 MHz, CD_3CN, 298 K) of compound **4**·HPF_6.

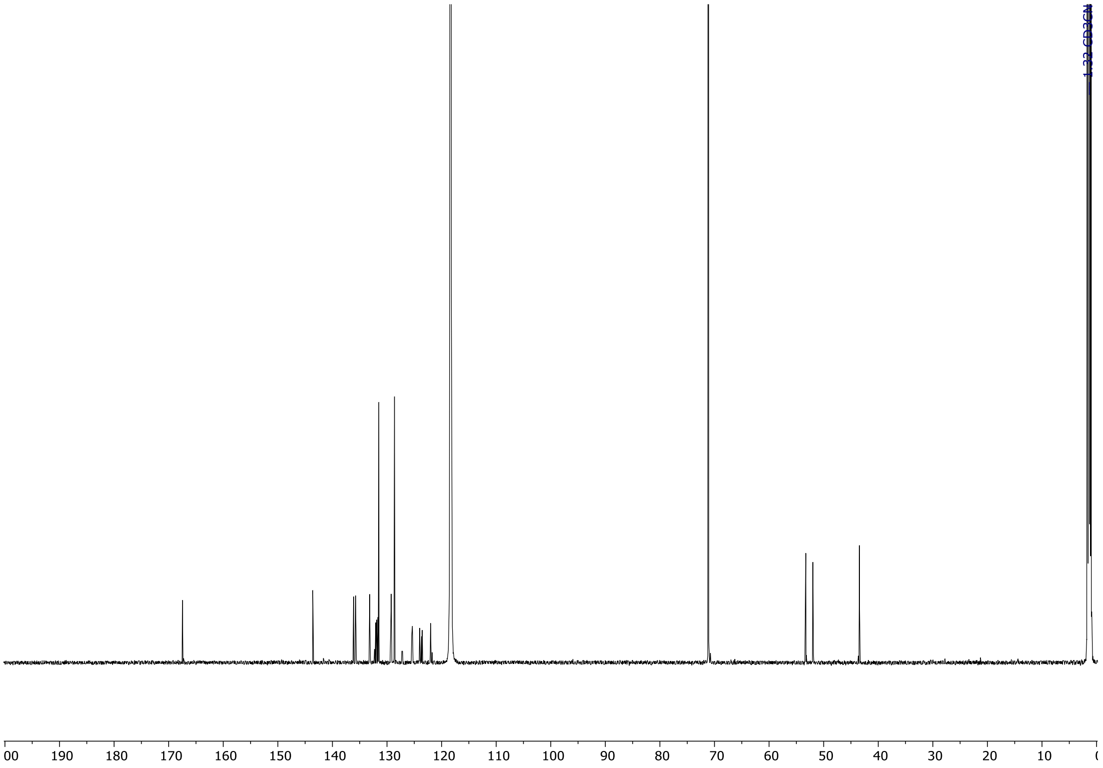

Spectrum S32. ^{13}C NMR spectrum (151 MHz, CD_3CN, 298 K) of compound **4**·HPF_6.

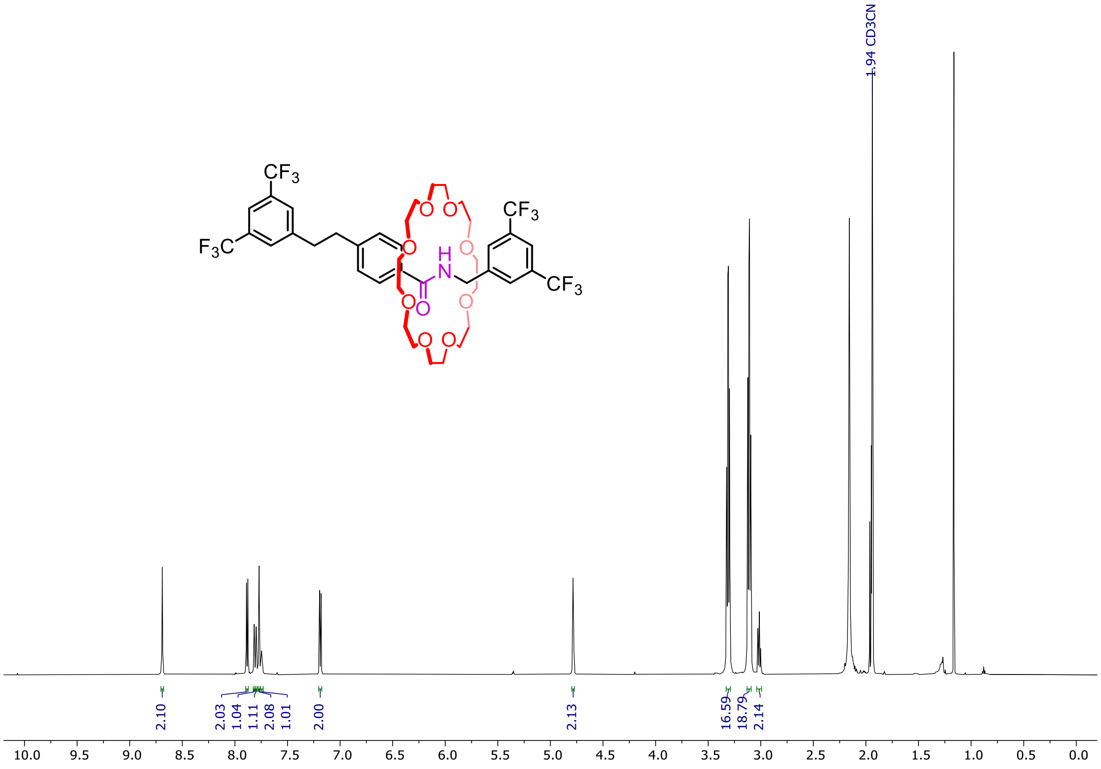

Spectrum S33. ^{1}H NMR spectrum (600 MHz, CD_3CN, 298 K) of compound **8**.

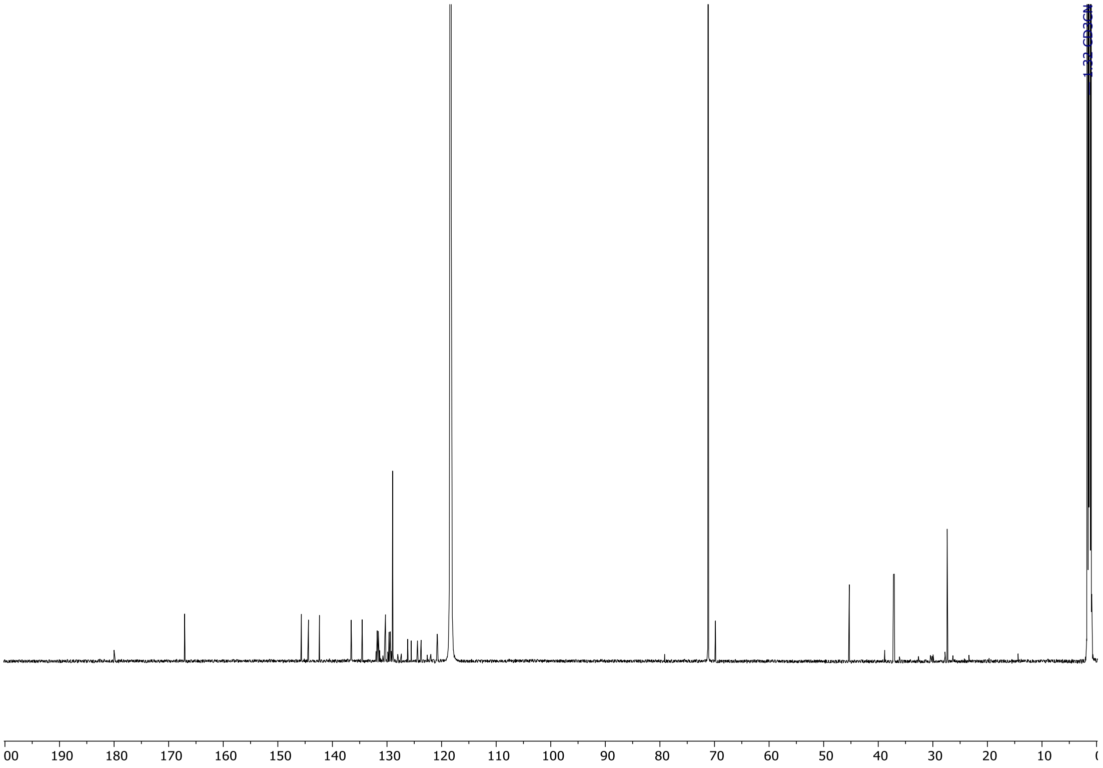

Spectrum S34. ^{13}C NMR spectrum (151 MHz, CD_3CN, 298 K) of compound **8**.

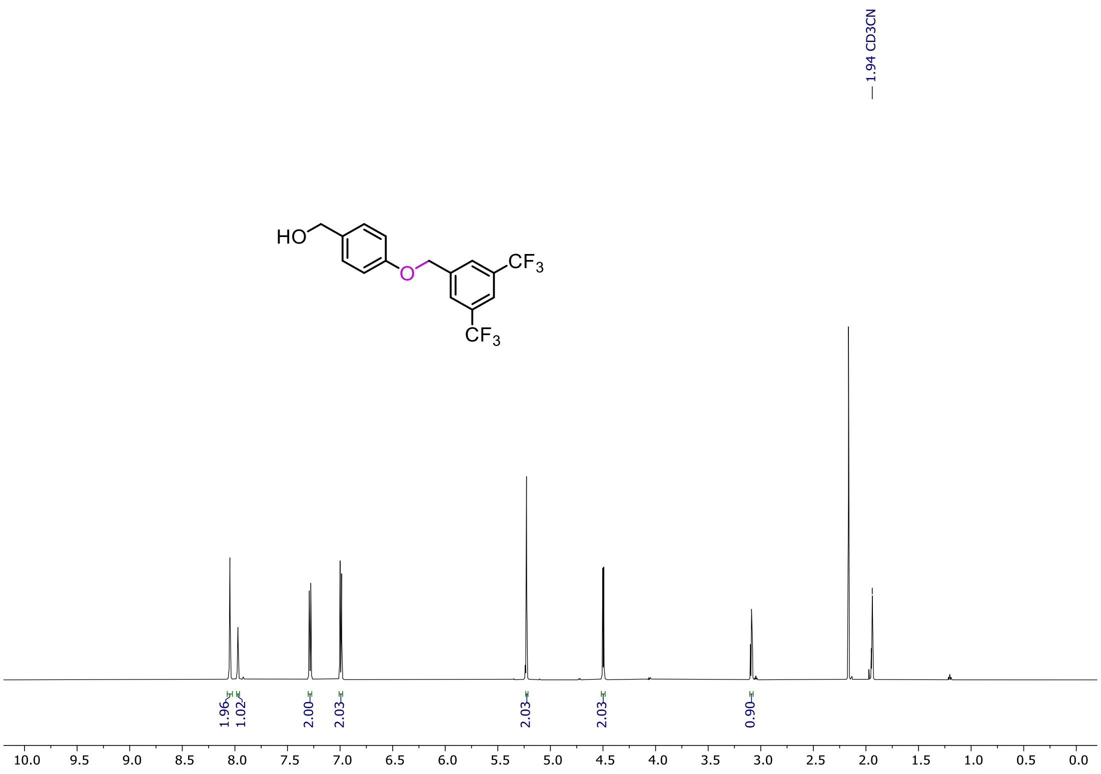

Spectrum S35. ^{1}H NMR spectrum (600 MHz, CD_3CN, 298 K) of compound **S18**.

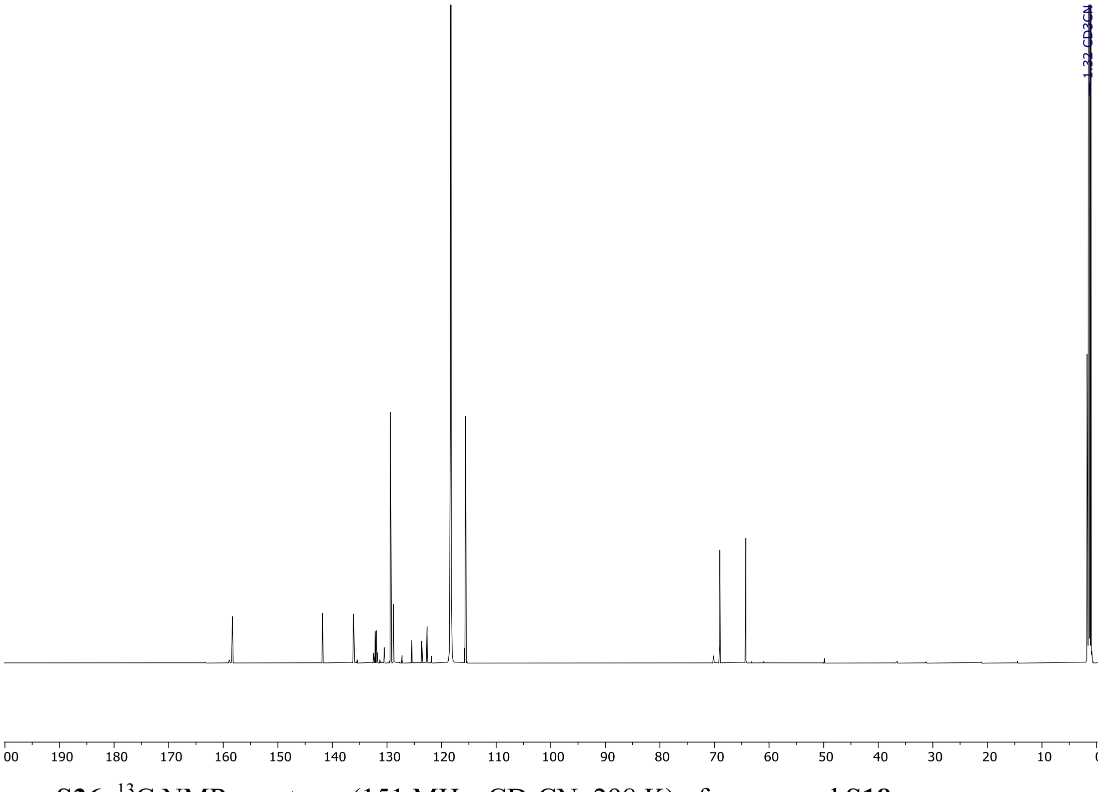

Spectrum S36. ^{13}C NMR spectrum (151 MHz, CD_3CN, 298 K) of compound **S18**.

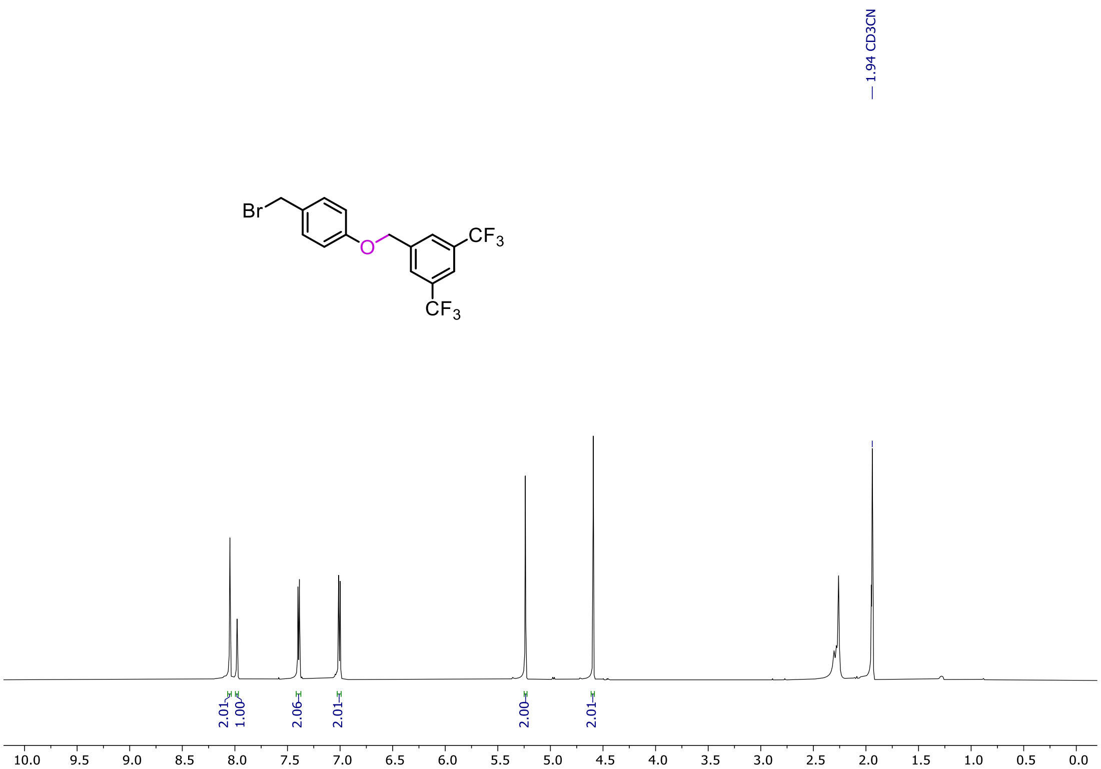

Spectrum S37. ^{1}H NMR spectrum (600 MHz, CD_3CN, 298 K) of compound **S19**.

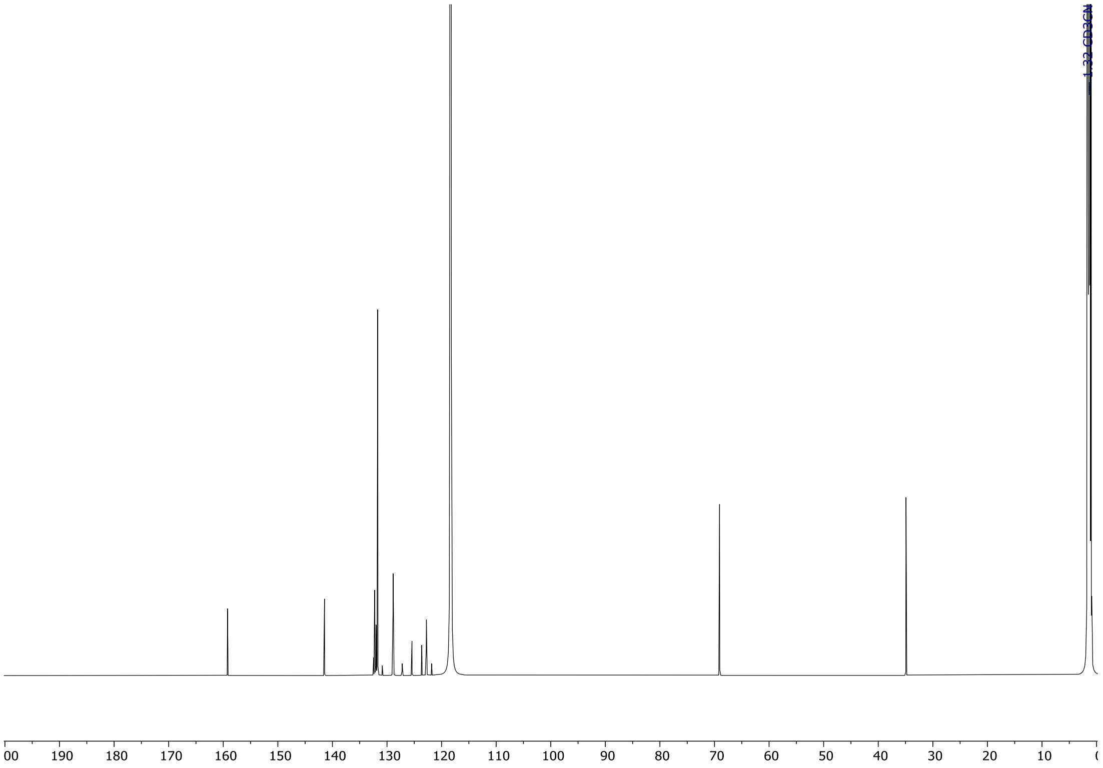

Spectrum S38. ^{13}C NMR spectrum (151 MHz, CD_3CN, 298 K) of compound **S19**.

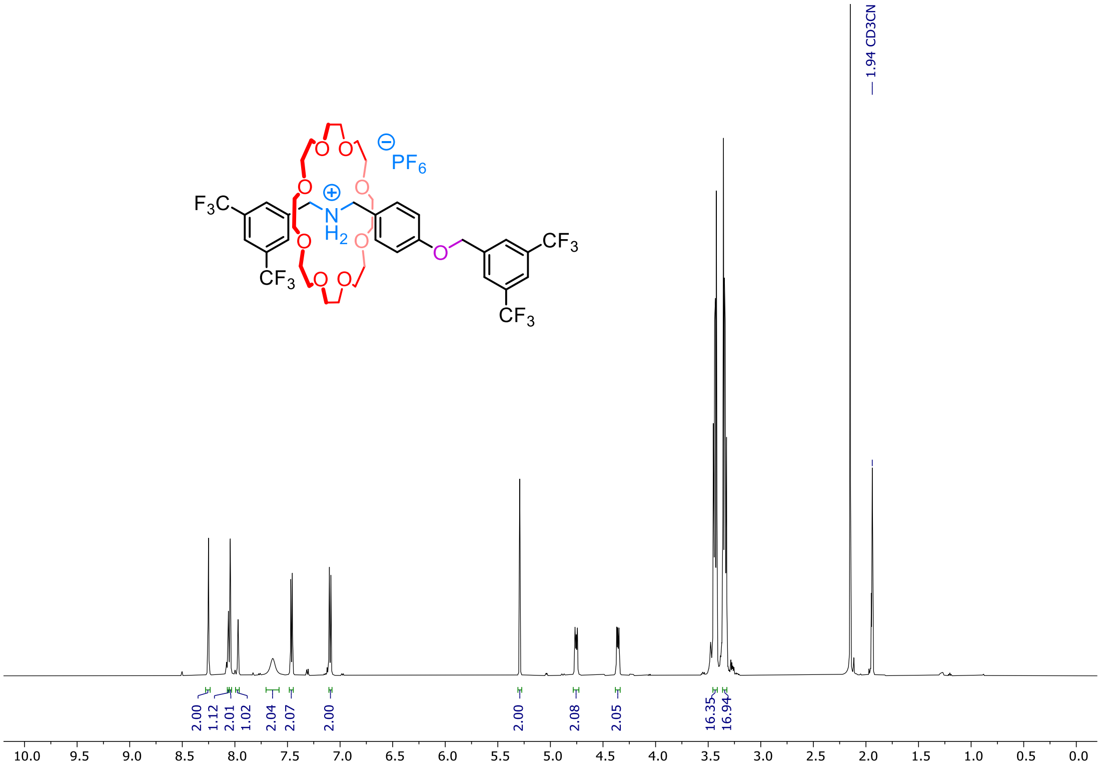

Spectrum S39. ^{1}H NMR spectrum (600 MHz, CD_3CN, 298 K) of compound **5**·HPF_6.

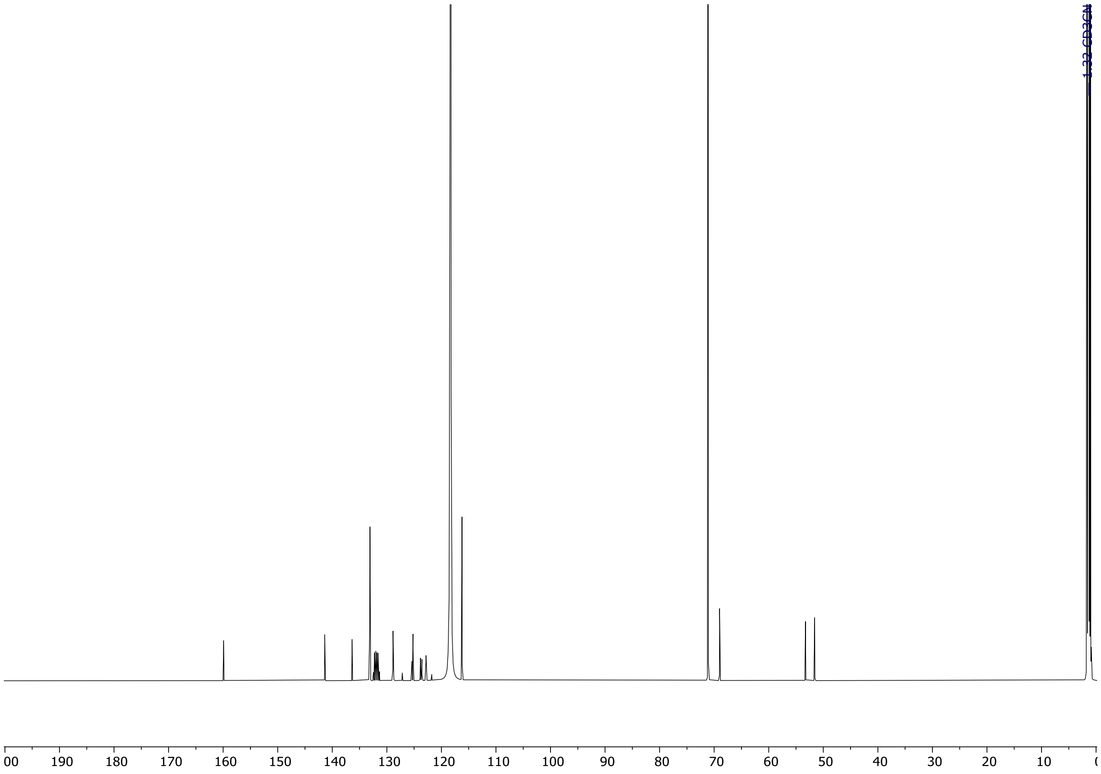

Spectrum S40. ^{13}C NMR spectrum (151 MHz, CD_3CN, 298 K) of compound **5**·HPF_6.

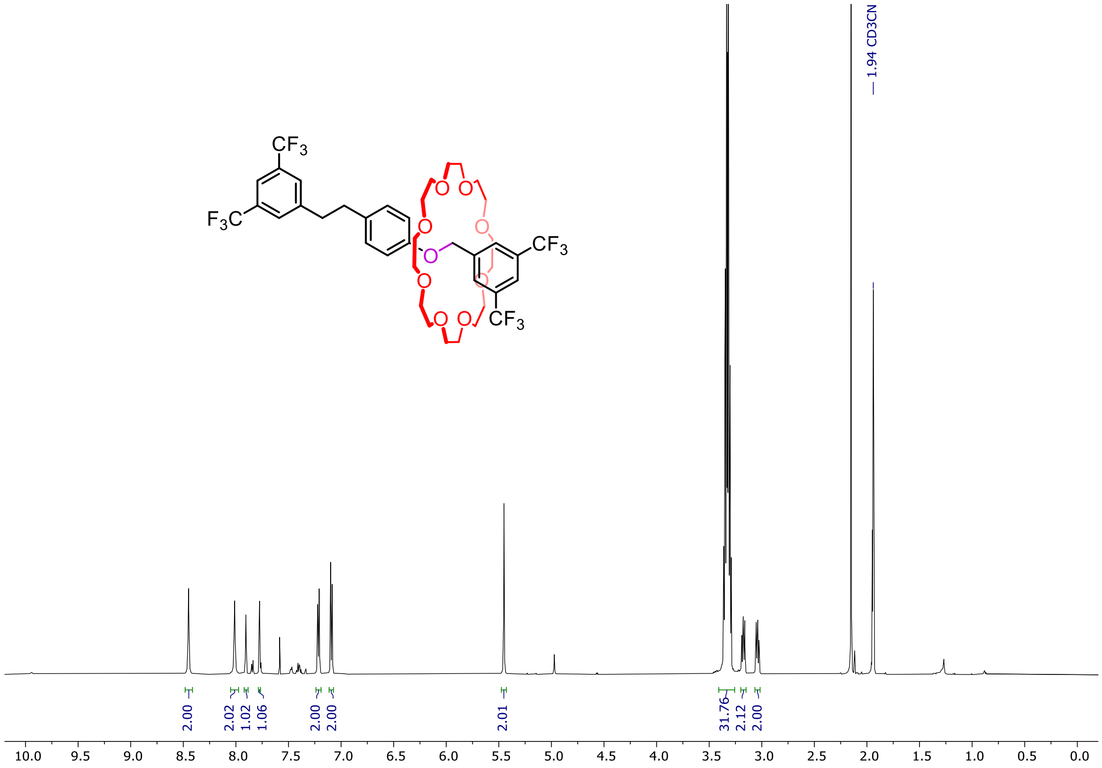

Spectrum S41. ^{1}H NMR spectrum (600 MHz, CD_3CN, 298 K) of compound **9**.

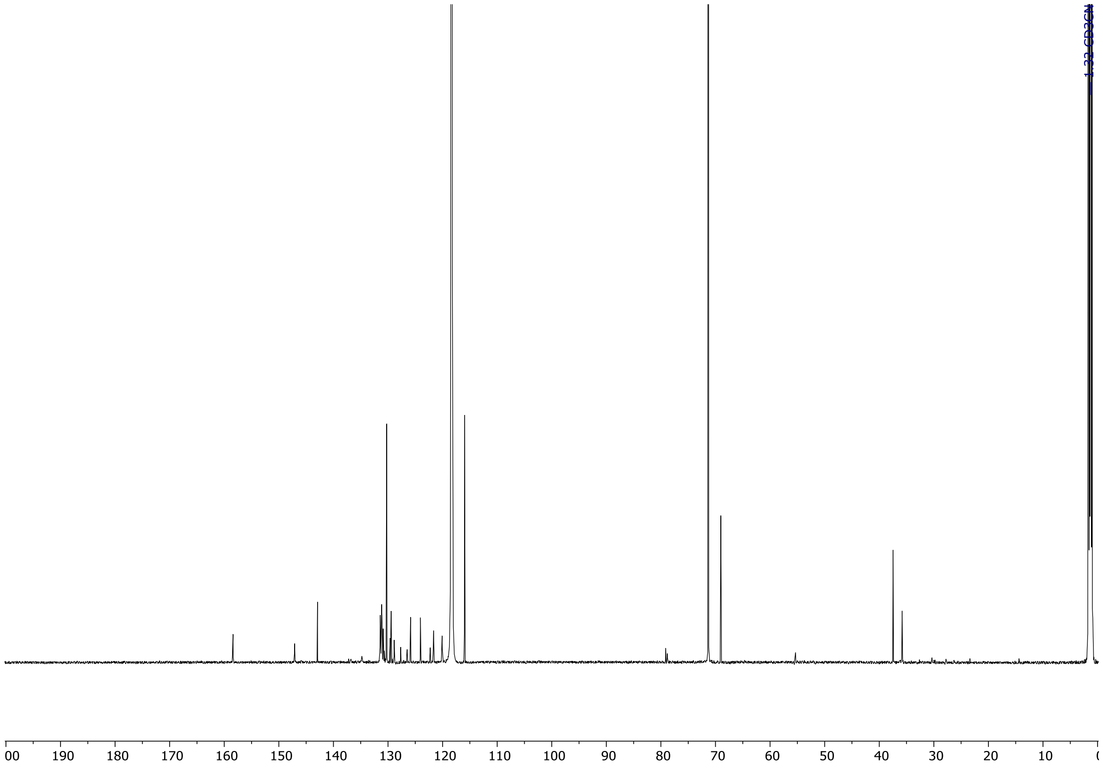

Spectrum S42. ^{13}C NMR spectrum (151 MHz, CD_3CN, 298 K) of compound **9**.

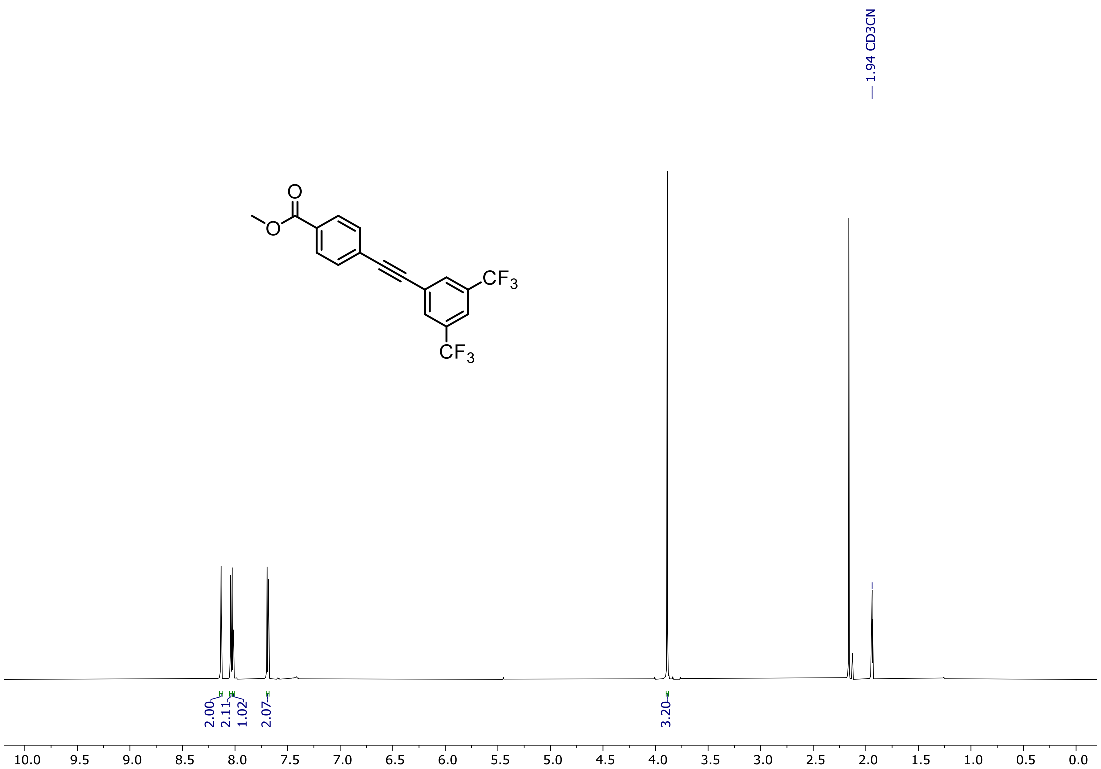

Spectrum S43. ^{1}H NMR spectrum (600 MHz, CD_3CN, 298 K) of compound **S22**.

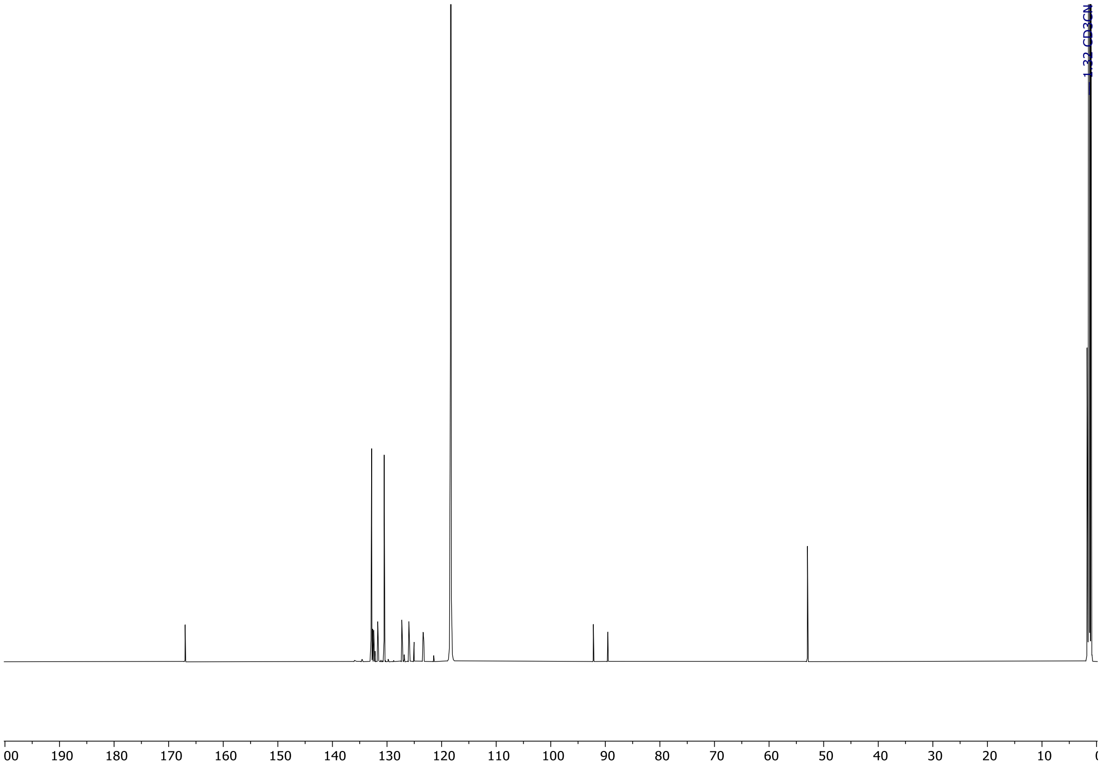

Spectrum S44. ^{13}C NMR spectrum (151 MHz, CD_3CN, 298 K) of compound **S22**.

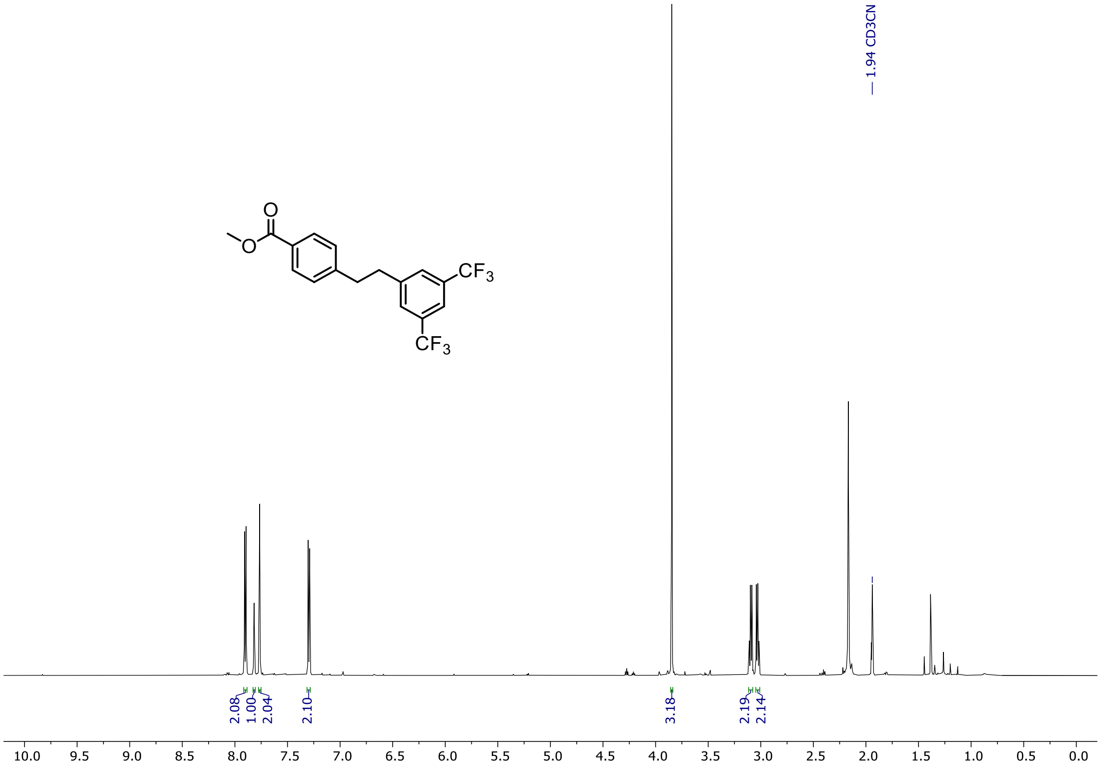

Spectrum S45. ^{1}H NMR spectrum (600 MHz, CD_3CN, 298 K) of compound **S23**.

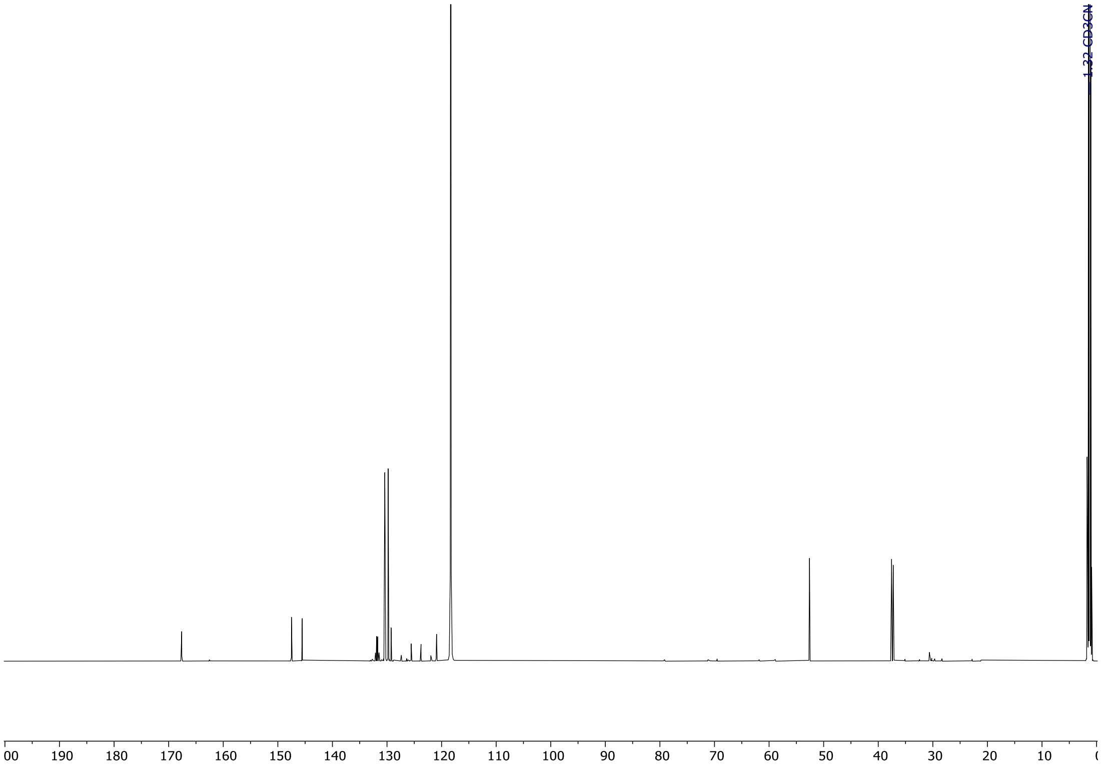

Spectrum S46. ^{13}C NMR spectrum (151 MHz, CD_3CN, 298 K) of compound **S23**.

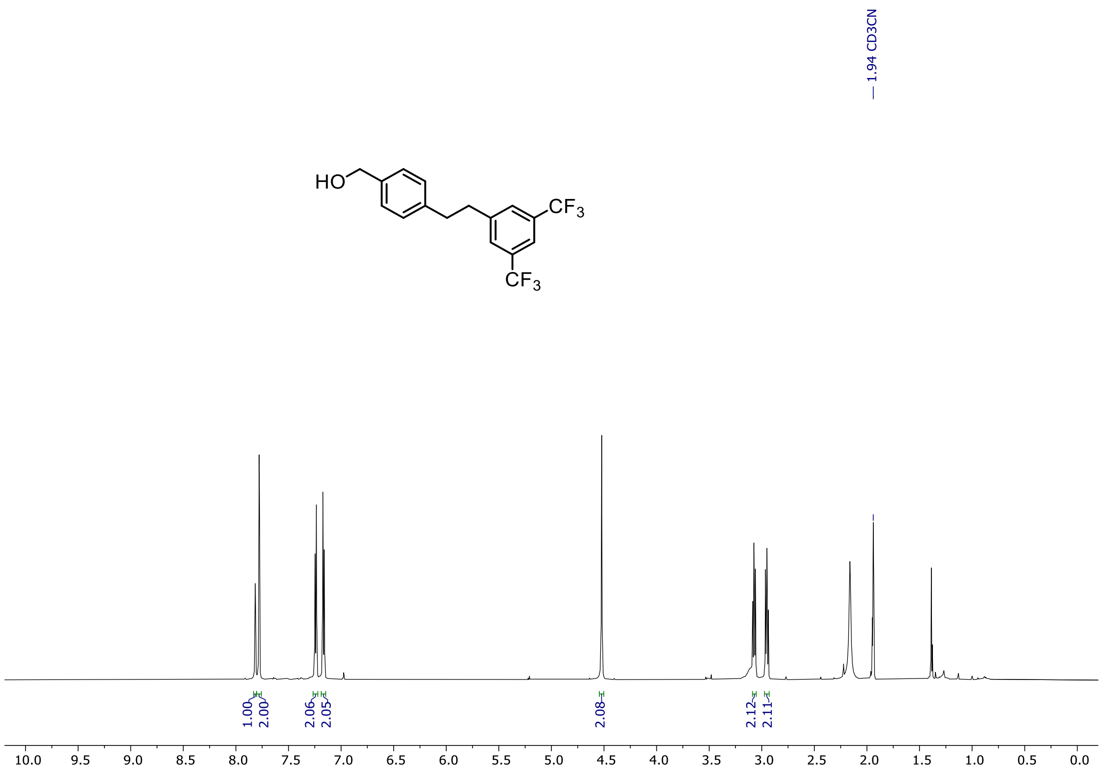

Spectrum S47. ^{1}H NMR spectrum (600 MHz, CD_3CN, 298 K) of compound **S24**.

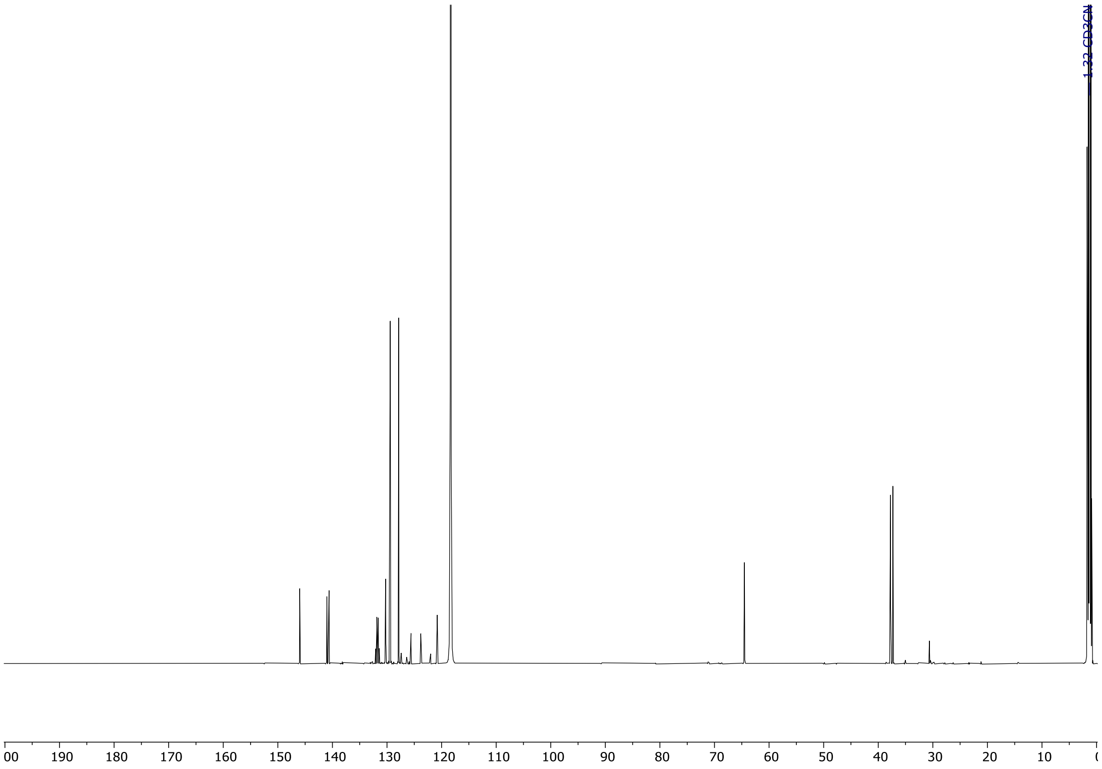

Spectrum S48. ^{13}C NMR spectrum (151 MHz, CD_3CN, 298 K) of compound **S24**.

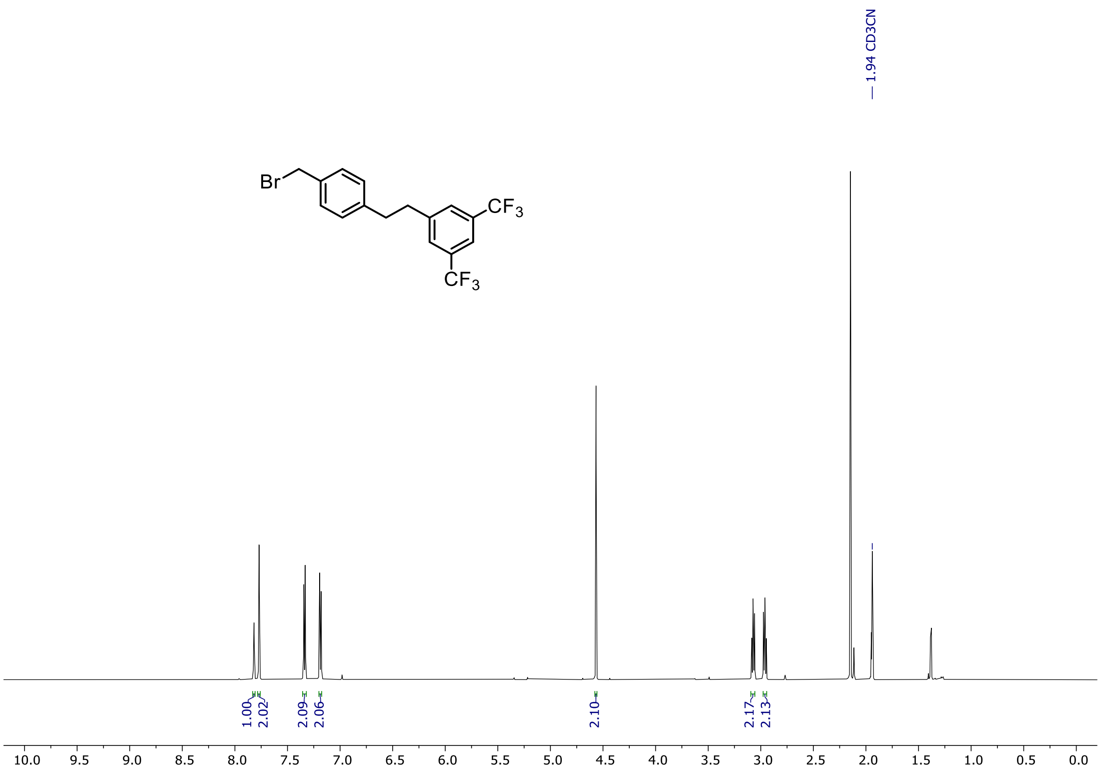

Spectrum S49. ^{1}H NMR spectrum (600 MHz, CD_3CN, 298 K) of compound **16**.

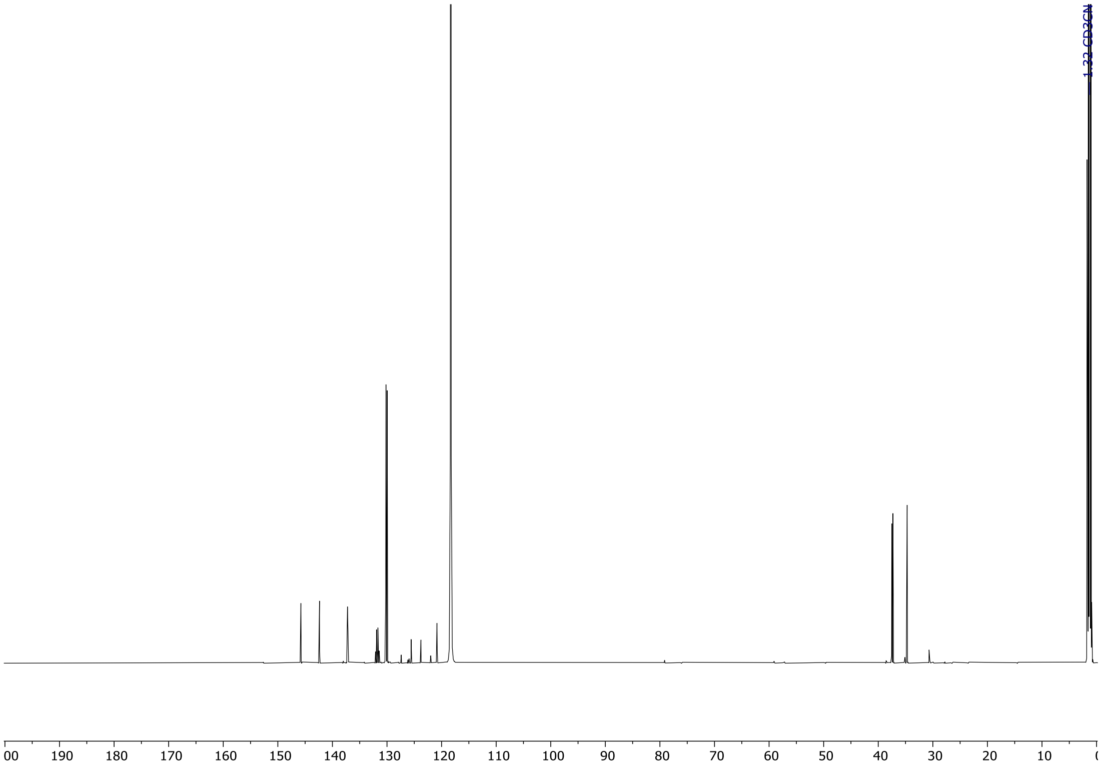

Spectrum S50. ^{13}C NMR spectrum (151 MHz, CD_3CN, 298 K) of compound **16**.

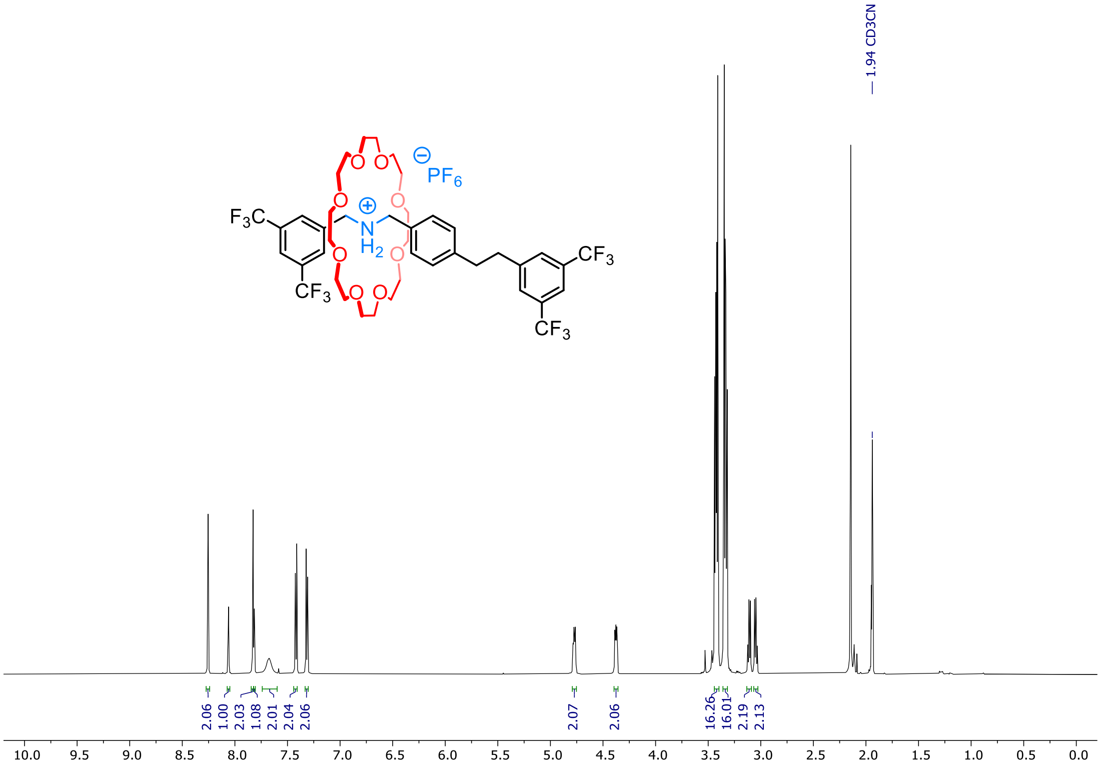

Spectrum S51. ^{1}H NMR spectrum (600 MHz, CD_3CN, 298 K) of compound **10**·HPF_6.

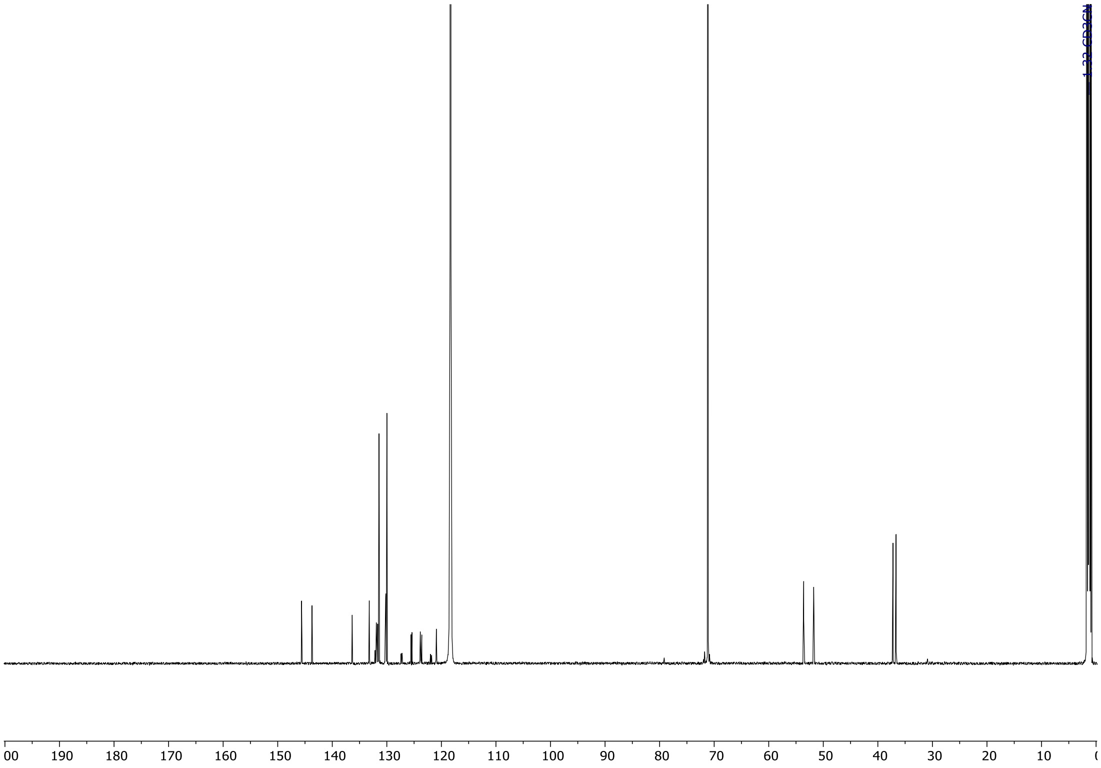

Spectrum S52. ^{13}C NMR spectrum (151 MHz, CD_3CN, 298 K) of compound **10**·HPF_6.

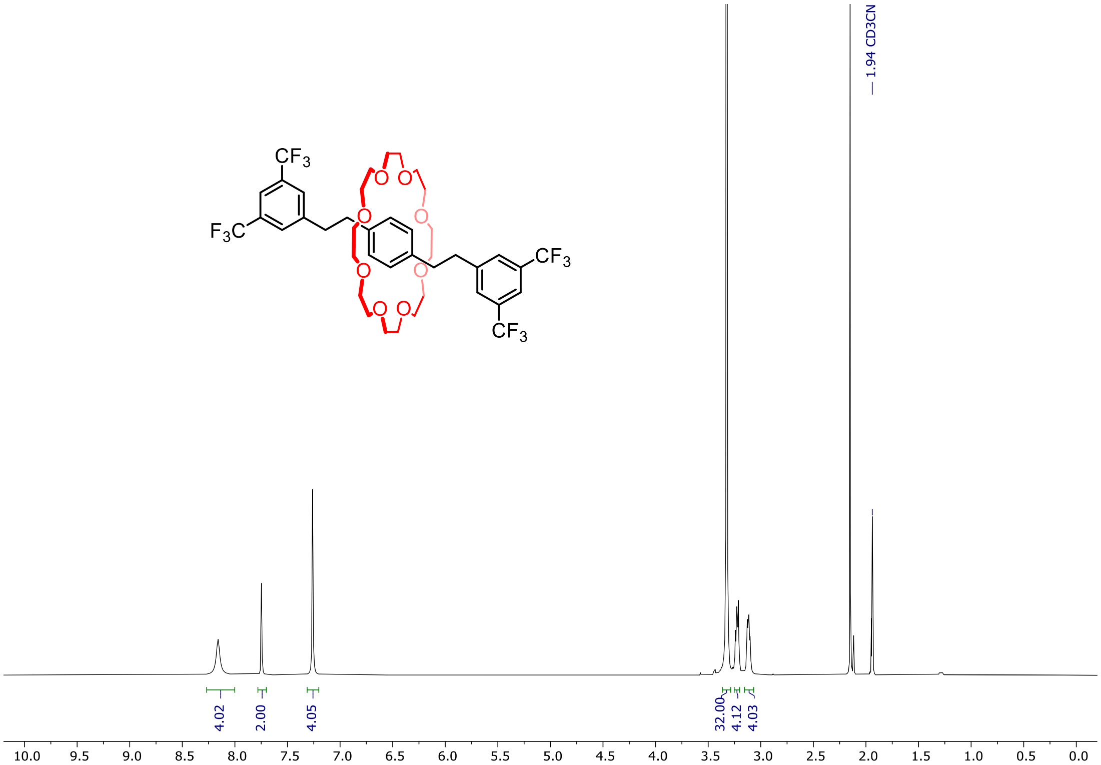

Spectrum S53. ^{1}H NMR spectrum (600 MHz, CD_3CN, 298 K) of compound **17**.

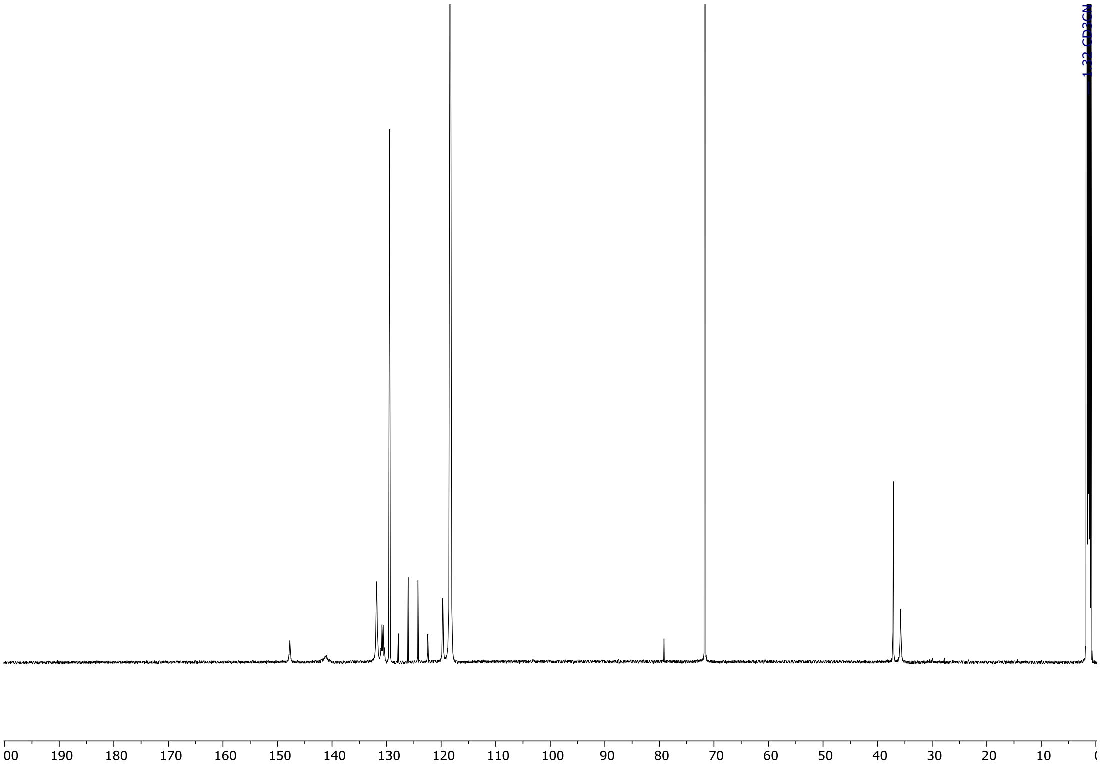

Spectrum S54. ^{13}C NMR spectrum (151 MHz, CD_3CN, 298 K) of compound **17**.

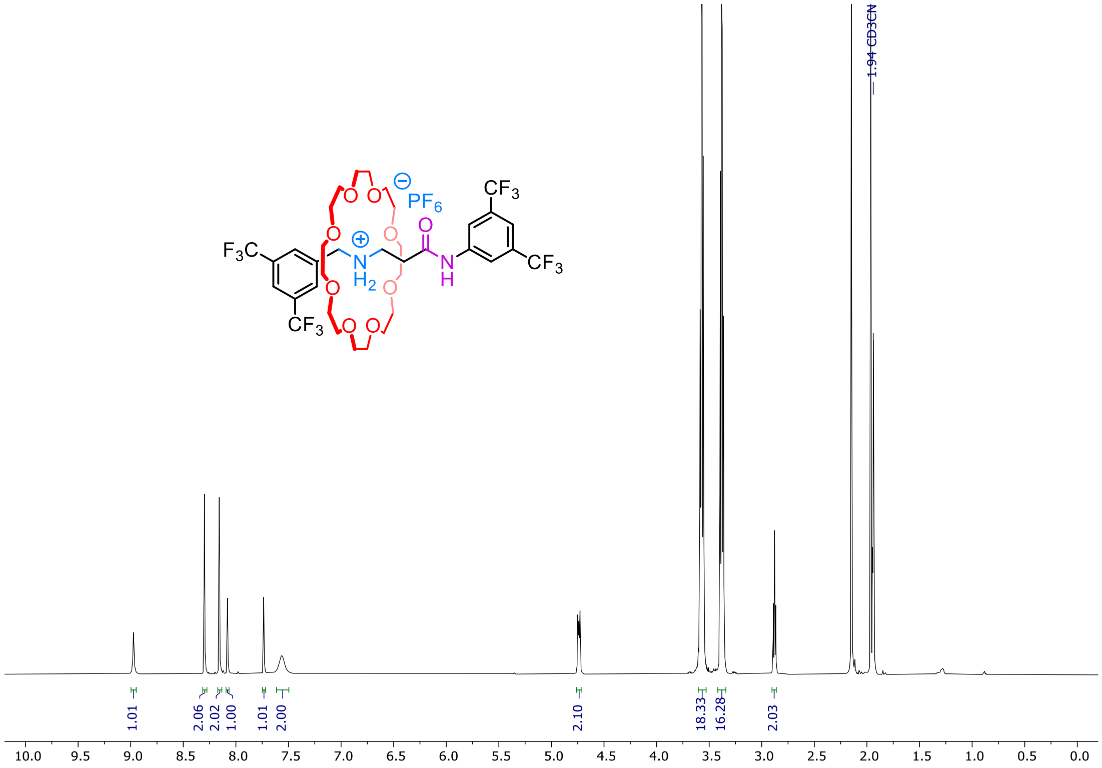

Spectrum S55. ^{1}H NMR spectrum (600 MHz, CD_3CN, 298 K) of compound **11**·PF_6.

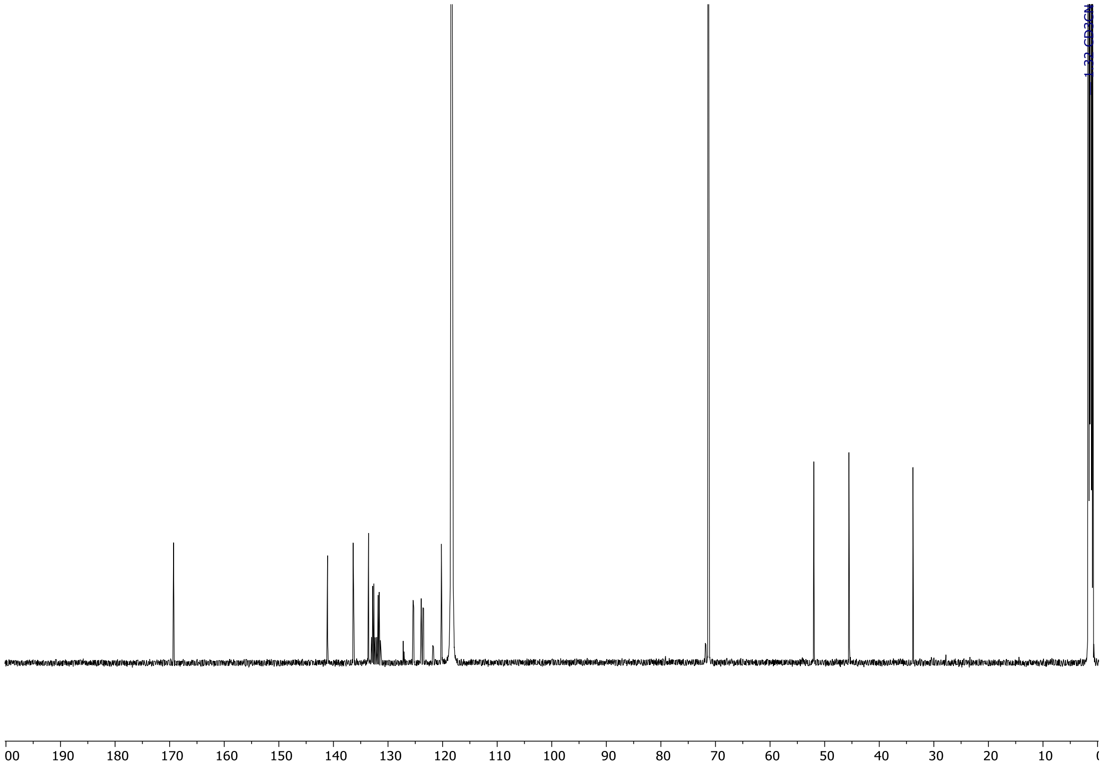

Spectrum S56. ^{13}C NMR spectrum (151 MHz, CD_3CN, 298 K) of compound **11**·PF_6.

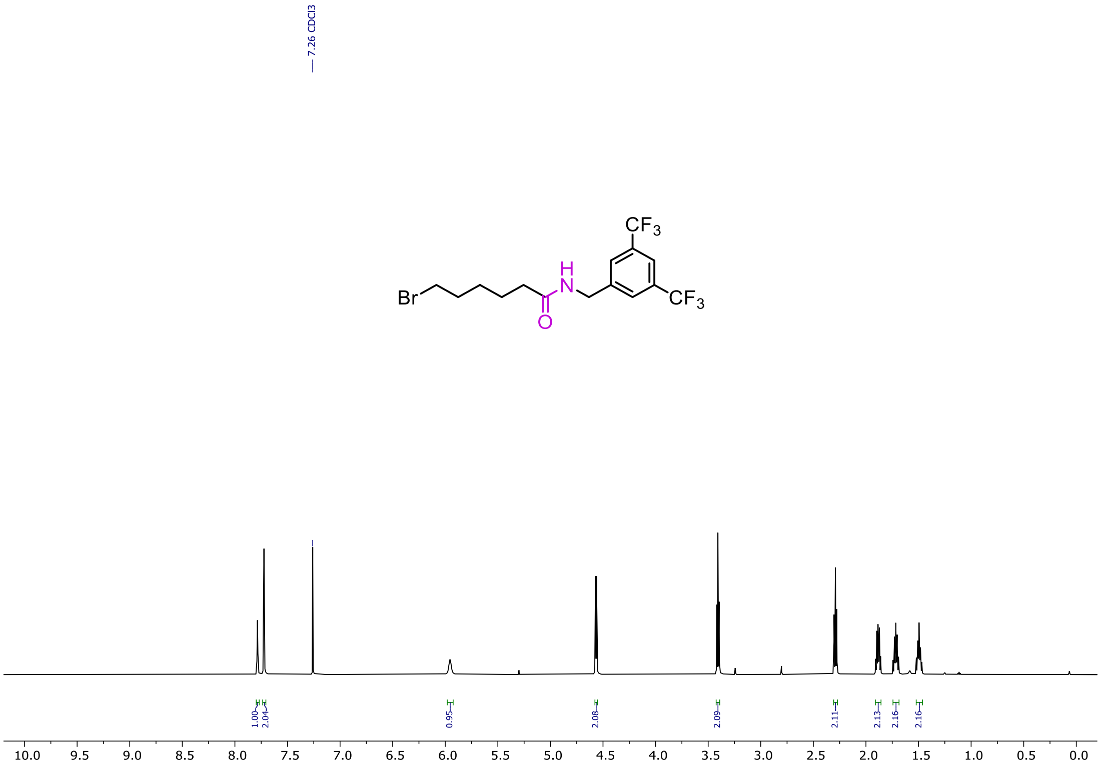

Spectrum S57. ^{1}H NMR spectrum (600 MHz, $CDCl_3$, 298 K) of compound **S29**.

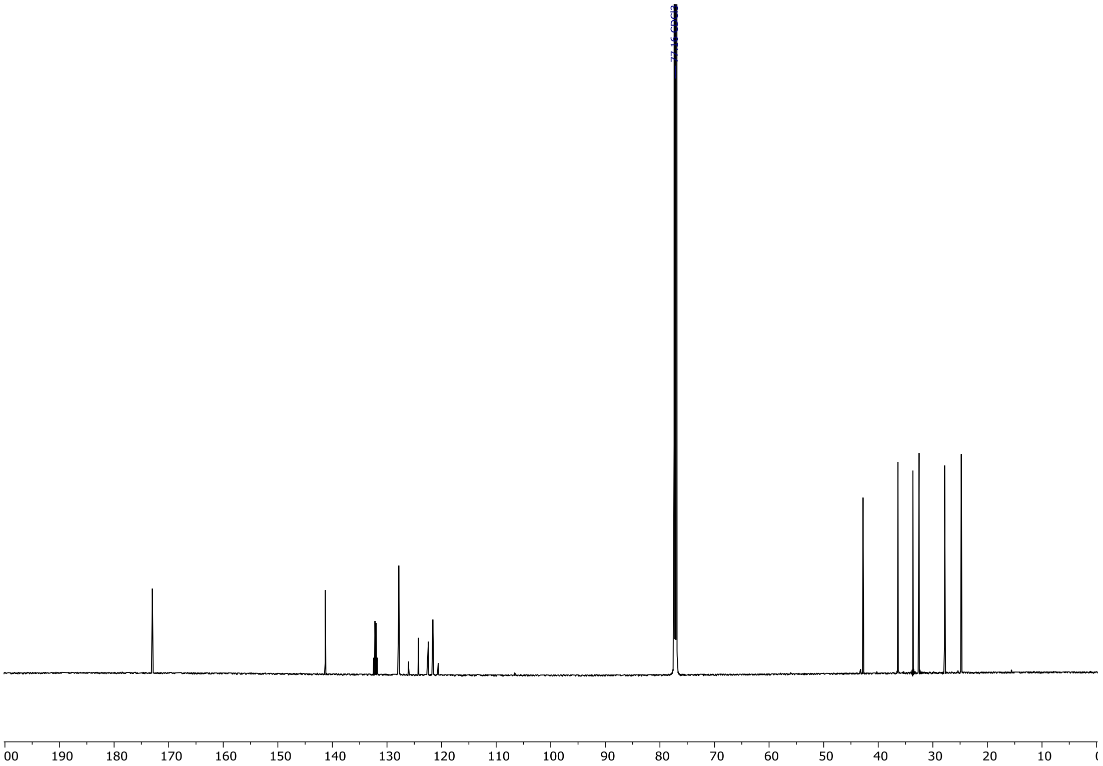

Spectrum S58. ^{13}C NMR spectrum (151 MHz, $CDCl_3$, 298 K) of compound **S29**.

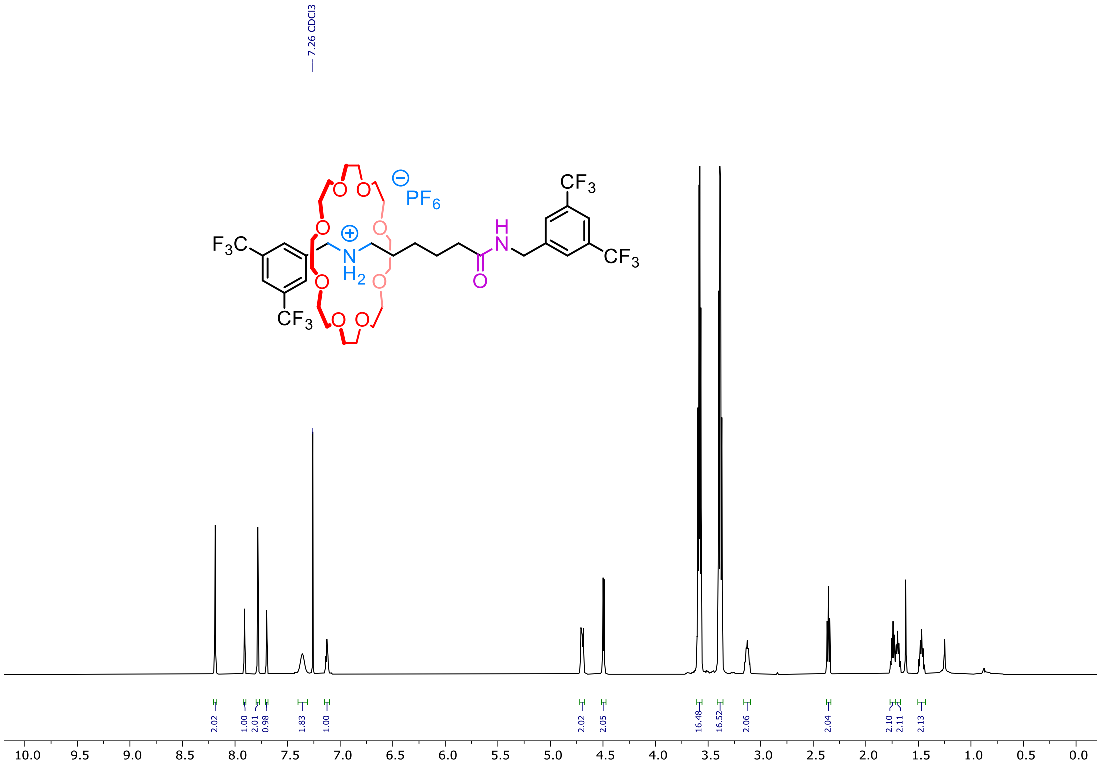

Spectrum S59. ^{1}H NMR spectrum (600 MHz, $CDCl_3$, 298 K) of compound **12**·PF_6.

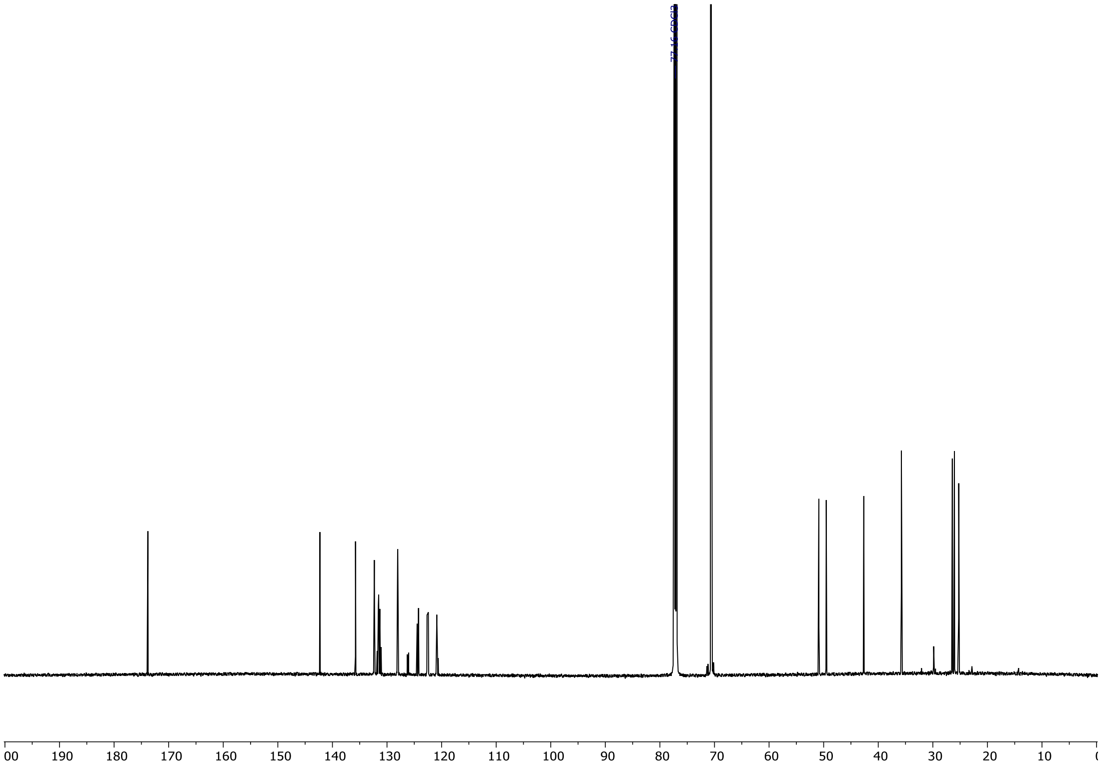

Spectrum S60. ^{13}C NMR spectrum (151 MHz, $CDCl_3$, 298 K) of compound **12**·PF_6.

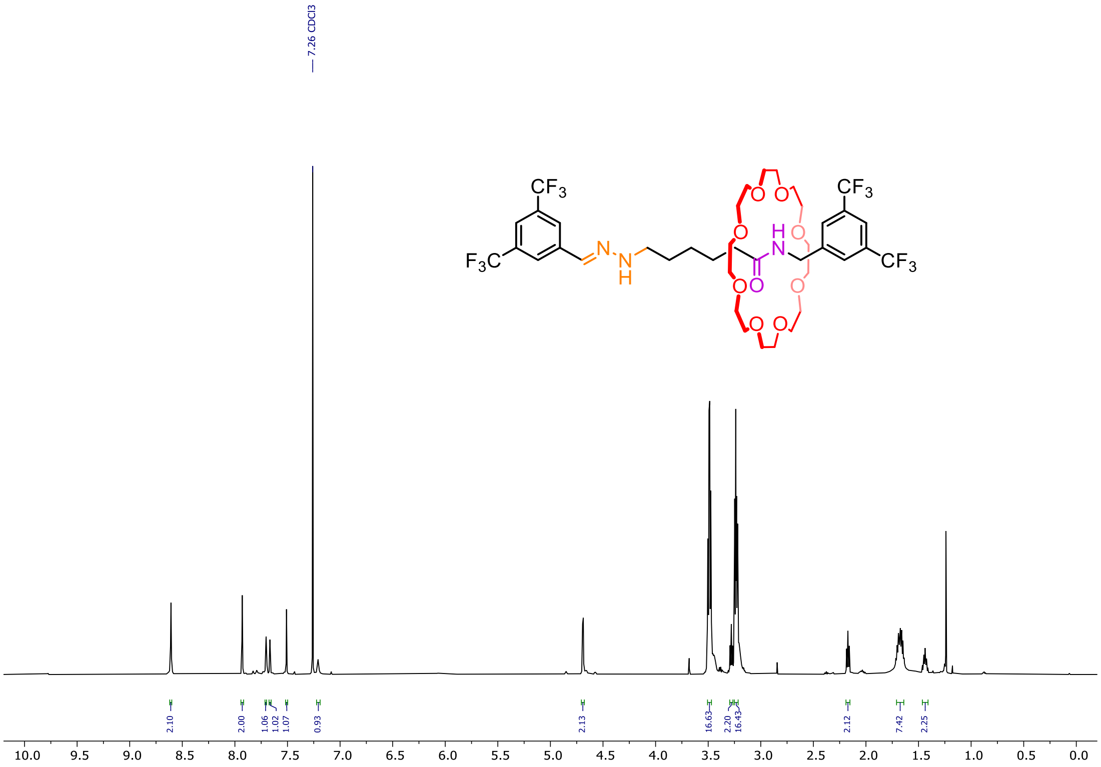

Spectrum S61. ^{1}H NMR spectrum (600 MHz, $CDCl_3$, 298 K) of compound **13**.

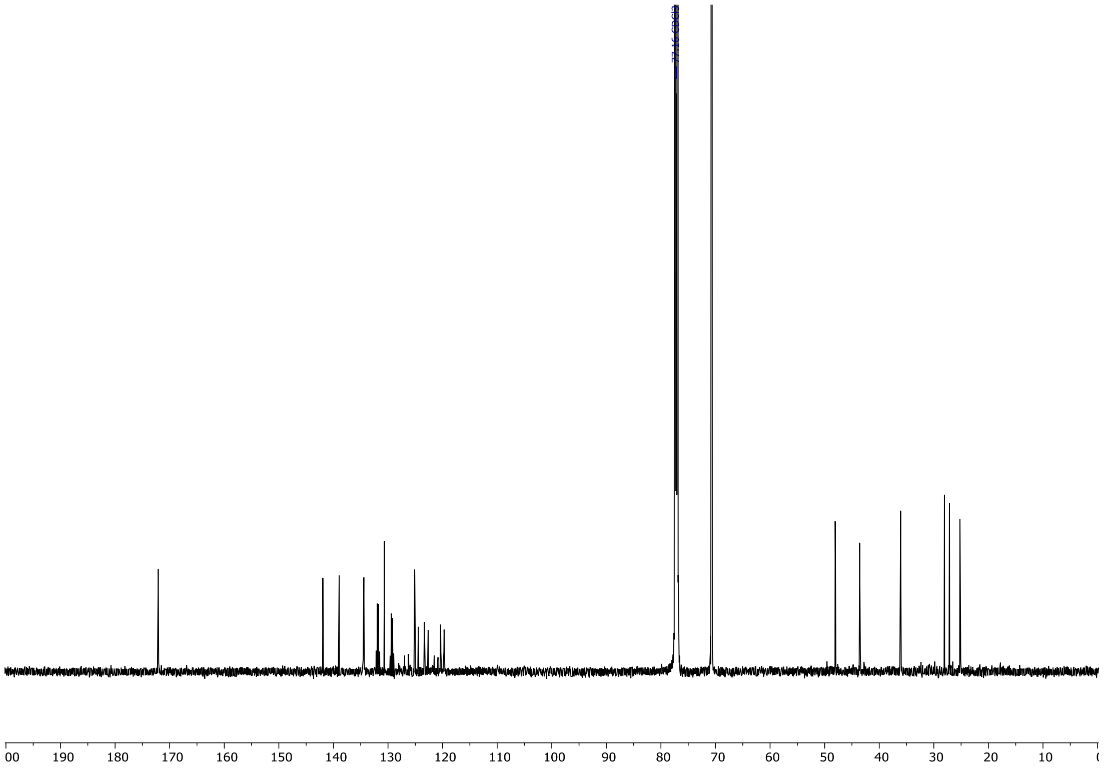

Spectrum S62. ^{13}C NMR spectrum (151 MHz, $CDCl_3$, 298 K) of compound **13**.
